# Computational strategies for copy number variation detection, disease association, and beyond

**DOI:** 10.1186/s13059-026-04189-6

**Published:** 2026-07-10

**Authors:** Amir Hossein Saeidian, Hani Sabaie, Mahdi Akbarzadeh, Hassan Vahidnezhad, Leila Youssefian, Toktam Saadattalab, Haowei Du, Xi Luo, Nichole Marie Owen, Xiaonan Zhao, Joseph Glessner, Hakon Hakonarson

**Affiliations:** 1 Department of Molecular and Human Genetics, Baylor College of Medicine, Houston, TX USA; 2 Center for Applied Genomics, Children’s Hospital of Philadelphia, Philadelphia, PA USA; 3 Department of Medical Genetics, Faculty of Medicine, Tabriz University of Medical Sciences, Tabriz, Iran; 4 Cellular and Molecular Endocrine Research Center, Research Institute for Endocrine Molecular Biology, Research Institute for Endocrine Sciences, Shahid Beheshti University of Medical Sciences, Tehran, Iran; 5 Department of Pediatrics, Perelman School of Medicine, University of Pennsylvania, Philadelphia, PA USA; 6 Division of Genetic and Genomic Medicine, Children’s Hospital of Philadelphia, Philadelphia, PA USA; 7 Department of Dermatology, Perelman School of Medicine, University of Pennsylvania, Philadelphia, PA USA; 8 Department of Pathology, Cytogenetics Laboratory, City of Hope National Medical Center, Duarte, CA USA; 9 Department of Medicinal Chemistry, Faculty of Pharmacy, Tehran University of Medical Sciences, Tehran, Iran; 10 Division of Pulmonary Medicine, Children’s Hospital of Philadelphia, Philadelphia, PA USA; 11 Faculty of Medicine, University of Iceland, Reykjavik, Iceland

## Abstract

Copy number variations (CNVs) are key structural variations that contribute to human genetic diversity, evolution, and disease susceptibility. Advances in sequencing technologies and computational methods have improved CNV detection, yet association studies remain challenged by methodological limitations and a lack of standardisation. This review provides an overview of computational strategies for germline CNV detection and disease association. We highlight the value of CNV analysis for uncovering genetic contributions to complex traits and disease risk and outline an analysis workflow including key benchmarking methods. We also discuss current challenges and future directions for advancing CNV detection and association analysis.

**Supplementary Information:**

The online version contains supplementary material available at https://doi.org/10.1186/s13059-026-04189-6.

## Introduction

**Fig. 1:**
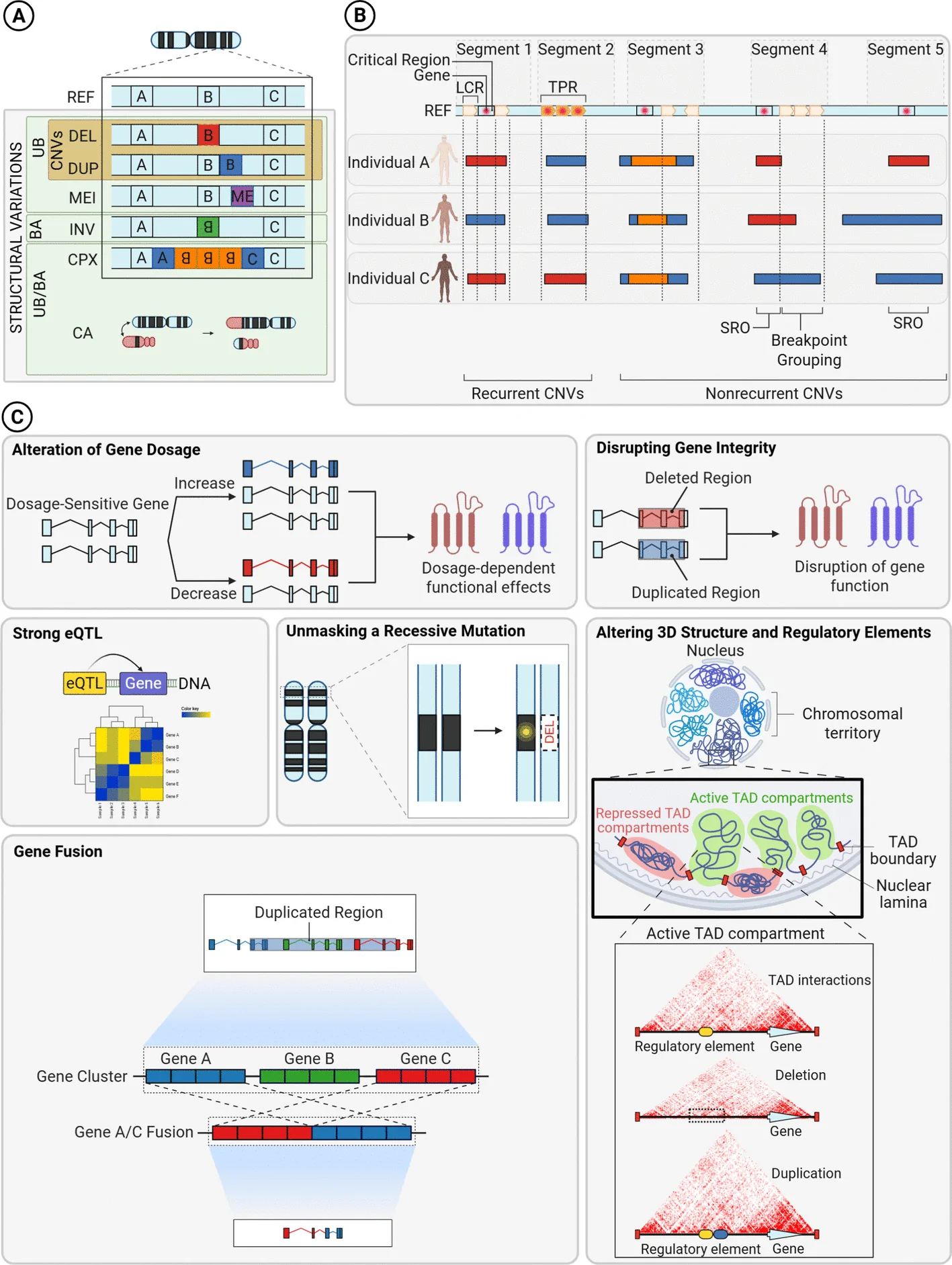
Structural and functional features of copy number variations (CNVs). **A** Structural variations (SVs) are genomic alterations, including deletions (DEL), duplications (DUP), insertions (INS; ≥ 50 bp), including mobile element insertions (MEIs), inversions (INV), complex rearrangements (CPX), and chromosomal abnormalities (CA), including reciprocal translocations and aneuploidy. These include balanced (BA) and unbalanced (UB) types. BA SVs maintain DNA dosage but may disrupt gene structure, whereas UB SVs cause DNA gain or loss. CNVs are SVs that are typically divided into DEL, which reduces the copy number, and DUP, which increases it. Moreover, complex CNV architectures like DUP-triplication (TRP)/INV-DUP configurations can occur. **B** Recurrent and non-recurrent CNV architectures and breakpoint patterns. The reference genome (REF) is divided into segments, with dosage-sensitive genes in critical regions. Low-copy repeats (LCRs) mediate non-allelic homologous recombination (NAHR), causing recurrent CNVs. Tandem paralogous repeats (TPR) can also mediate these rearrangements. CNVs are shown as coloured rectangles (red for DEL, blue for DUP, and orange for inverted TRP structures). Segment 1 contains the critical region gene flanked by LCRs, predisposing it to recurrent CNVs of the same size and content across individuals. Segment 2 shows that recurrent rearrangements are possible within TPRs and alter the copy number of a dosage-sensitive gene located inside the repeat. Segment 3 illustrates non-recurrent DUP-TRP/INV-DUP structures. Segment 4 represents non-recurrent rearrangements that display breakpoint grouping within paralogous repeats, in contrast to the tight clustering seen in recurrent cases. Segment 5 demonstrates non-recurrent rearrangements without any clustering or grouping of breakpoints, resulting in entirely unique CNVs across individuals. The smallest region of overlap (SRO) refers to the minimal genomic segment that is consistently altered among different individuals with overlapping CNVs. Panel B was inspired by the schematic representation of recurrent and non-recurrent CNVs described in [4]. **C** Functional consequences of CNVs. CNVs can affect gene and protein function through gene dosage alterations, disruption of gene structure, changes in regulatory architecture (including topologically associating domains), effects on gene expression, and formation of gene fusions or unmasking of recessive variants. These functional consequences are essential for interpreting the phenotypic impact of CNVs. Created with BioRender.com

Structural variations (SVs) are crucial human genetic variations, typically defined by alterations involving 50 or more DNA nucleotides [1]. These SVs include a broad spectrum of genomic changes, including copy number variations (CNVs), insertions (≥ 50 bp), including mobile element insertions (MEIs), inversions, and chromosomal abnormalities [2, 3] (Fig. 1A). Investigating SVs is crucial for understanding the diversity of human genetics, their role in evolution, and their functional effects (reviewed in Collins and Talkowski [2]).

CNVs, a class of SVs characterised by changes in the number of copies of a particular segment of DNA, are among the most frequently examined and prevalent SVs. Previous studies have shown that insertions and CNVs collectively account for the majority of SVs in the human genome, often exceeding 90% of all detected SVs [2]. However, long-read sequencing (LR-seq) studies have demonstrated that the relative distribution of SV classes can vary across datasets, with insertions and deletions often identified at comparable frequencies. The observed spectrum of SVs is therefore influenced by sequencing technology, analytical methods, and genomic context [5]. CNVs involve deletions or duplications within the genome, typically defined as spanning 50 base pairs or more and often exceeding 1 kb. These variations are fairly prevalent, with approximately 10,000 occurrences per genome, and approximately 800 of these are larger than 10 kilobases [2]. These structural alterations account for a significant portion of genetic diversity, encompassing an estimated 5–10% of the human genome [6, 7].

CNVs at a locus can arise as recurrent or non-recurrent events. Recurrent CNVs are typically mediated by genomic features, such as low-copy repeats, resulting in similar breakpoints across individuals, whereas non-recurrent CNVs exhibit variable breakpoints and structures (Fig. 1B). Beyond structural features, CNVs exert diverse functional effects that are important for downstream interpretation but are not directly part of computational detection workflows. As summarised in Fig. 1C, a primary mechanism is gene dosage alteration, where deletions may result in haploinsufficiency and duplications in triplosensitivity, particularly in dosage-sensitive genes [2, 8]. CNVs can also disrupt gene integrity and alter regulatory landscapes, for example, by perturbing topologically associating domains (TADs) and modifying enhancer-gene interactions [3, 8, 9]. In addition, CNVs can have substantial effects on gene expression and may act as expression quantitative trait loci (eQTLs) [10, 11], or contribute to the formation of gene fusions in the context of complex rearrangements [12]. Deletions may also unmask recessive variants on the homologous chromosome, thereby revealing pathogenic alleles that would otherwise remain silent [8].

**Fig. 2:**
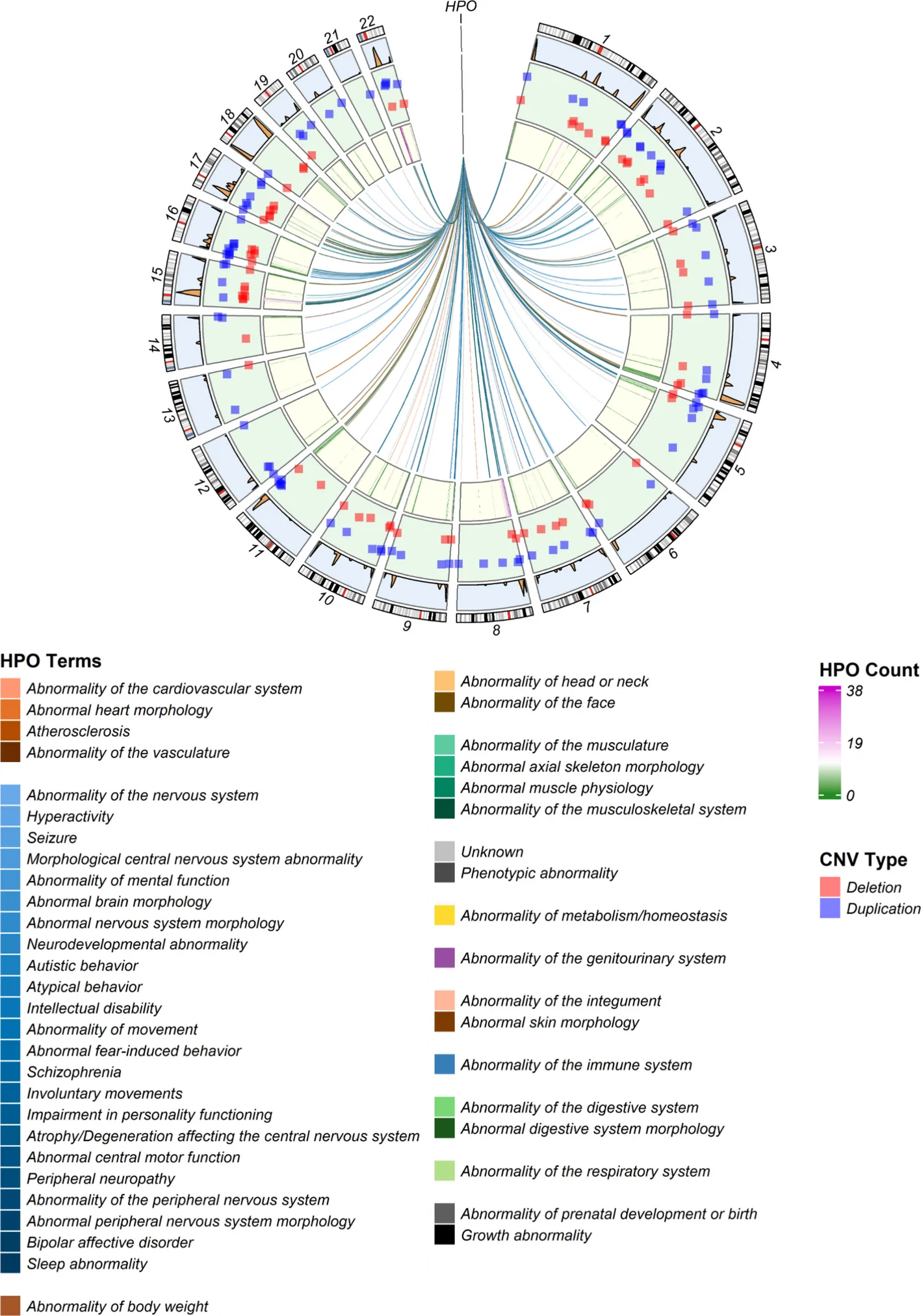
Genome-wide distribution of disease-associated copy number variations (CNVs) and their associated phenotypes. To illustrate the broad impact of CNVs, we visualised data from Collins et al. [14], which showed that large rare CNVs are significantly associated with multiple human diseases and traits across the genome. The study performed a large-scale meta-analysis across multiple cohorts, identifying 163 rare, large CNV loci significantly associated with diverse human diseases and traits, particularly neurodevelopmental and psychiatric disorders. Circos plot displaying chromosomes 1–22 with outer ideograms. The second track shows the density plots of CNV counts along each chromosome. The third track depicts individual CNV loci, with red squares representing deletions and blue squares representing duplication. The fourth track is a heatmap indicating the number of Human Phenotype Ontology (HPO) terms mapped to each locus (low to high intensity). The innermost links connect genomic loci to HPO categories, which are colour-coded as indicated in the figure legend. The figure was generated using the circlize R package [23]

Genomic disorders (GDs) arising from CNVs or genomic rearrangements provide definitive evidence of CNV pathogenicity [13]. Many well-characterised GDs, particularly those mediated by recurrent large CNVs, involve megabase-sized regions encompassing multiple genes [14]. While some of these disorders have a single dominant genetic driver, others follow oligogenic and polygenic models, underscoring the complexity of the genetic architecture and the influence of modifier effects [15–17]. Several recurrent GD CNVs found in the general population can influence physiological traits, such as height, even in the absence of overt clinical disease [18–20]. Beyond classical GDs, CNVs are associated with a broad spectrum of neurodevelopmental and psychiatric conditions, including schizophrenia, autism, and bipolar disorder [14, 21, 22] (Fig. 2). Taken together, CNVs are widely recognised as important contributors to disease susceptibility, and in some cases, they can exhibit large effect sizes approaching those observed in Mendelian disorders. For example, variation at the 22q11.2 locus illustrates this impact, with deletions associated with an approximately 60-fold increase in schizophrenia risk, whereas duplications may reduce risk to approximately 15% of baseline levels [8, 21]. At the same time, many CNVs are incompletely penetrant and show variable expressivity, reflecting the broader complexity of their genetic architecture [2].

Large-scale biobanks are essential for advancing this field. By linking genome-wide CNV discovery with large-scale and broadly characterised phenotypic data, resources such as the UK Biobank enable systematic CNV association analysis across tens of thousands of individuals. These analyses refine penetrance estimates, identify pleiotropic effects across traits, and reveal modifier influences from local and genome-wide genetic backgrounds [8, 18, 24–27]. Advances in sequencing technologies and computational methods have enabled higher-resolution and more sensitive CNV detection at a larger scale, including major contributions from large-scale efforts such as the Human Genome Structural Variation Consortium, which has leveraged LR-seq and haplotype-resolved assemblies to improve the discovery and characterisation of SV [28–32]. However, CNV association studies face several gaps in best practices, with challenges ranging from methodological issues to a lack of standardisation and a robust data infrastructure. These limitations hinder accurate and reproducible CNV detection and association analysis. A primary obstacle is the variability and low consensus in CNV calls across different algorithms and platforms [33–36]. Tool performance is highly context-dependent, with strengths and limitations varying across sequencing technologies, data types, and analytical strategies. As a result, no single algorithm consistently outperforms others across all platforms and use cases [33, 34, 37–41]. With numerous tools available, each yielding different outcomes, selecting the appropriate tool is overwhelming. Most current tool comparisons rely on benchmarks provided by tool developers, potentially introducing bias. Without independent benchmarking, researchers and clinicians risk making suboptimal choices that may compromise the analysis and clinical interpretation [33, 37–40, 42–45]. Furthermore, unlike single nucleotide polymorphism (SNP)-based association analysis, which has standardised tools such as PLINK [46, 47] and REGENIE [48], there is currently no universally adopted or community-standard tool for CNV-based association analysis [8, 49], although ongoing community efforts are actively developing and benchmarking methods in this field. Existing tools often lack user-friendliness, essential reporting features, or fail to integrate necessary analytical steps [49–52].

In this review, we focus on germline CNVs, as their detection, interpretation, and association analyses rely on distinct study designs and computational frameworks compared to somatic CNVs, which are typically investigated in cancer genomics and mosaic contexts. While somatic CNVs have well-established roles in both cancer and non-cancer conditions [40, 53–65], their analysis involves fundamentally different methodological considerations and is therefore beyond the scope of this review. Accordingly, we concentrated on computational approaches for the detection and association analysis of germline CNVs.

We first highlight the value of CNV association analysis in revealing genetic contributions to complex traits that are often missed by SNP-based analysis. We then provide a general workflow for CNV association analysis. We then present the platforms and methods for CNV detection and outline the benchmarking frameworks and challenges of comparing CNV detection tools. Next, we review CNV calling and association analysis tools, focusing on independent benchmarking studies for unbiased performance evaluation. In the absence of independent evidence, we supplemented the discussion with a critical overview of community-recommended tools to provide practical, evidence-based, and context-specific recommendations for researchers and clinicians seeking to select appropriate CNV detection and association approaches. Finally, we conclude by addressing the current challenges and future perspectives of CNV detection and association analyses.

## Unique contributions of copy number variation association studies

CNV association analyses provide insights that go beyond the capabilities of SNP-based methodologies, offering unique opportunities and expanding our understanding of the genetic architecture of complex traits [8, 20, 26, 27, 66]. In some cases, traits such as birth weight, total cholesterol, low-density lipoprotein (LDL) cholesterol, and apolipoprotein B have been shown to be associated with CNV burden, indicating an additional layer of genetic architecture shaped by rare, high-impact CNVs or more common variants with mild effects [27]. There is a growing body of literature on the use of CNV modelling for trait association testing (Additional file 1: Fig. S1, Additional file 2: Table S1), although the majority still rely on SNP arrays, which are limited by their low resolution [8]. Traditional SNP arrays lack the resolution to reliably detect small CNVs and are generally insensitive to the full spectrum of CNVs present across the genome, particularly those that are smaller but potentially impactful [67–69]. Moreover, the substantial overlap between genomic CNV loci and genome-wide association study (GWAS) signals, as well as their enrichment in recombination hotspots, suggests that CNVs may explain a portion of the missing heritability that cannot be accounted for by SNPs alone [70].

SNP and CNV signals can influence the measurement and interpretation of each other. In array-based genotyping, SNP-calling algorithms typically assume a fixed diploid copy number state and have limited ability to capture underlying CNV variation. Consequently, unmodelled CNVs can lead to incorrect genotype assignments and apparent deviations from the Hardy–Weinberg equilibrium. Conversely, ignoring SNP information during CNV calling may overlook allele-specific gains and losses and limit the ability to leverage the linkage disequilibrium (LD) between CNVs and nearby SNPs [71]. Many CNVs, particularly rare or structurally complex ones, are poorly tagged by SNPs, which means that standard approaches are unable to detect their effects [8, 72–78]. One major reason is that CNVs, particularly recurrent ones, can arise repeatedly at specific genomic loci due to elevated local mutation rates, resulting in multiple independent events with similar functional consequences. The de novo mutation rate for CNVs has been estimated at approximately 0.2 events per individual, reflecting the propensity of certain genomic regions to undergo recurrent structural changes. Consequently, CNVs are often not consistently associated with specific haplotypes, making their tagging by SNPs challenging [72–75, 79]. Consequently, the LD between CNVs and nearby SNPs is often weak or variable, particularly in repetitive sequence contexts, where elevated CNV mutation rates and increased genotyping errors further destabilise LD. Furthermore, multiallelic CNVs (mCNVs), which have a wide range of copy numbers beyond the diploid expectation, can break typical LD patterns and generate population-specific runaway haplotypes that cannot be tagged by short variants. These mCNVs also tend to have a lower average observed LD with nearby SNPs [2, 72, 78, 80, 81]. The rarity and structural diversity of certain CNVs also limit the statistical power of standard SNP-based association methods to detect or accurately model them, while complex CNV rearrangements pose additional challenges for accurate interpretation and tagging [47, 52, 66]. According to the Wellcome Trust Case Control Consortium, 79% of common CNVs (minor allele frequency (MAF) > 10%) can be tagged by nearby SNPs with high LD. However, only approximately 22% of less common CNVs (those with MAF below 5%) are well-tagged by SNPs [77], and this tagging is shown to be even less effective in populations of African or African American descent due to lower LD between SNPs and CNVs [78]. Collectively, these factors make strong SNP-CNV tagging uncommon when considering the full spectrum of CNVs, particularly rare, complex, and multiallelic variants across the genome.

Consequently, CNV-specific association studies play a crucial role in uncovering genetic signals that are undetectable in SNP-based association studies. For instance, in a recent study analysing fine-mapped CNV association regions, 17% (23 out of 133 regions) were classified as CNV-only, highlighting the unique contribution of CNV analysis to genetic discovery [66]. CNV-only associations refer to genetic signals that can be detected exclusively through CNV GWAS but not by SNP GWAS. Examples of CNV-only associations illustrate the added value of CNV GWAS. For instance, CNVs affecting *SPDYE1*, *SPDYE6*, and *POLR*-related genes have been associated with chronotype [66]. Notably, these genes had not previously been linked to chronotype by SNP-GWAS in the UK Biobank, suggesting that CNVs in these regions may influence an individual’s sleep patterns in a dose-dependent manner [66]. Additional CNV-only associations have been found, such as a region at the *ZDHHC11B* gene for the forced expiratory volume (FEV)/forced vital capacity (FVC) ratio and another at the *CDK11A* gene associated with standing height [66].

The mechanisms by which CNVs exert their effects can also differ fundamentally from those of SNPs, as CNVs can act through changes in dosage that may have opposite or identical phenotypic consequences that are not fully captured by SNP-based models [20, 26]. CNV analysis also informs our understanding of gene function and dosage sensitivity, generating or corroborating hypotheses about how specific genes influence biological processes and disease risk [20, 26, 27]. By studying pathogenic CNVs in the general population rather than solely in clinical cohorts, researchers can better characterise the full range of phenotypic outcomes, from severe early onset conditions to mild or asymptomatic manifestations. This aligns with a model of variable expressivity and incomplete penetrance, providing a more nuanced view of genotype–phenotype relationships [26, 27, 82–84].

From a clinical perspective, CNV association studies generate morbidity maps that help clinicians anticipate and monitor potential outcomes for carriers of specific CNVs [26, 85, 86]. While loss-of-function variants in genes such as *BRCA1* and *LDLR* are known to increase disease risk irrespective of variant type, CNV association studies provide complementary insights by characterising the population-level impact of these alterations. In particular, they enable the assessment of penetrance, variable expressivity, comorbidities, and overall CNV burden in large cohorts, thereby refining our understanding of disease risk and clinical outcomes [26]. These insights improve risk stratification and support clinical decision-making, particularly for recurrent or multi-gene CNVs.

## General workflow for copy number variation association analysis

**Fig. 3:**
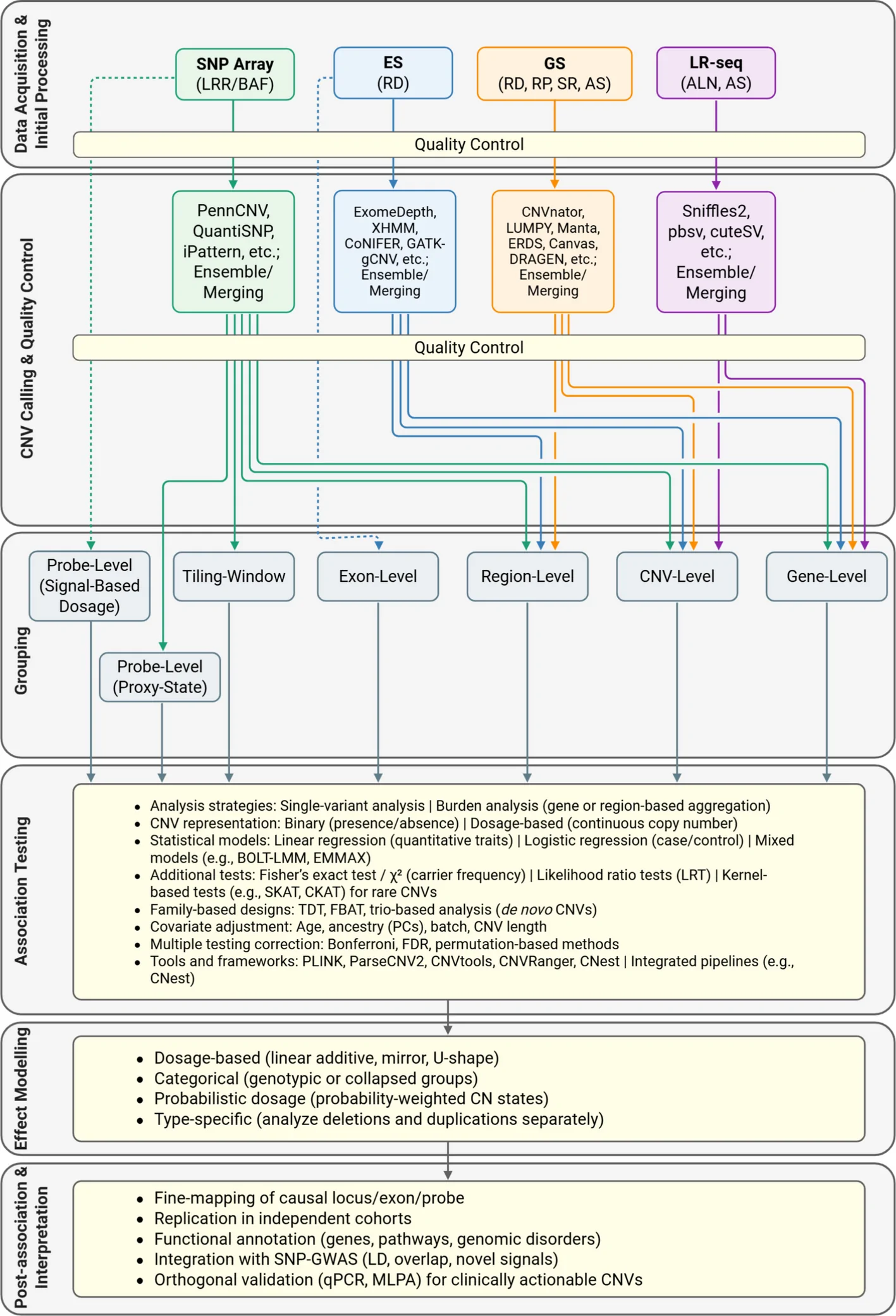
Workflow of copy number variation (CNV) association analysis. The process begins with data acquisition from multiple platforms, including SNP arrays, short-read exome sequencing (ES), short-read genome sequencing (GS), and long-read sequencing (LR-seq). ES is represented here by read depth signals, which constitute its primary input for CNV detection. Following initial normalisation and quality control (QC), CNV calling is conducted using platform-specific CNV callers or ensemble/merging strategies. Some callers listed in this figure, such as DRAGEN, represent comprehensive end-to-end analysis platforms applicable across multiple sequencing modalities, including both ES and GS, rather than standalone CNV detection algorithms. Notably, there are two exceptions: probe-level analyses, which utilise normalised signal intensities from SNP arrays directly, and exon-level analyses, which test the normalised read depth per exon in ES. These exceptions bypass CNV calling by directly engaging with the quantitative signals. After CNV calling (or signal-level approaches in these exceptions), post-calling QC is implemented to ensure reliability by filtering out low-quality events and regions with noise. Detected CNVs are then categorised at various resolutions for association testing, including probe-level (signal or proxy-state), tiling-window, exon-level, region-level (CNVRs), CNV-level, or gene-level. Although other grouping schemes could be theoretically envisioned, these levels represent the approaches most frequently adopted in practice. Statistical association testing is conducted using standard regression models (linear, logistic, mixed models), burden tests, or kernel-based approaches, supported by an expanding array of dedicated CNV association tools, such as PLINK [46, 47], ParseCNV2 [49], CNVtools [87], CNVRanger [88], and CNest [66]. Effect modelling frameworks (dosage-based, categorical, probabilistic, or type-specific) and post-association analysis steps (fine-mapping, replication, orthogonal validation, integration with SNP-GWAS, pathway analysis, and clinical annotation) further refine biological and clinical inferences. Created with BioRender.com

The workflow for CNV association analysis identifies CNVs that are significantly associated with human traits and diseases and interprets their biological and clinical relevance (Fig. 3).

**Fig. 4:**
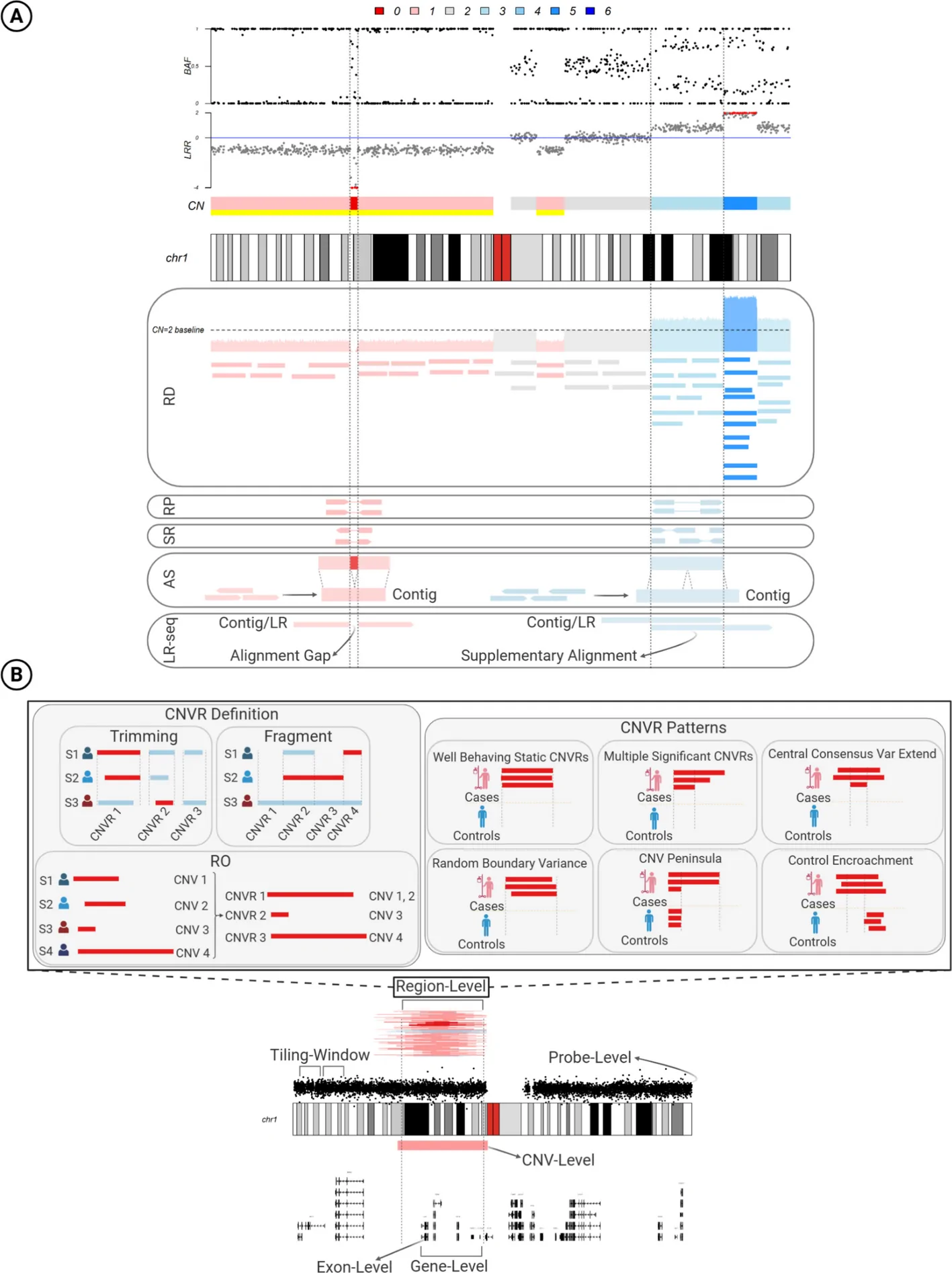
Copy number variation (CNV) signal representations and CNV region (CNVR) definition strategies. **A** Illustrations of CNV signal representation across various platforms are provided. For SNP arrays, normalised metrics, such as B-allele frequency (BAF) and log R ratio (LRR), are presented alongside copy number states. In short-read sequencing-based methodologies, pertinent CNV evidence encompasses read depth (RD), discordant read pairs (RP), split reads (SR), assembly (AS), and long-read sequencing (LR-seq), using either alignment- or assembly-based approaches, where deletions and duplications are identified through alignment gaps or supplementary alignments. These signals provide complementary evidence of CNVs, with RD reflecting dosage changes, RP indicating abnormal insert sizes or orientations, and SR enabling breakpoint resolution; these same signatures are also used during manual inspection (e.g., in IGV) to distinguish true variants from technical artefacts. Additional technical details on detection methodologies and signal processing are available in Additional file 3: Note S1 [2, 28, 29, 56–61, 89–103] and Additional file 3: Note S2 [28, 94, 103–132]. **B** Schematic representation of CNVR definitions and common CNVR patterns observed in case–control analyses. Overlapping CNV calls across individuals can be consolidated into CNVRs using various methods, including trimming, reciprocal overlap (RO), and fragment-based techniques [50]. Given the variability in CNV size and breakpoints, distinct CNVR patterns may emerge in case–control data. Well-behaved CNVRs show highly concordant CNV boundaries across samples. Random boundary variance reflects minor stochastic variation in breakpoints. Multiple significant CNVRs represent distinct clusters of overlapping CNVs. A CNV peninsula denotes a small case-specific extension with limited probe support adjacent to a shared region. Central consensus with variable extension indicates a shared core CNV with variable flanking boundaries. Control encroachment occurs when CNVs in controls overlap regions defined primarily by cases [52]. Parts of the schematic were generated using the R package karyoploteR [133]. Created with BioRender.com

The key signal representations used for CNV detection across platforms and strategies for defining CNV regions (CNVRs) are illustrated in Fig. 4.

### Data acquisition and initial processing

The workflow begins with data acquisition from array- or sequencing-based platforms. A detailed overview of the platforms and methodologies for CNV detection is provided in Additional file 3: Note S1. For array-based datasets, raw signals are processed to obtain the log R ratio (LRR), which represents the relative signal intensity, and the B-allele frequency (BAF), which shows the B allele proportion at each locus. For sequencing-based datasets, read depth profiles and alignment signatures are extracted, with split reads or discordant read pairs potentially included based on the CNV calling algorithm. This stage includes normalisation and QC to reduce technical artefacts before CNV detection [5, 19, 20, 24, 26, 66, 86, 134–141].

### CNV calling

After preprocessing, CNV calling algorithms are applied to infer individual copy number states (see Additional file 3: Note S2 and Additional file 3: Note S3 [103, 113, 120, 142–156] for details on CNV detection strategies and segmentation approaches). CNV detection performance depends strongly on the underlying platform, with key differences in resolution, breakpoint accuracy, and sensitivity to CNV size. Different tools employ statistical models to integrate various signal features to call CNVs (Additional file 1: Table S2 [66, 105–107, 109–114, 119–122, 125–127, 129, 131, 142, 144, 145, 147, 149–151, 157–207] and Additional file 1: Figs. S2-S8 [208]). To increase reliability, it is common to use multiple algorithms in parallel [28].

#### Approaches and tools for copy number variation detection via ensemble and merging strategies

Ensemble and merging approaches for CNV detection are designed to overcome the limitations of individual calling tools, such as high false-positive rates, low concordance, and incomplete detection [28]. Because no single CNV detection method consistently delivers the best results across various contexts, combining the strengths of multiple tools has emerged as a promising strategy for CNV detection.

These approaches can be broadly categorised based on their primary strategies for combining and refining the variant calls. These ensemble and merging strategies have been developed across different platforms, including SNP arrays, exome sequencing (ES), genome sequencing (GS), and LR-seq, and their applicability is often platform specific. One category is simple consensus or overlap-based approaches, which rely on the direct overlap of variant calls or require a minimum number of callers to agree on a variant to define high-confidence calls. Although straightforward, these methods may not always achieve the highest precision or recall. For example, HugeSeq, developed for GS data, identifies high-confidence SVs and CNVs as those detected by two or more algorithms with at least 50% reciprocal overlap, integrating outputs from tools such as BreakDancer, Pindel, CNVnator, and BreakSeq using BEDtools [183]. NextSV, designed for SV detection from low-coverage LR-seq data, integrates the results from multiple SV callers to improve robustness. It produces two call sets: a sensitive set (union of all calls) that maximises recall, and a stringent set (intersection of calls) that prioritises precision. For instance, in the case of deletions, calls are combined based on reciprocal overlap thresholds, which are typically 50% or higher [173]. SURVIVOR is another widely used tool that filters and merges SVs based on user-defined criteria, such as coordinate distance, SV type, and chromosome, and supports tools such as Delly, LUMPY, Pindel, and cn.MOPs [176].

Although combiSV also combines outputs from multiple callers, it is not a simple overlap-based method. It is a recent ensemble tool specifically designed to improve SV detection from LR-seq data by combining the outputs of multiple SV detectors. It takes variant call format (VCF) outputs from up to six different tools, namely cuteSV, pbsv, Sniffles, NanoVar, NanoSV, and SVIM, and integrates their calls to produce a consensus SV call set with increased recall and precision. Unlike simple overlap-based methods, combiSV uses caller-specific prioritisation to select the most accurate information for each SV parameter (e.g., position, length, and genotype) based on benchmarking results, placing it in the category of advanced ensemble methods. It also allows users to set thresholds for the minimum number of supporting callers and read the coverage for each variant [171].

A more sophisticated category involves machine learning and statistical integration approaches that use data-driven models to assign weights or integrate features across variant calls, leading to better accuracy and a reduced number of false positives. CN-Learn, designed for ES data, is a random forest-based framework that integrates CNV calls from multiple algorithms, such as CANOES, CODEX, XHMM, and CLAMMS, using features such as GC content and mappability, trained on a subset of validated CNVs [193]. FusorSV, which targets GS data, is part of the Structural Variation Engine and uses a data mining approach to assess tool performance against a truth set. It combines calls using a mutual exclusion strategy that minimises false positives while maximising sensitivity [161].

Another group of tools falls under advanced merging with refinement or validation pipelines, which not only combine calls but also refine breakpoints, re-genotype variants, and validate them using additional evidence. For instance, EnsembleCNV detects and genotypes CNVs from SNP array data using a two-phase pipeline involving initial detection and a re-genotyping step that uses local likelihood models to refine the boundaries [131]. MetaSV, which is optimised for GS data, merges calls from multiple SV detection tools using intra- and inter-tool merging strategies, integrates local assembly via SPAdes, refines breakpoints, performs genotyping, and annotates variants [189]. Parliament2, designed for the scalable analysis of GS data, integrates multiple SV callers, uses SURVIVOR for merging, SVTyper for validation, and assigns a quality score to each SV based on supporting evidence [205]. SVMerge, also developed for GS data, takes a modular approach by integrating SV calls from various tools and refining them using local de novo assembly, making it extensible to future SV calling tools [201].

Ensemble and merging algorithms improve CNV detection but face key limitations, including a lack of standardisation, variable sequencing coverage, dependency on specific algorithm combinations, inconsistent benchmarking, and immature standalone tools. For an overview of ensemble algorithms for SV detection, readers should refer to the detailed review by Ho et al. [28].

#### Overview of benchmarking

Benchmarking SVs, including CNVs, is a crucial process in genomic research that is designed to evaluate and validate the accuracy of variant calling methods. The constant progress in genomic sequencing technologies has led to enhanced detection of SVs, particularly with the advent of LR-seq, which allows for the refined characterisation of these variants [42].

Benchmarking consists of four core components. The first is the establishment of truth data [42], a gold standard or ground truth dataset (such as those produced by the Genome in a Bottle Consortium, GIAB [209]), to which the analytical results can be compared. These truth sets, also referred to as high-confidence or ground truth call sets, are typically generated through the integration of multiple sequencing technologies, variant calling methods, and extensive manual curation to minimise platform-specific biases [42]. However, widely used resources such as those from the GIAB consortium are derived from a limited number of individuals and may not fully capture the diversity or spectrum of clinically relevant CNVs across populations [42]. Multiple benchmark datasets are available for assessing SVs, including CNVs (reviewed by Majidian et al. [42]). An important complement to GIAB-derived truth sets is the Platinum Pedigree resource [210], which leverages Mendelian inheritance across a multi-generation pedigree (CEPH-1463) sequenced with PacBio HiFi, Illumina, and Oxford Nanopore Technologies. By tracking haplotype transmission within the family, this resource validates variant calls genome-wide, including single nucleotide variants, indels, tandem repeats, and SVs, across difficult genomic regions often excluded from conventional truth sets, prioritising sensitivity and completeness over specificity. The second component involves specific tools and algorithms used to align and compare analytical outputs with truth data. Third, performance metrics are employed to comprehensively assess concordance and diagnostic accuracy using standard statistical measures. These include counts such as true positives (TP), false positives (FP), true negatives (TN), and false negatives (FN), from which key indicators are derived. Sensitivity (or recall) measures the proportion of actual positives correctly identified ($\frac{\mathrm{T}\mathrm{P}}{\mathrm{T}\mathrm{P}+\mathrm{F}\mathrm{N}}$), whereas specificity measures the proportion of actual negatives correctly identified ($\frac{\mathrm{T}\mathrm{N}}{\mathrm{T}\mathrm{N}+\mathrm{F}\mathrm{P}}$). Precision, also referred to as the positive predictive value (PPV), indicates the proportion of predicted positives that are truly positive ($\frac{\mathrm{T}\mathrm{P}}{\mathrm{T}\mathrm{P}+\mathrm{F}\mathrm{P}}$). Conversely, the negative predictive value (NPV) shows the proportion of predicted negatives that are truly negative ($\frac{\mathrm{T}\mathrm{N}}{\mathrm{T}\mathrm{N}+\mathrm{F}\mathrm{N}}$). To balance precision and recall, the F1 score, calculated as the harmonic mean of these two metrics ($\frac{2\times (\mathrm{P}\mathrm{r}\mathrm{e}\mathrm{c}\mathrm{i}\mathrm{s}\mathrm{i}\mathrm{o}\mathrm{n}\times \mathrm{R}\mathrm{e}\mathrm{c}\mathrm{a}\mathrm{l}\mathrm{l})}{\mathrm{P}\mathrm{r}\mathrm{e}\mathrm{c}\mathrm{i}\mathrm{s}\mathrm{i}\mathrm{o}\mathrm{n}+\mathrm{R}\mathrm{e}\mathrm{c}\mathrm{a}\mathrm{l}\mathrm{l}}$), is used as a single metric reflecting overall performance [42]. Finally, effective benchmarking necessitates a clear representation of results, which is commonly achieved through visualisations and comprehensive reports designed to convey findings in an interpretable manner [211].

One of the key challenges in SV/CNV benchmarking is that the same genomic alteration can be reported differently by various tools, often with varying breakpoint coordinates, reference alleles, and event descriptions, complicating direct cross-method comparisons [42]. Furthermore, the selection of comparison parameters critically impacts benchmarking outcomes. Overly permissive SV-matching thresholds may cause over-merging of distinct variants, artificially inflate performance metrics, introduce incorrect annotations, falsely classify unique SVs as shared, reduce observed allelic diversity, and overestimate allele frequencies. In contrast, overly stringent thresholds can underestimate performance, miss valid annotations, and reduce the statistical power of downstream analyses [212, 213]. CNV detection is particularly challenging in repetitive genomic regions, where variants are frequently mediated by repetitive elements that complicate alignment and variant resolution [4]. Although advances in LR-seq have improved the characterisation of these regions, substantial challenges remain in establishing reliable truth sets and comparing variant calls, primarily due to breakpoint degeneracy and the existence of multiple equally valid breakpoint placements [29, 42, 214]. Consequently, many benchmark datasets deliberately exclude highly repetitive regions [209, 215, 216]. Despite these limitations, truth sets have been highly effective in several contexts, including standardising performance evaluation across tools using metrics such as precision and recall, benchmarking variants in structurally complex but medically relevant regions, and enabling the development of assembly-based benchmarking approaches (e.g. TT-Mars), which improve evaluation in regions where coordinate-based comparisons are unreliable [42, 214]. Finally, the heterogeneity of output formats produced by different variant calling tools necessitates rigorous standardisation and normalisation prior to comparative analysis [42].

Benchmarking tools are essential frameworks in genomics that enable researchers to objectively evaluate and compare the performance of SV and CNV detection methods using standardised reference datasets and curated truth sets.

Truvari is widely recognised for benchmarking SV callsets by computing the precision, recall, F1-score, and genotype concordance through rigorous comparisons of test callsets with validated benchmark sets. It supports flexible matching criteria, including breakpoint proximity, size similarity, and sequence similarity, making it suitable for both high-resolution and less precise SV calls [213].

SVanalyzer SVbenchmark employs advanced sequence-based matching algorithms, enabling a robust assessment of SV calls with differing representations, particularly in complex or repetitive genomic regions, and improves accuracy even for variants with imprecise breakpoints or those within tandem repeats [209].

TT-Mars introduces a distinct benchmarking paradigm by evaluating SV calls against high-quality haplotype-resolved genome assemblies rather than solely against conventional truth sets. Instead of matching a predefined set of calls, TT-Mars assesses whether the sequence content implied by each SV call aligns with the actual sequence in the assembled haplotypes, making it especially valuable in regions lacking curated truth sets or where variant representation is ambiguous owing to genomic complexity [214].

CNVbenchmarkeR [217] and its enhanced successor, CNVbenchmarkeR2 [38], are comprehensive R-based frameworks for systematically benchmarking CNV calling tools using targeted next-generation sequencing gene panel data. CNVbenchmarkeR automates the execution and evaluation of multiple CNV callers, including DECoN, CoNVaDING, panelcn.MOPS, ExomeDepth, and CODEX2, using validated datasets with gold-standard confirmation via multiplex ligation-dependent probe amplification (MLPA) or array-based comparative genomic hybridisation (aCGH). It calculates an extensive range of performance metrics, including sensitivity, specificity, PPV, NPV, and F1-score, and supports dataset-specific parameter optimisation [217]. CNVbenchmarkeR2 extends these capabilities to benchmark a broader set of CNV tools, such as Atlas-CNV, ClinCNV, CNVkit, and GATK-gCNV, and allows the systematic exploration of over 100 tool parameters, generation of detailed graphical reports, and evaluation of meta-caller strategies that combine the results from multiple tools [38]. Both frameworks are designed to be user-friendly and adaptable, enabling research and clinical laboratories to benchmark CNV detection tools directly using their datasets.

witty.er (https://github.com/Illumina/witty.er) is a dedicated benchmarking tool for large variants that is explicitly designed to assess the performance of SV and CNV callers. Developed by Illumina, it compares query VCFs to truth VCFs for multiple SV types, applies flexible positional and genotype matching criteria, and produces detailed benchmarking statistics with annotated VCFs. Functionally, it is analogous to hap.py for small-variant benchmarking, making it a central resource for performance assessment in large variant calling.

svclassify serves as a foundational resource by enabling the creation of high-confidence benchmark SV call sets. Rather than acting as a benchmarking evaluator, it integrates multi-platform sequencing evidence and applies machine learning algorithms to classify candidate SVs as likely true- or false-positive. The resulting high-confidence SV sets serve as truth sets for downstream benchmarking, ensuring reliable performance evaluation and supporting the reproducibility of SV benchmarking studies [218].

Supporting tools such as SURVIVOR play complementary roles in benchmarking workflows. SURVIVOR can simulate SVs for benchmarking, merge and filter SV callsets, and generate consensus or simulated truth datasets for evaluation, although it does not produce standardised benchmarking reports [176].

By offering standardised workflows for metric calculation, parameter optimisation, and cross-tool comparison, and by integrating with auxiliary tools for callset preparation, simulation, and validation, these benchmarking solutions serve as foundational elements for the development of CNV tools, method selection, and validation of clinical pipelines in both research and diagnostic settings.

#### Insights from independent benchmarking studies of copy number variation callers

Bioinformatic algorithms are essential for CNV detection across sequencing and microarray platforms. Because benchmarking results vary substantially depending on the data type, CNV size, and study design, findings are best interpreted in a platform-aware context. With a wide range of algorithms available, this review synthesises findings from independent benchmarking studies to provide an unbiased overview of tool performance across different contexts. Detailed platform-specific benchmarking results and tool comparisons are provided in Additional file 3: Note S4 [33–35, 37–39, 41, 43–45, 124, 162, 197, 217, 219–229].

Overall, systematic benchmarking of independent CNV callers across various technologies has revealed that no single tool or approach is universally optimal for every experimental context, CNV size, or type. Rather than employing a simplistic overlap strategy, the iterative merging of top-performing tool pairs can enhance both accuracy and sensitivity. However, not all combinations are advantageous, underscoring the need for systematic benchmarking to identify synergistic combinations. Therefore, achieving robust and accurate CNV detection requires a multi-layered strategy involving careful selection and parameter tuning of tools, often necessitating the intelligent combination of multiple callers and rigorous post-processing steps. Key steps include optimising parameters, such as window size, probe selection, caller-aligner pairing, and reference set composition, as well as removing recurrent artefacts through custom filtering and applying quality control thresholds. Comprehensive post-processing and quality checks, such as depth of coverage analysis, uniformity assessment, and outlier detection, are critical for minimising false positives and improving confidence in detected CNVs [28, 35–39, 41, 43, 44, 197, 217, 226–229].

Crucially, computational predictions alone are insufficient for high-confidence CNV calls, particularly for rare, small, and clinically relevant variants. All such findings should be validated using orthogonal methods, ranging from visual inspection and Binary Alignment Map (BAM) review to quantitative PCR (qPCR), MLPA, or array-based approaches before being reported or interpreted clinically. Incorporating post-calling steps, such as genotyping refinement, confidence scoring (potentially using machine learning or Bayesian models), and integration of haplotype or alternative reference information, can further enhance the reliability of CNV calls. Finally, practical considerations, such as workflow reproducibility (e.g., through containerisation and workflow managers), user-friendliness, integration with existing clinical or diagnostic pipelines, and laboratory bioinformatics capacity, are equally important for successful CNV analysis [33, 34, 36–39, 43–45, 197, 217, 220, 222, 225, 226, 229]. While these approaches are effective for detecting CNV events, it is important to distinguish between CNV discovery and genotyping. Association studies, particularly those involving common or multiallelic CNVs, require accurate estimation of copy number states across cohorts, as implemented in cohort-based and mixture-model approaches, such as CNest and GenomeSTRiP [66, 230].

### Quality control and pre-processing

QC is a critical step, both before and after CNV calling, to ensure sample- and variant-level reliability prior to downstream analysis. At the pre-calling stage, individuals with low genotyping rates, outlier LRR or BAF metrics, sex discordance, or abnormal coverage profiles (for sequencing data) are excluded [21, 66, 134, 136–141, 231–235]. Additional corrections, such as GC content adjustment to mitigate hybridisation bias and principal component analysis (PCA) to remove batch effects, are also applied at this stage [20, 66, 86, 134–141, 231]. At the post-calling stage (or equivalently, during variant-level QC for signal-based analyses), low-quality loci are removed. For CNV calls, this includes excluding samples with excessive CNV calls (suggestive of poor DNA quality), filtering events in noisy or polymorphic loci, and removing calls that fail the minimum thresholds for probe density, read depth, or length. For signal-based analyses (e.g., probe- or exon-level association), this includes excluding units with poor coverage, extreme variance, or low mappability. Adjacent CNV calls in the same individual may be merged to prevent over-fragmentation, and problematic genomic regions prone to spurious calls or somatic variation, such as centromeres, telomeres, immunoglobulin loci, T-cell receptor regions, segmental duplications, and low-copy repeats, are routinely excluded [20, 21, 76, 86, 134–141, 231, 232, 236–238]. As discussed in the previous section, high-confidence CNVs are typically defined by concordance across multiple detection methods, probabilistic quality scores, or independent experimental validation [19, 20, 26, 76, 134, 236, 238]. In addition, visual inspection is a critical quality control step used to validate CNV calls and filter out potential artefacts. Researchers typically examine several key characteristics during this process, including signal intensity patterns and allele frequency distributions in array-based data (e.g., LRR and BAF), as well as read depth profiles, split-read and discordant read pair signals, and local mapping quality in sequencing data, often visualised using genome browsers such as Integrative Genomics Viewer (IGV). In addition, genomic context is evaluated using resources such as UCSC Genome Browser, for example, to assess proximity to repetitive elements, segmental duplications, or low-mappability regions, which may indicate potential artefacts [20, 24, 26, 27, 86, 135, 139, 232, 238, 239].

### Copy number variation grouping, statistical association testing, and effect modelling

Approaches to CNV association analysis can be organised hierarchically based on three major components: (i) strategies for grouping CNVs, (ii) statistical frameworks for association testing, and (iii) effect modelling schemes for representing CNVs within a statistical model. The choice at each level should be guided by the study design, sample size, variant frequency, expected effect size, and underlying biological hypotheses.

At the grouping stage, CNVs can be analysed at different levels of granularity. In probe-level analysis, individual probes on genotyping arrays are used as proxies for copy number, with the estimated copy number at each probe typically entered into regression models as a continuous (linear dosage) variable. This eliminates the need for complex CNV overlap criteria, making it efficient for large-scale array-based studies [19, 20, 27]. Gene-level analysis aggregates rare CNVs based on the genes they overlap, especially those predicted to have a functional impact (e.g., loss-of-function), thereby increasing the statistical power [18, 20, 138, 240]. Importantly, the criteria used to assign CNVs to genes vary widely across studies and can substantially influence the association results. These range from simple intersection-based definitions, such as any overlap (≥ 1 bp) [21, 26, 135], to functionally informed approaches requiring overlap with specific elements (e.g., coding exons, splice sites, or UTRs) [27, 241], with exon-centric definitions commonly used for genic burden analyses [21, 136, 238, 239]. Some approaches extend gene boundaries by incorporating flanking regions (e.g., 10–50 kb) to capture potential regulatory effects [18, 76]. More stringent strategies include proportional or reciprocal overlap thresholds (e.g., ≥ 50%), minimum overlap with predefined critical regions, or requiring that a large fraction of the CNV lies within the target region [20, 26, 66, 85, 136, 231]. When deletions span multiple genes, they are typically assigned to each affected gene and included independently in gene-level analyses [18]. In such cases, additional prioritisation strategies may be applied, for example, by focusing on variants predicted to disrupt coding regions (pLoF events) or by stratifying analyses based on gene-level intolerance metrics, such as the probability of loss-of-function intolerance (pLI) or the loss-of-function observed/expected upper bound fraction (LOEUF), which can help identify biologically relevant signals and potential driver genes within multi-gene events [26, 86, 242]. Region-level analysis merges overlapping CNV calls into CNVRs and tests these merged regions for association with the phenotype [26, 27, 134, 136, 243]. In sequencing-based data, particularly GS, CNV grouping requires additional consideration because of the higher breakpoint resolution and variability across detection algorithms [5, 18]. Although breakpoints can be defined at near base pair resolution, different callers may produce slightly discordant or fragmented calls for the same event [244]. Consequently, clustering strategies, such as reciprocal overlap thresholds or distance-based merging, are commonly applied to harmonise CNVs across samples and tools while preserving breakpoint precision [244]. In tiling-window analysis, the genome is divided into fixed-size, potentially overlapping windows (e.g. 10 kb), and CNVs within each window are aggregated for unbiased genome-wide scans [76, 231]. At the CNV level, individual CNVs, common or rare, are tested separately, which is most appropriate for well-characterised variants with sufficient frequency and expected effect sizes [18, 20].

At the association testing stage, different analytical paradigms can be applied to grouped data. Single-variant analysis tests each probe, CNV, or region independently, whereas burden-style analysis aggregates multiple CNVs (e.g. all rare CNVs overlapping a gene) to evaluate their collective effect [18, 19, 21, 26, 27, 66, 76, 134, 139, 236–239, 245]. Standard regression methods, such as logistic regression for binary traits and linear regression for quantitative traits, are widely used [18, 19, 26, 27, 66]. In sequencing-based analyses, CNVs can be modelled using continuous dosage estimates derived from the read depth or allele balance, rather than binary presence/absence. This approach is analogous to SNP dosage models used in GWAS and enables more precise effect estimation compared to array-based approaches [66]. Fisher’s exact test and the chi-square test are commonly used to compare the frequency of a CNV between groups; in small samples or when expected cell counts are low, Fisher’s exact test is preferred because it calculates the exact probability [26, 134, 139, 231, 237, 239, 246]. Likelihood ratio tests (LRTs) can be used to assess the significance of CNV terms within regression models [136]. Other methods include kernel-based tests (e.g., SKAT, CKAT, CONCUR, MCKAT, SMCKAT) for rare-variant contexts with heterogeneous effects [158, 247–252], Cox proportional hazards models for time-to-event outcomes such as age at disease onset [26], mixed models (e.g., EMMAX [253], BOLT-LMM [254, 255]) to account for relatedness and population structure [20, 76], permutation-based tests for empirical significance estimation [134, 139, 239, 245], and family-based association tests in related cohorts [237, 256, 257]. Family-based association analyses, including family-based association tests (FBATs) and transmission disequilibrium-based approaches, are particularly important in CNV studies, as they leverage pedigree information (e.g. trios or multiplex families) to evaluate transmission patterns of variants [236, 237, 241]. Trio-based designs are particularly valuable for detecting de novo CNVs, assessing Mendelian consistency, and investigating their contribution to disease risk, especially in neurodevelopmental disorders such as autism spectrum disorder [5, 70, 78, 236, 237, 241]. The transmission disequilibrium test (TDT) evaluates whether alleles or CNVs are transmitted from heterozygous parents to affected offspring more frequently than expected by chance, providing a robust framework that is resistant to population stratification [258]. In addition, conditional logistic regression stratified by family enables within-family comparisons by treating relatives as matched sets, thereby controlling for shared genetic background and environmental factors [237, 259]. In addition, some studies have focused specifically on de novo CNVs and assessed their association using regression-based frameworks (e.g. logistic regression), particularly in trio-based designs [241]. These designs are inherently robust to population stratification and control-related biases, which can confound case–control analyses [237]. Furthermore, these approaches facilitate the assessment of variant segregation within families, helping to clarify the pathogenic relevance of CNVs and identify variants that may contribute to disease susceptibility [241].

In addition to autosomal CNVs, the analysis of sex chromosomes presents distinct methodological challenges owing to differences in baseline ploidy between sexes, which violate standard diploid assumptions and require sex-specific normalisation or modelling strategies (Additional file 3: Note S5 [21, 25, 27, 47, 49, 50, 52, 66, 86, 134, 138, 158, 197, 225, 238, 260–262]). Despite these complexities, accurate detection of sex chromosome CNVs remains important due to their established roles in developmental disorders and sex-specific disease risk [263].

Furthermore, to reduce confounding and improve the accuracy of effect estimation, CNV association analyses routinely adjust for relevant covariates, such as age, ancestry (commonly represented via principal components), genotyping array or batch, assessment centre, and CNV length [18–21, 26, 66, 136, 238]. The inclusion of these covariates helps to control for population stratification and technical artefacts. Importantly, CNV patterns and their phenotypic associations can differ substantially across ancestral populations due to variation in demographic history, LD structure, and reference-related biases [8, 78]. CNV frequencies are often population-specific, which can influence statistical power, effect size estimation, and the generalisability of association findings [19, 78]. In particular, reduced LD between CNVs and SNPs in certain populations limits tagging efficiency, whereas the reliance on reference genomes derived predominantly from European populations may introduce alignment bias and affect CNV detection accuracy [8, 27, 78]. These factors can result in population-specific signals and reduced replication across studies if not properly accounted for, highlighting the importance of ancestry-aware analytical strategies in CNV association analysis.

Given the large number of statistical tests performed in genome-wide CNV studies, appropriate multiple test corrections are essential to control false positives. Common approaches include the Bonferroni correction, false discovery rate (FDR) control (e.g. the Benjamini–Hochberg procedure), and permutation-based methods [18, 24, 26, 85, 134, 231, 236, 237, 239, 245]. In some cases, the “effective number of tests” approach is applied to set genome-wide significance thresholds, where the effective count is estimated from the correlation structure among tests arising from overlapping or co-occurring CNVRs rather than assuming full independence [19, 26].

In the effect modelling stage, the representation of CNV status within the statistical model is determined. Dosage-based models treat copy numbers as continuous variables. The basic linear or additive dosage model assumes a proportional change in effect with each unit copy number change [66]. Specific forms include the mirror-effect model, where deletions and duplications have equal magnitude but opposite effects, and the U-shape model, where both deletions and duplications affect the phenotype in the same direction [19, 26, 27]. Probabilistic dosage models incorporate uncertainty in CNV calls by weighting the effects according to the probability of each copy number state [19]. Alternatively, categorical models treat each copy number state as a discrete category (genotypic model) or collapse the states into broader groups (dominant/recessive coding) [76, 243, 264]. Finally, type-specific models analyse deletions and duplications separately, allowing for the identification of distinct biological effects [20, 26, 27].

#### Copy number variation association tools

Specialised tools are essential for CNV association studies because CNVs differ fundamentally from SNPs in terms of their biological nature, measurement properties, and analytical complexities. Unlike SNPs, which are typically biallelic, CNVs can encompass a wide range of integer copy numbers and may manifest as deletions or duplications spanning entire genomic regions, resulting in more than three possible genotypes and increased allelic diversity [71, 88, 265]. CNV measurement is inherently noisier than SNP genotyping because of technical variations in intensity signals and systematic biases between cases and controls, which, if unaddressed, can inflate false-positive rates [71, 87]. Defining and merging CNVRs across individuals adds further complexity, as CNVs vary considerably in size and breakpoint locations. Accurate boundary refinement and region merging require advanced algorithms to handle overlapping calls, fragmented CNVs, and complex rearrangements [49, 50, 52, 266]. Importantly, the choice of merging strategy can substantially influence downstream analyses. Overly permissive merging may combine distinct variants and inflate false-positive signals, whereas overly stringent criteria may fragment shared CNVs, reduce statistical power, and obscure true associations [50, 52]. Breakpoint heterogeneity across individuals further complicates this process and may dilute association signals if not appropriately modelled [52]. In addition, different merging strategies (e.g., reciprocal overlap, density-based trimming, or fragment approaches) can lead to markedly different CNVR definitions, affecting both the localisation and interpretability of association signals [50, 66, 88]. Moreover, CNV data require specialised QC pipelines and effect models tailored to CNV biology. Standard SNP-based GWAS tools lack native support for these complexities, often missing crucial QC steps and statistical models that account for CNV-specific uncertainties and the more complex, often non-linear relationships between copy number and phenotypes [46, 47, 49, 52]. Consequently, the unique challenges posed by CNV data in terms of calling, representation, uncertainty, and association analysis necessitate the development of dedicated tools specifically tailored to the complexities of CNV association studies. Although various methods have been devised for calling CNVs, there is a scarcity of strategies for CNV association analysis (Additional file 1: Table S3 [46, 47, 49, 50, 52, 66, 71, 87, 88, 122, 181, 261, 265–268] and Additional file 1: Figs. S2-S8). Dedicated tools have nonetheless been developed, ranging from early extensions of GWAS software (for example, PLINK) to integrated pipelines that couple detection and association (for example, Birdsuite, R-GADA, ParseCNV/2), likelihood-based statistical frameworks (for example, CNVtools, CNVassoc), CNVR-defining approaches (for example, CNVRuler and CNVRanger), more recent scalable solutions for next generation sequencing (NGS) data (for example, CNest), and emerging frameworks tailored to LR-seq that integrate discovery, genotyping, phasing, and imputation for population-scale CNV association. For details and examples of their applications, see Additional file 3: Note S6 [5, 21, 26, 46, 47, 49, 50, 52, 66, 70, 71, 77, 87, 88, 122, 125, 181, 230, 261, 265–297].

### Post-association analysis and interpretation

Post-association analysis and interpretation refine and validate CNV findings and place them in a broader biological context. Fine-mapping approaches, including stepwise conditional analyses or identification of the most strongly associated probe or exon within a locus, help pinpoint likely causal CNVs or sub-regions within that locus [20, 27, 66, 231]. Replication in independent cohorts and orthogonal experimental validation methods, such as qPCR, MLPA, or GS, are essential for establishing the robustness of these associations. Integration with SNP GWAS data allows the assessment of overlap, LD relationships, and the potential for CNVs to provide novel association signals beyond those captured by SNPs [20, 26, 27, 66, 76]. Functional annotation and pathway analyses identify enriched biological processes, pathways, or protein interaction networks among associated loci [26, 76, 134, 236–238], whereas clinical relevance can be evaluated by mapping CNVs to known genomic disorders, syndromes, or by assessing pleiotropic effects [20, 26, 70, 232]. Additional integrative analyses, such as colocalisation with expression or epigenetic QTLs and phenome-wide association studies (PheWAS), can provide deeper insights into the molecular mechanisms and phenotypic spectrum of CNV effects [18, 27, 240]. Finally, sharing results, particularly summary statistics, through public repositories such as the GWAS Catalogue facilitates reproducibility, secondary analyses, and translation into clinical or public health applications [18, 20, 21, 27, 66].

## Conclusion and future perspectives

CNVs are a major class of SVs that shape human genetic diversity and influence susceptibility to a wide range of diseases. Substantial progress in sequencing technologies and computational frameworks has greatly enhanced CNV detection; however, important challenges persist in translating CNV discoveries into reliable association studies. A central limitation is that no single CNV calling tool consistently outperforms the others across all experimental settings; thus, the combined use of multiple algorithms remains the most robust strategy for CNV detection. Beyond detection, rigorous QC, careful CNV grouping, and appropriate statistical frameworks for association testing and effect modelling are essential to ensure valid and interpretable results. Together, these considerations underscore the complexity of CNV analysis and highlight the need for continued methodological innovation to fully elucidate the contribution of CNVs to health and diseases.

The landscape of CNV analysis is anticipated to experience profound changes driven by advances in reference genome assemblies, integrative methodologies, benchmarking standards, and computational tools. The development of comprehensive reference genomes, particularly the telomere-to-telomere (T2T) CHM13 assembly, has provided a gap-free human genome sequence that corrects structural errors and adds approximately 200 Mbp of previously unresolved sequences, many of which are located in centromeric, acrocentric, and telomeric regions, and includes additional protein-coding genes [298]. Building on this, the creation of human reference pangenomes that represent diverse populations through genome graphs offers a powerful means to better characterise repetitive regions and complex SVs [299–302]. The Human Pangenome Reference Consortium has produced a draft comprising phased diploid assemblies with substantial euchromatic sequences and SVs that are absent from GRCh38 [303]. Tools such as GraphTyper [304, 305], which leverage pangenome graphs for population-scale genotyping of SV, exemplify how the integration of genome graph representations into large-scale studies can overcome reference bias, enhance read alignment, and facilitate accurate SV genotyping across diverse populations. However, transitioning existing annotations and benchmarks to graph-based references presents challenges, particularly in adapting gene definitions and regulatory annotations to this new framework [2].

In parallel, LR-seq has emerged as a transformative technology for SV discovery and CNV analysis. A population-scale LR-seq study by Beyter et al. [5] identified over 22,000 SVs per individual, three to five times more than those found using short-read sequencing, highlighting the substantial gains in sensitivity, especially for tandem repeat-associated SVs. Moreover, the integration of LR-seq-derived SVs with imputation into large cohorts has enabled functional insights, such as the identification of a rare deletion in *PCSK9* associated with reduced LDL cholesterol levels and a multiallelic repeat in *ACAN* linked to height [5]. More recently, Bai et al. [306] constructed a reference panel using 482 haplotype-resolved long-read assemblies. They developed an online imputation tool, ImputeSV, designed to impute SVs and tandem repeat variants from SNP data. This tool was applied to impute 54,578 common SVs in 456,643 participants from the UK Biobank. The study identified 17,335 SV-trait associations across 2,624 traits and estimated that SVs contributed to at least 4.7% of the common genetic variance associated with complex traits. These findings underscore the importance of LR-seq as a critical complement to pangenome approaches, offering comprehensive SV catalogues that better capture population diversity and disease-relevant variations.

Future directions also emphasise the integration of CNV and SNP association studies within a unified analytical framework. Many CNVs are poorly tagged by SNPs or occur on distinct haplotypes, underscoring the need for CNV-specific analysis. Approaches such as CNest have facilitated the classification of CNV associations based on their overlap with SNP signals, distinguishing between CNV-only, CNV-allele, SNP-CNV near, and SNP-CNV far associations. Joint modelling of SNP and CNV associations is anticipated to reveal shared genetic mechanisms, enhance the prioritisation of causal genes, and refine polygenic risk score (PGS) predictions, contingent upon the establishment of LD maps between SNPs and CNVs [27, 66, 307].

Future CNV research will increasingly adopt multimodal integration strategies that combine CNV data with other molecular layers, such as transcriptomic, epigenomic, and chromatin topology, to better understand the functional impact of SVs/CNVs. Recent studies have highlighted how rare germline SVs can disrupt highly expressed and mutationally constrained genes, alter regulatory domains such as topologically associating domains (TADs), and dysregulate gene expression in tissue-specific contexts [3, 28]. For example, the integration of CNV data with RNA sequencing and epigenetic profiles from disease-relevant tissues has demonstrated that singleton gene-disruptive germline CNVs preferentially impact genes expressed in the tissue of origin, potentially leading to downstream transcriptional dysregulation observable in paediatric tumours. Furthermore, non-coding CNVs that overlap with tissue-specific chromatin boundaries are associated with disruptions in three-dimensional genome architecture, linking CNVs to alterations in the regulatory networks [308].

Benchmarking and evaluation of CNV detection remain challenging, particularly in repetitive genomic regions. Emerging tools, such as TT-Mars, which compare SV calls to haplotype-resolved assemblies, are helping to address benchmarking challenges in repetitive regions [214]. Additionally, the nf-core/variantbenchmarking pipeline streamlines evaluation by integrating diverse truth sets and supporting comparisons across assemblies, including CNV-specific benchmarks [309].

Beyond benchmarking, there is a critical need to standardise reporting guidelines and metadata definitions to ensure reproducibility and interoperability, ultimately facilitating the inclusion of CNV findings in public resources, such as the GWAS Catalogue. In this context, causal inference methods, such as transcriptome-wide Mendelian randomisation, are particularly valuable, as they provide robust frameworks for linking CNVs to functional gene expression changes and phenotypic effects, thereby strengthening the evidence required for CNV association results to be formally integrated into catalogues and downstream analyses, such as PGS and drug target prioritisation [27, 82, 310].

Innovative workflows and haplotype-aware methods have transformed the large-scale discovery and analysis of CNVs. For example, the CNest workflow enables high-resolution CNV detection from NGS read depth across very large cohorts and has already uncovered hundreds of novel associations in the UK Biobank [66]. Crucially, CNest adheres to the Global Alliance for Genomics and Health (GA4GH) standards, ensuring interoperability and standardised data exchange in population-scale studies [311]. Complementing this, haplotype-informed approaches, such as HI-CNV, substantially boost detection sensitivity by leveraging shared haplotypes in biobank-scale datasets, identifying more than six times as many CNVs per individual as earlier methods [20]. Finally, advances in statistical phasing tools, such as SHAPEIT5, provide the haplotype scaffolding necessary for CNV analyses, enabling the exploration of phase-dependent allelic series and their downstream phenotypic consequences [312].

## Supplementary Information

Additional file 1: Figs. S1-S8 and Tables S2-S3. Document containing eight figures and two tables. Fig. S1 shows trends in CNV association studies over time. Figs. S2-S7 present citation landscapes for array-based, exome sequencing-based, genome sequencing-based, targeted panel-based, long-read sequencing-based, and ensemble-based CNV detection tools, respectively, while Fig. S8 presents the citation landscape for CNV association tools. Table S2 summarises common CNV callers, including their data inputs, calling approach, and associated repositories. Table S3 summarises common CNV association tools, including their input data types, study designs, statistical methods, strengths, and limitations.

Additional file 2: Table S1. An Excel spreadsheet listing 131 published copy number variation association studies, including authors, publication year, study title, sequencing/array platform used, journal, and citation count.

Additional file 3: Notes S1-S6. The document provides a detailed background on CNV detection platforms and methodologies (Note S1), an overview of detection strategies across array- and sequencing-based platforms (Note S2), segmentation algorithms used in CNV calling (Note S3), platform-specific benchmarking comparisons (Note S4), challenges in sex chromosome CNV analysis (Note S5), and a tool-by-tool review of CNV association software (Note S6).

## Data Availability

This review did not include newly generated primary data. Fig. 2 was produced using publicly available data from Collins et al. [14, 313], specifically the annotated list of disease-associated CNV loci provided in Table S3 of that study. Additional file 1: Fig. S1 was generated using a published compilation of CNV GWAS studies provided in Supplementary Information 2 of Harris et al. [8, 314] and supplemented with additional studies curated by the authors. The combined list of CNV association studies is shown in Additional file 1: Fig. S1, with detailed study characteristics provided in Additional file 2: Table S1.
