# Supplementary material for: Computational strategies for copy number variation detection, disease association, and beyond
Source: Genome Biol. 2026 Jul 10;27:287. doi: 10.1186/s13059-026-04189-6 (PMC13551817; doi:10.1186/s13059-026-04189-6)
Supplement: Supplementary file 1 [file 13059_2026_4189_MOESM1_ESM.docx]

**Fig. S1.** Trends in CNV association studies over time. Bump plot illustrating the number of published studies applying CNV modelling for trait association testing from 2008 to 2024, stratified by technology. Most studies have relied on SNP microarrays (orange), whereas more recent studies have increasingly used short-read sequencing (green) and long-read sequencing (blue), which provide higher resolution. The data are summarised in Additional file 2: Table S1. The figure was generated using RAWGraphs.

**Fig. S2.** Citation landscape of array-based copy number variation (CNV) detection and association tools. This visualisation was generated using Litmaps (1) and provides a citation-based overview of the tools for CNV detection and association. Each node represents a publication describing a CNV-related tool in the network. Edges denote the citation relationships between publications. The horizontal axis represents the publication date (more recent articles are positioned to the right), while the vertical axis reflects citation momentum (citation count adjusted for recency), highlighting influential recent studies. The node size corresponds to the total citation count. Node colours indicate sequencing platforms or methodological categories, and multicoloured nodes represent tools that are applicable across multiple settings. This analysis includes peer-reviewed publications available up to March 2026. Tools lacking standalone publications (e.g. those described only in company documentation or broader platform resources rather than in dedicated research articles) were excluded, which may have influenced representation. In addition, some tools are introduced as part of broader project papers rather than dedicated tool-specific manuscripts, which may affect their citations.

**Fig. S3.** Citation landscape of exome sequencing (ES) CNV detection tools. See Fig. S2 for a detailed description of the visualisation framework. This panel focuses on ES-based CNV detection tools.

**Fig. S4.** Citation landscape of genome sequencing (GS) CNV detection tools. See Fig. S2 for detailed description of the visualisation framework. This panel focuses on GS-based CNV detection tools.

**Fig. S5.** Citation landscape of targeted panel approaches. See Fig. S2 for a detailed description of the visualisation framework. This panel focuses on targeted panel-based CNV detection tools.

**Fig. S6.** Citation landscape of long-read sequencing callers developed for PacBio and ONT technologies. See Fig. S2 for a detailed description of the visualisation framework. This panel focuses on long-read sequencing-based CNV detection tools.

**Fig. S7.** Citation landscape of ensemble tools that implement integrative approaches. See Fig. S2 for a detailed description of the visualisation framework. This panel focuses on ensemble-based CNV detection tools.

**Fig. S8.** Citation landscape of CNV association tools designed to evaluate the associations between CNVs and phenotypic or disease traits. See Fig. S2 for a detailed description of the visualisation framework. This panel focuses on CNV association tools.

| Table S2. Common copy number variation callers | | | | | | |
| --- | --- | --- | --- | --- | --- | --- |
| Platform / Approach | Tool | Subtype(s) | Input(s) | Methodology | URL(s) | Reference(s) |
| Microarrays | ADM-2 | aCGH | Log_2_ ratios (Agilent output files) | Aberration Detection Method 2 – a statistical segmentation algorithm used in Agilent’s software | https://www.agilent.com/en/product/cgh-cgh-snp-microarray-platform | Agilent Technologies. CytoGenomics Software User Guide [software manual] |
|  | DNAcopy | aCGH | Log_2_ ratios | Uses CBS to detect change-points in log2 ratio signal | https://bioconductor.org/packages/release/bioc/html/DNAcopy.html | (2) |
|  | LRS | aCGH | Log_2_ ratios | The LRS algorithm (likelihood ratio selection) uses likelihood ratio tests to optimally identify CNV segments | https://www.tandfonline.com/doi/abs/10.1198/jasa.2010.tm10083 | (3) |
|  | Nexus | aCGH | Log_2_ ratios | A commercial toolkit integrating multiple algorithms (CBS, FASST2, etc.) to call CNVs and visualise results (proprietary methods) | https://bionano.com/nexus-copy-number-software/ | (4) |
|  | SaRa | aCGH | Log_2_ ratios | Screening and ranking algorithm for multiple change-point detection – identifies CNV breakpoints in O(n) time by iteratively detecting shifts in intensity | https://ysph.yale.edu/c2s2/software/sara/ | (5) |
|  | VEGA | aCGH | Log_2_ ratios | Variational Bayesian segmentation: uses an energy-minimisation model to find breakpoints more efficiently than CBS, achieving high accuracy | https://bioconductor.statistik.tu-dortmund.de/packages/2.9/bioc/html/Vega.html | (6) |
|  | iPattern | SNP-array | Allele-specific intensities for each SNP | Joint multi-sample analysis: computes total SNP intensity per sample, normalises across individuals, then uses a sliding-window Gaussian mixture model to call CNVs present in multiple samples | https://www.nature.com/articles/nature09146 | (7) |
|  | PennCNV | SNP-array | LRR and BAF values per SNP | HMM using both LRR and BAF to infer CN states | https://penncnv.openbioinformatics.org/en/latest/ | (8) |
|  | QuantiSNP | SNP-array | LRR and BAF values per SNP | HMM-based like PennCNV, modelling LRR and BAF jointly | https://github.com/cwcyau/quantisnp | (9) |
|  | R-GADA | SNP-array | Normalised Log_2_ ratios | The Genome Alteration Detection Algorithm (GADA), implemented in R, uses sparse Bayesian learning to segment the intensity signal. R-GADA pipeline includes data normalisation and logistic regression for post-processing | https://github.com/isglobal-brge/R-GADA | (10) |
| SR-seq | CANOES | ES | Per-target coverage matrix | Negative-binomial model: models read counts per target with a negative binomial and estimates variance via regression on a reference set | https://github.com/ShenLab/CANOES | (11) |
|  | CLAMMS | ES | BAM, per-target coverage matrix | Lattice-aligned mixture models: fits a mixture model for each exon across tens to thousands of samples | https://github.com/rgcgithub/clamms | (12) |
|  | CONIFER | ES | Per-exon RPKM values | Detects CNVs from exome data by normalising read counts (RPKM), removing technical and batch biases using SVD, and then identifying CNVs with a threshold on the cleaned signal | https://conifer.sourceforge.net/ | (13) |
|  | CONTRA | ES | BAM, SAM | Computes log_2_ read depth ratio per exon, then applies statistical thresholds to call CN gains/losses | https://contra-cnv.sourceforge.net/ | (14) |
|  | EXCAVATOR/2 | ES | BAM | An ES-focused CNV tool that normalises read counts for GC content, mappability, and exon length, and then computes log_2_ ratios between test and control exomes. It segments the normalised ratio using HSLM (an SLM variant accounting for gaps between exons) to find CNV breakpoints and classifies each segment into five CN states (homozygous deletion, hemizygous deletion, normal, gain, and amplification) using the FastCall algorithm. EXCAVATOR2 is an enhanced version of EXCAVATOR that achieves genome-wide CNV resolution by exploiting both on-target and off-target reads from ES experiments, alongside improved normalisation | https://sourceforge.net/projects/excavatortool/  https://sourceforge.net/projects/excavator2tool/ | (15, 16) |
|  | ExCopyDepth | ES | Per-exon coverage matrix | ExCopyDepth combines ExomeCopy and ExomeDepth to enhance CNV detection from ES data. It uses ExomeCopy for exon-length normalisation and extraction of read count and GC content, and then leverages ExomeDepth to select the optimal reference set and call CNVs. This hybrid approach minimises GC bias and improves reference set selection, leading to more stringent and accurate CNV calls | In-house modification to ExomeCopy and ExomeDepth | (17) |
|  | ExomeCNV | ES | BAM | Read depth + BAF in exome: combines the per-exon depth ratio and SNP BAF to detect CN changes. Uses CBS segmentation on depth and evaluates BAF shifts to call CNVs | https://github.com/cran/ExomeCNV | (18) |
|  | exomeCopy | ES | BAM | HMM with covariates: fits an HMM to the read count sequence, modelling two hidden states (normal vs. CNV). It incorporates covariates, such as GC content, to adjust the expected read counts. Returns the Viterbi path of CN states for each exon | https://www.bioconductor.org/packages//2.10/bioc/html/exomeCopy.html | (19) |
|  | ExomeDepth | ES | BAM | Beta-binomial per-exon model: builds an optimised reference by combining the read counts of multiple samples. For each exon, it models the test sample’s count as beta-binomial distributed around the expected count (derived from reference set) | https://github.com/vplagnol/ExomeDepth | (20) |
|  | HMZDelFinder | ES | BAM | Homozygous deletion finder: identifies exons with near-zero coverage in a proband while reads are present in others | https://github.com/BCM-Lupskilab/HMZDelFinder | (21) |
|  | PatternCNV | ES | BAM | Utilises a pattern-recognition approach for detecting CNVs specifically at the exon level | https://github.com/nordhuang/patternCNV | (22) |
|  | XHMM | ES | Per-target coverage matrix | PCA normalisation + HMM: PCA is performed on the sample-by-target read depth matrix to remove the top PCs (systematic noise). Then applies an HMM to the normalised read depth Z-scores to call deletions/duplications | https://github.com/RRafiee/XHMM | (23) |
|  | Canvas | ES, GS | BAM | Read depth and MAF-based CNV caller: bins reads into variable-size windows (k-mer-based), applies GC correction, and then segments normalised coverage using an unbalanced Haar wavelet transform (default) or CBS. CN states are assigned jointly from coverage and MAF at heterozygous SNV sites using a Gaussian mixture model. Supports SmallPedigree-WGS workflow | https://github.com/Illumina/canvas | (24) |
|  | cn.MOPs | ES, GS | Read count matrix, BAM | Mixture of Poissons model: models read counts per genomic position across multiple samples using a mixture of Poisson distributions to distinguish CN states | https://www.bioinf.jku.at/software/cnmops/cnmops.html | (25) |
|  | CNest | ES, GS | BAM, CRAM | CNest extracts read coverage from sequencing data and uses sample sex classification for QC. Dynamic reference samples were then selected to normalise technical variations and convert coverage data into median-normalised log_2_ ratios. Using a custom three-state HMM, CNest calls CNVs | https://github.com/tf2/CNest | (26) |
|  | FREEC | ES, GS | BAM, SAM | Read depth + BAF + GC-correction: performs GC content normalisation and optionally uses matched normal or population GC profiles. Segments read depth and incorporates BAF from SNPs to refine zygosity of calls | https://boevalab.inf.ethz.ch/FREEC/ | (27) |
|  | Manta | ES, GS | BAM | Hybrid SV calling with local assembly: identifies SV candidates from discordant read pairs and split reads, then performs local *de novo* assembly around each candidate to refine breakpoints | https://github.com/Illumina/manta | (28) |
|  | ClinCNV | ES, GS, targeted panel | BED, Per-target coverage matrix | Multi-sample joint calling: performs joint normalisation across many samples and uses a custom algorithm combining elements of CBS segmentation and HMM modelling | https://github.com/imgag/ClinCNV | (29) |
|  | DRAGEN | ES, GS, targeted panel | FASTQ, BAM, CRAM | Hardware-accelerated pipeline: DRAGEN’s CNV/SV caller combines discordant pair and split read signals with read depth changes into an integrated calling strategy | https://help.dragen.illumina.com/dragen-v4.5/product-guides/dragen-v4.5/dragen-dna-pipeline/cnv-overview/cnv-germline | (30) |
|  | GATK-gCNV | ES, GS, targeted panel | BAM | GATK-gCNV detects CNVs by modelling the read depth using a Bayesian probabilistic approach. It first generates a read-count matrix from aligned sequence data and then applies cohort-based normalisation to remove technical variations (e.g. GC bias and capture efficiency). Using a HMM, it infers the most likely CN state at each interval for each sample | https://github.com/broadinstitute/GATK-gCNV-publication | (31) |
|  | CNVkit | ES, targeted panel | BAM | Toolkit for depth normalisation + segmentation: combines on-target read depths with off-target (off-panel) reads to increase the signal-to-noise ratio. Performs GC and bias corrections, then segments log_2_ coverage ratios using CBS by default | https://cnvkit.readthedocs.io/en/stable/ | (32) |
|  | CODEX/2 | ES, targeted panel | Per-exon coverage matrix | Latent factor normalisation + CBS: uses a Poisson latent factor model to remove biases due to GC content, exon length, and capture efficiency. After normalisation, CBS segmentation is applied to call CNVs. CODEX2 improves upon CODEX by preserving common CNV signals | https://github.com/yuchaojiang/CODEX2 | (33, 34) |
|  | DECoN | ES, targeted panel | BAM | DECoN detects exon CNVs by comparing the read depth of each exon in a test sample to a reference set following stringent QC and provides both tabular and interactive visual output. It is based on the statistical framework of ExomeDepth and is tailored for high-quality clinical diagnostics | https://github.com/RahmanTeam/DECoN | (35) |
|  | BIC-seq2 | GS | BAM | Read depth segmentation with BIC: normalises GC/mappability at nucleotide level, then applies a BIC-based algorithm to segment the genome and detect CNVs | https://github.com/ding-lab/BICSEQ2?tab=readme-ov-file | (36) |
|  | BreakDancer | GS | BAM | Discordant paired-end mapping: clusters read pairs with abnormal insert sizes or orientation to detect deletions, insertions, inversions, and inter-chromosomal translocations | https://github.com/genome/breakdancer | (37) |
|  | BreakSeq/2 | GS | BAM, SAM | Breakpoint-library alignment: aligns reads to a library of known non-reference breakpoint junction sequences. Detects SVs by matching soft-clipped read sequences to known SV junctions, achieving nucleotide-resolution calls | https://bioinform.github.io/breakseq2/ | (38, 39) |
|  | CNVnator | GS | BAM | Binned read depth analysis: computes read depth in fixed-size bins, performs GC correction, then uses a mean-shift clustering algorithm to identify significant coverage shifts indicating CNVs | https://github.com/abyzovlab/CNVnator | (40) |
|  | Cue | GS | BAM, CRAM | Deep learning SV caller: converts alignment data into image-like representations encoding coverage, insert size, and allele distributions, then applies a convolutional neural network (hourglass CNN) to detect SVs | https://github.com/PopicLab/cue | (41) |
|  | Delly | GS | BAM | An SV/CNV caller that leverages multiple signals: discordant paired-end reads, split read alignments, and read depth coverage. It also supports long-reads from PacBio or ONT | https://github.com/dellytools/delly | (42) |
|  | ERDS | GS | BAM + SNP calls (VCF) | Read depth + SNV integration: uses a paired HMM that combines the depth of coverage with SNP zygosity shifts. Also leverages discordant read pair signals | https://github.com/igm-team/ERDS | (43) |
|  | forestSV | GS | BAM | A machine learning approach for SV/CNV discovery that integrates prior knowledge of SV features. ForestSV extracts a wide array of sequence features from alignment data and trains a random forest model on known variants. Given a new BAM, it constructs feature vectors and predicts deletions or duplications by classification, outputting high-confidence CNV calls with associated scores | https://sebatlab.org/data-software | (44) |
|  | GRIDSS | GS | BAM | GRIDSS identifies variants through a genome-wide break-end assembly using an alignment-guided positional de Bruijn graph, along with evidence from split reads and read pairs | https://github.com/PapenfussLab/gridss | (45) |
|  | iCopyDAV | GS | BAM | Integrated CNV detection, annotation, and visualisation: a pipeline with modules for bias correction, segmentation, variant calling, and visualisation. It performs read depth segmentation (CBS or similar) on coverage tracks and provides an interactive interface to review/edit calls | https://bioinf.iiit.ac.in/icopydav/ | (46) |
|  | JointSLM | GS | Normalised depth, produced by RDXplorer | Joint sparse learning model: a population-based segmentation that assumes common CNVs across individuals. Uses a statistical model across multiple samples’ read depth to find shared CNV segments | https://academic.oup.com/nar/article/39/10/e65/1309398 | (47) |
|  | LUMPY | GS | BAM | Probabilistic multi-signal integration: combines evidence from discordant pairs and split reads in a unified model | https://github.com/arq5x/lumpy-sv | (48) |
|  | Pindel | GS | BAM | Split read pattern growth: aligns unaligned read segments to find sequence patterns that span breakpoints | https://github.com/genome/pindel | (49) |
|  | RDXplorer | GS | BAM | Event-wise statistical testing: normalises read depth per window, then uses a sliding window approach with hypothesis testing on each region to detect significant depth deviations | https://rdxplorer.sourceforge.net/ | (50) |
|  | ReadDepth | GS | BED | Segmentation using CBS and statistical testing for significant CN changes | https://github.com/chrisamiller/readDepth | (51) |
|  | SoftSV | GS | BAM | Paired-end + soft-clip exact calling: processes soft-clipped and discordant alignments to precisely identify breakpoints for deletions, inversions, tandem duplications, and translocations. Uses a robust algorithm to integrate split read alignments and paired-read evidence, yielding single-nucleotide resolution SV breakpoints | https://sourceforge.net/projects/softsv/ | (52) |
|  | SVseq2 | GS | BAM | A targeted SV/CNV caller designed for low-coverage genomes, which finds exact breakpoints of deletions and insertions by an improved split read strategy | https://sourceforge.net/projects/svseq2/ | (53) |
|  | TIDDIT | GS | BAM | Combined evidence approach: clusters discordant paired/split reads and contigs (local assembly) using DBSCAN for SV calls (deletions, duplications, inversions, translocations) | https://github.com/SciLifeLab/TIDDIT | (54) |
|  | Wham | GS | BAM | Uses graph-based integration of multiple alignment signals (split reads, discordant pairs, soft clips) and a random forest classifier to detect and label CNVs | https://github.com/zeeev/wham | (55) |
|  | Atlas-CNV | Targeted panel | Per-exon coverage matrix | Depth-of-coverage approach for panels: normalises coverage within each sample, computes log_2_ (sample/median) for each exon, and identifies CNVs when this ratio exceeds the thresholds. Applies Z-score based filters (C-score) to reduce false positives, enabling single-exon CNV detection | https://github.com/theodorc/Atlas-CNV | (56) |
|  | CoNVaDING | Targeted panel | BAM | Control-based Z-score method: for each target, selects a set of reference samples with similar coverage behaviour and computes a Z-score for the test sample’s depth | https://molgenis.gitbook.io/convading/ | (57) |
|  | ifCNV | Targeted panel | BAM | Uses an isolation forest machine learning algorithm to flag coverage outliers (possible CNVs) and then scored these to call genuine CNV events in panel data, without needing external controls | https://github.com/SimCab-CHU/ifCNV | (58) |
|  | panelcn.MOPS | Targeted panel | BAM | Adaptation of cn.MOPS for panels: performs QC on targets, then applies the cn.MOPS Poisson mixture model across multiple panel samples | https://www.bioinf.jku.at/software/panelcnmops/index.html | (59) |
|  | SOPHiA GENETICS DDM | Targeted panel | FASTQ | Proprietary algorithm: SOPHiA GENETICS’ DDM platform uses the MUSKAT™ algorithm for CNVs, an approach that performs double normalisation on panel read counts | https://www.sophiagenetics.com/sophia-ddm-for-genomics/ | SOPHiA GENETICS. DDM Platform documentation [software manual] |
| LR-seq | cuteSV | ONT, PacBio | BAM | Signature clustering: extracts SV signatures from long-read alignments (e.g. large deletions in CIGAR and split read alignments) and applies a clustering-and-refinement strategy to call SVs | https://github.com/tjiangHIT/cuteSV | (60) |
|  | Delly | ONT, PacBio | BAM | An SV/CNV caller that leverages multiple signals: discordant paired-end reads, split read alignments, and read depth coverage. It also supports short-reads from GS | https://github.com/dellytools/delly | (42) |
|  | NanoSV | ONT, PacBio | BAM | Split read clustering: identifies split or gapped alignments in long-read mappings and clusters them by orientation and genomic position to define SV breakpoint junctions | https://github.com/mroosmalen/nanosv | (61) |
|  | NanoVar | ONT, PacBio | FASTA, FASTQ, BAM, CRAM | Aligns long-reads and uses a neural-network classifier to detect SVs at low coverage | https://github.com/benoukraflab/NanoVar | (62) |
|  | Picky | ONT, PacBio | FASTQ | Uses the LAST aligner to break each long-read into high-scoring segments, then “stitches” these segments with a greedy seed-and-extend algorithm to assemble SV sequences | https://github.com/TheJacksonLaboratory/Picky | (63) |
|  | Sniffles/2 | ONT, PacBio | BAM, CRAM | Multi-signal read-based calling: parses long-read alignments to find SV evidence from split reads, large gaps/mismatches and coverage shifts. Sniffles clusters split read breakpoints, leverages large soft-clipped sequences and supplementary alignments as insertion signals, and checks read depth anomalies to call SVs. Sniffles2 builds on Sniffles by adding repeat-aware clustering (handles reads in repetitive regions more carefully) and a fast consensus assembly for insertion sequences | https://github.com/fritzsedlazeck/Sniffles | (64, 65) |
|  | SVIM-asm | ONT, PacBio | BAM | Assembly-to-reference SV detection: aligns a genome assembly (haploid or diploid) to a reference and identifies SVs from alignment differences | https://github.com/eldariont/svim-asm | (66) |
|  | SyRI | ONT, PacBio | Whole-genome alignment of two assemblies | Assembly-vs-assembly differencer: synteny and rearrangement identifier that compares two whole-genome assemblies to find both large-scale rearrangements and local variations | https://zenodo.org/records/3555197 | (67) |
|  | PBHoney | PacBio | BAM, SAM | Hybrid strategy: contains two algorithms – PBHoney-Spots, which scans each aligned PacBio read for regions with abnormally high discordance (error/mismatch density) to flag potential SV loci, and PBHoney-Tails, which captures reads with large soft-clipped ends (>200 bp), re-aligns those “tails” to the genome, and clusters them to pinpoint SV breakpoints | https://sourceforge.net/projects/pb-jelly/ | (68) |
|  | pbsv | PacBio | BAM | PacBio consensus-based caller: identifies candidate SV breakpoints from PacBio read alignments by identifying clusters of anomalous alignment signatures (e.g. split reads or large gaps). For each candidate, pbsv assembles a consensus sequence from the supporting reads (using partial-order alignment) and re-aligns this consensus to precisely determine the REF/ALT sequences and breakpoints | https://github.com/PacificBiosciences/pbsv | Pacific Biosciences. pbsv [software]. https://github.com/PacificBiosciences/pbsv |
| Ensemble/Merging Approaches | EnsembleCNV | Microarrays (SNP-array) | Final Report files (LRR and BAF) | Ensemble learning framework: merges calls from PennCNV, QuantiSNP, and iPattern, and then re-genotypes the aggregated CNV loci using a local likelihood model. Provides CNV genotypes with confidence scores | https://github.com/HaoKeLab/ensembleCNV | (69) |
|  | CN-Learn | SR-seq (ES) | BAM | Random-forest ensemble for exomes: runs four ES CNV callers (e.g., CANOES, CODEX, CLAMMS, XHMM) and uses a trained random forest classifier to combine their outputs | https://github.com/girirajanlab/CN_Learn | (70) |
|  | SVMerge | SR-seq (GS) | BAM | Pipeline integration framework: runs a suite of SV callers (e.g., BreakDancer, Pindel) and then filters and merges their results | https://svmerge.sourceforge.net/ | (71) |
|  | Parliament2 | SR-seq (GS) | BAM | Integrated pipeline: runs multiple best-in-class SV callers in parallel (e.g., BreakDancer, BreakSeq2, CNVnator, Delly, Manta, and LUMPY) and then merges their outputs into a consensus SV set | https://github.com/fritzsedlazeck/parliament2 | (72) |
|  | MetaSV | SR-seq (GS) | BAM | Meta-caller ensemble: merges SV calls from multiple algorithms (e.g., BreakDancer, Pindel, and CNVnator) to produce a consolidated, high-confidence callset. Improves SV resolution by leveraging orthogonal SV signals (e.g., depth, split, and pair evidence) from different callers. Can optionally perform local assembly for unresolved calls | https://bioinform.github.io/metasv/ | (73) |
|  | HugeSeq | SR-seq (GS) | FASTQ, BAM | HugeSeq runs a suite of ~24 tools in parallel: for CNVs, it combines read pair methods (e.g. BreakDancer), split read methods (Pindel), read depth callers (CNVnator), and known pattern matching (BreakSeq). The pipeline automates alignment, QC, variant calling, and annotation, finally merging results into consolidated VCF outputs | https://github.com/StanfordBioinformatics/HugeSeq | (74) |
|  | FusorSV | SR-seq (GS) | VCF | Uses a data-mining classifier to weight and fuse contributions from individual callers based on their performance characteristics | https://github.com/timothyjamesbecker/FusorSV | (75) |
|  | combiSV | LR-seq (ONT, PacBio) | VCF | Ensemble merging: combines SV calls from multiple long-read callers into a superior consensus call set. It takes VCF outputs from tools like Sniffles, pbsv, SVIM, cuteSV, NanoVar, and NanoSV, merging overlapping calls to boost recall and precision | https://github.com/ndierckx/combiSV | (76) |
|  | NextSV | LR-seq (ONT, PacBio) | FASTQ | Meta-caller pipeline: automatically runs multiple aligners and SV callers and integrates their results. For example, it can map reads with minimap2, NGMLR, and winnowmap, call SVs with Sniffles and PBHoney, then merge outputs | https://github.com/Nextomics/nextsv | (77) |
|  | SURVIVOR | Any | VCF | Merges SV call sets within or across samples, given a maximum distance and type matching criteria | https://github.com/fritzsedlazeck/SURVIVOR | (78) |
| Abbreviations: aCGH = array comparative genomic hybridisation; BAM = Binary Alignment Map; BAF = B-allele frequency; BED = Browser Extensible Data; BIC = Bayesian Information Criterion; bp = base pair; CBS = circular binary segmentation; CN = copy number; CNV = copy number variation; CRAM = Compressed Reference-oriented Alignment Map; CNN = convolutional neural network; DBSCAN = density-based spatial clustering of applications with noise; ES = exome sequencing; GC = Guanine–Cytosine content; GS = genome sequencing; HMM = hidden Markov model; HSLM = heterogeneous shifting level model; LRR = log R ratio; LR-seq = long-read sequencing; MAF = minor allele frequencies; ONT = Oxford Nanopore Technologies; PacBio = Pacific Biosciences; PCA = principal component analysis; QC = quality control; REF/ALT = reference/alternate alleles; RPKM = reads per kilobase per million mapped reads; SAM = Sequence Alignment Map; SNV = single nucleotide variant; SNP = single nucleotide polymorphism; SNV = single nucleotide variant; SR-seq = short-read sequencing; SV = structural variant; SVD = singular value decomposition; VCF = Variant Call Format | | | | | | |

| Table S3. Common copy number variation association tools | | | | | | | | | | | | |
| --- | --- | --- | --- | --- | --- | --- | --- | --- | --- | --- | --- | --- |
| Tool | Input | Study Design | Phenotype | CNV Type | Uncertainty, QC, Bias Handling | User Interface | Association Method | Strengths | Limitations | CNV Calling | URL(s) | Reference(s) |
| Birdsuite | SNP array intensities (Affy SNP 6.0+) | GWAS (large cohorts, families possible) | Binary; quantitative (via external PLINK) | Common; rare CNVs | HMM noise modelling; probe-level QC; length/frequency filters | CLI | Association via external tools | Simultaneous SNP genotyping and CNV calling on Affy 6.0 | Affy 6.0 only; discontinued; external analysis required | Yes | https://github.com/ohsu-comp-bio/birdsuite | (79) |
| CNest | BAM | Ultra-large GWAS (e.g. 200k UKB) | Binary; quantitative | Common; rare CNVs | Rigorous normalisation; bias correction | CLI (Docker, Nextflow) | Regression | Large-scale, robust CNV GWAS | Additive model only; requires large cohort sizes for stable normalisation; computationally intensive | Yes | https://github.com/tf2/CNest | (26) |
| CNVassoc | CNV genotype probabilities (arrays, MLPA) | Population case-control; quantitative | Binary; quantitative; survival; count outcomes | Mainly common CNVs | Explicit uncertainty modelling via latent class model | R | GLM framework with copy number uncertainty | Flexible outcomes; rigorous uncertainty modelling | Not CNV discovery; common CNVs only | Yes (basic) | https://github.com/isglobal-brge/CNVassoc | (80) |
| CNVRanger | CNV segments (bed-like) | Case-control; quantitative (CNV-eQTL) | Binary; quantitative; expression | Any CNV | Assumes quality calls; checks biases | R/Bioconductor | edgeR GLMs (expression); external PLINK regression | Comprehensive CNV toolkit, supports probe-level GWAS for SNP-chip CNVs | Not a CNV caller; external analysis required | No | https://waldronlab.io/CNVRanger/ | (81) |
| CNVRuler | Pre-called CNVs (arrays) | Case-control; quantitative | Binary; quantitative | Any CNV | No internal modelling; population stratification adjustment | Java GUI/R backend | Fisher’s exact test; chi-square; regression | User-friendly GUI; flexible CNVR definitions | Outdated; GUI limits automation; no uncertainty handling | No | http://www.ircgp.com/CNVRuler/index.html | (82) |
| CNVtest | SNP array intensity data; can use NGS read depth | Case-control; quantitative | Binary; continuous | Common; rare CNVs | Directly models intensities; LRS noise control | R | GLM | No predefined CNVs needed | Complex algorithm; limited documentation | Yes (implicit) | https://github.com/wuqian77/CNVtest | (83) |
| CNVtools | Quantitative intensity data (locus-specific) | Case-control; quantitative | Binary; continuous | Mainly multi-allelic; common CNVs | EM-based mixture model; explicit uncertainty modelling | R | GLM; likelihood ratio tests (mixture models) | Robust uncertainty modelling | Designed for locus-by-locus analysis; computationally complex | Yes (implicit) | http://bioconductor.jp/packages/3.0/bioc/html/CNVtools.html | (84) |
| CoNVaQ | Pre-called CNV segments (≥2 groups) | Case-control; categorical groups | Binary (group labels only) | Recurrent CNVs | Permutation-based significance; no uncertainty modelling | Web interface (website unavailable at time of writing); R | Fisher’s exact test with user-defined query CNV region overlap model | User-friendly | Binary analysis only; no covariates | No | https://github.com/SimonLarsen/convaq | (85) |
| ParseCNV/2 | CNV call lists; VCF (arrays, NGS) | Case-control; family; quantitative | Binary; quantitative; family data | Rare; common CNVs (filters common by default) | Integrated QC (call and region-level) | CLI (PLINK2 integration) | Fisher’s exact test; regression; rare-variant tests, family tests (RvTests integration) | Versatile statistical options; extensive QC | Complex CLI; no built-in CNV discovery | No | https://parsecnv.sourceforge.net/  https://github.com/CAG-CNV/ParseCNV2 | (86, 87) |
| PeakCNV | Case-control CNV segments | Case-control only | Binary | Recurrent CNVs | IR-score filtering to avoid spurious CNVRs | R | Fisher’s exact test; IR-score refinement | Biological specificity;  feature ranking-based true/false positive filtering; reduced false positives | No quantitative; no covariates | No | https://github.com/mahdieh1/PeakCNV/ | (88) |
| PLINK | Pre-called CNV segment lists | Case-control; quantitative; family-based | Binary; quantitative | Rare; common CNVs | Extensive QC filters; no explicit uncertainty modelling | CLI | Fisher’s exact test; permutation test; regression; CNV burden tests | Integrated GWAS pipeline; flexible tests; supports covariates | No uncertainty handling; no CNV discovery | No | https://zzz.bwh.harvard.edu/plink/download.shtml#download  https://github.com/chrchang/plink-ng/blob/master/1.9/plink_cnv.c | (89, 90) |
| R-GADA | SNP/aCGH intensity data | Case-control | Binary | Any CNV detectable by arrays | Fast GADA segmentation; population stratification adjustment | R | chi-square; regression; LRT (via multiCNVassoc function) | Fast, scalable; full pipeline; ancestry correction; covariate adjustment | Array only; basic association tests; no uncertainty; in practice, predominantly used for CNV detection rather than association testing | Yes | https://github.com/isglobal-brge/R-GADA | (10) |
| SNATCNV | Pre-called CNV segments (gene list file optional for annotation) | Case-control | Binary | Recurrent CNVs | Single-base scanning; no uncertainty modelling | CLI | Single-nucleotide Fisher’s exact test | Precise subregion discovery in large CNVs | Requires large cohorts; no covariates; limited distribution | No | https://github.com/hamidrokny/SNATCNV | (91) |
| Wham | BAM | Case-control (limited) | Binary | Any CNV | Likelihood scores | CLI | Built-in LRT | Unified calling + association | Wham is primarily an SV caller; its association functionality is limited and not widely validated for GWAS | Yes | https://github.com/zeeev/wham | (55) |
| Abbreviations: aCGH = array comparative genomic hybridisation; BAM = Binary Alignment Map; CLI = command line interface; CNV = copy number variation; CNVR = copy number variation region; eQTL = expression quantitative trait locus; EM = expectation maximisation; GLM = generalised linear model; GUI = graphical user interface; GWAS = genome-wide association study; IR-score = independence ranking score; LRT = likelihood ratio test; LRS = likelihood ratio selection; MLPA = multiplex ligation-dependent probe amplification; NGS = next-generation sequencing; QC = quality control; R = R statistical environment; RvTests = Rare Variant Tests framework; SNP = single nucleotide polymorphism; UKB = UK biobank; VCF = Variant Call Format | | | | | | | | | | | | |

**Reference**

1.	Litmaps. Litmaps (Version 2025-01-16) [Visualization purposes]. 2024.

2.	Olshen AB, Venkatraman ES, Lucito R, Wigler M. Circular binary segmentation for the analysis of array-based DNA copy number data. Biostatistics. 2004;5(4):557-72.

3.	Jeng XJ, Cai TT, Li H. Optimal Sparse Segment Identification with Application in Copy Number Variation Analysis. J Am Stat Assoc. 2010;105(491):1156-66.

4.	Darvishi K. Application of Nexus copy number software for CNV detection and analysis. Curr Protoc Hum Genet. 2010;Chapter 4:Unit 4.14.1-28.

5.	Niu YS, Zhang H. THE SCREENING AND RANKING ALGORITHM TO DETECT DNA COPY NUMBER VARIATIONS. Ann Appl Stat. 2012;6(3):1306-26.

6.	Morganella S, Cerulo L, Viglietto G, Ceccarelli M. VEGA: variational segmentation for copy number detection. Bioinformatics. 2010;26(24):3020-7.

7.	Pinto D, Pagnamenta AT, Klei L, Anney R, Merico D, Regan R, et al. Functional impact of global rare copy number variation in autism spectrum disorders. Nature. 2010;466(7304):368-72.

8.	Wang K, Li M, Hadley D, Liu R, Glessner J, Grant SF, et al. PennCNV: an integrated hidden Markov model designed for high-resolution copy number variation detection in whole-genome SNP genotyping data. Genome Res. 2007;17(11):1665-74.

9.	Colella S, Yau C, Taylor JM, Mirza G, Butler H, Clouston P, et al. QuantiSNP: an Objective Bayes Hidden-Markov Model to detect and accurately map copy number variation using SNP genotyping data. Nucleic Acids Res. 2007;35(6):2013-25.

10.	Pique-Regi R, Cáceres A, González JR. R-Gada: a fast and flexible pipeline for copy number analysis in association studies. BMC Bioinformatics. 2010;11:380.

11.	Backenroth D, Homsy J, Murillo LR, Glessner J, Lin E, Brueckner M, et al. CANOES: detecting rare copy number variants from whole exome sequencing data. Nucleic Acids Res. 2014;42(12):e97.

12.	Packer JS, Maxwell EK, O'Dushlaine C, Lopez AE, Dewey FE, Chernomorsky R, et al. CLAMMS: a scalable algorithm for calling common and rare copy number variants from exome sequencing data. Bioinformatics. 2016;32(1):133-5.

13.	Krumm N, Sudmant PH, Ko A, O'Roak BJ, Malig M, Coe BP, et al. Copy number variation detection and genotyping from exome sequence data. Genome Res. 2012;22(8):1525-32.

14.	Li J, Lupat R, Amarasinghe KC, Thompson ER, Doyle MA, Ryland GL, et al. CONTRA: copy number analysis for targeted resequencing. Bioinformatics. 2012;28(10):1307-13.

15.	Magi A, Tattini L, Cifola I, D'Aurizio R, Benelli M, Mangano E, et al. EXCAVATOR: detecting copy number variants from whole-exome sequencing data. Genome Biol. 2013;14(10):R120.

16.	D'Aurizio R, Pippucci T, Tattini L, Giusti B, Pellegrini M, Magi A. Enhanced copy number variants detection from whole-exome sequencing data using EXCAVATOR2. Nucleic Acids Res. 2016;44(20):e154.

17.	Samarakoon PS, Sorte HS, Kristiansen BE, Skodje T, Sheng Y, Tjønnfjord GE, et al. Identification of copy number variants from exome sequence data. BMC Genomics. 2014;15(1):661.

18.	Sathirapongsasuti JF, Lee H, Horst BA, Brunner G, Cochran AJ, Binder S, et al. Exome sequencing-based copy-number variation and loss of heterozygosity detection: ExomeCNV. Bioinformatics. 2011;27(19):2648-54.

19.	Love MI, Myšičková A, Sun R, Kalscheuer V, Vingron M, Haas SA. Modeling read counts for CNV detection in exome sequencing data. Stat Appl Genet Mol Biol. 2011;10(1).

20.	Plagnol V, Curtis J, Epstein M, Mok KY, Stebbings E, Grigoriadou S, et al. A robust model for read count data in exome sequencing experiments and implications for copy number variant calling. Bioinformatics. 2012;28(21):2747-54.

21.	Gambin T, Akdemir ZC, Yuan B, Gu S, Chiang T, Carvalho CMB, et al. Homozygous and hemizygous CNV detection from exome sequencing data in a Mendelian disease cohort. Nucleic Acids Res. 2017;45(4):1633-48.

22.	Wang C, Evans JM, Bhagwate AV, Prodduturi N, Sarangi V, Middha M, et al. PatternCNV: a versatile tool for detecting copy number changes from exome sequencing data. Bioinformatics. 2014;30(18):2678-80.

23.	Fromer M, Moran JL, Chambert K, Banks E, Bergen SE, Ruderfer DM, et al. Discovery and statistical genotyping of copy-number variation from whole-exome sequencing depth. Am J Hum Genet. 2012;91(4):597-607.

24.	Roller E, Ivakhno S, Lee S, Royce T, Tanner S. Canvas: versatile and scalable detection of copy number variants. Bioinformatics. 2016;32(15):2375-7.

25.	Klambauer G, Schwarzbauer K, Mayr A, Clevert DA, Mitterecker A, Bodenhofer U, et al. cn.MOPS: mixture of Poissons for discovering copy number variations in next-generation sequencing data with a low false discovery rate. Nucleic Acids Res. 2012;40(9):e69.

26.	Fitzgerald T, Birney E. CNest: A novel copy number association discovery method uncovers 862 new associations from 200,629 whole-exome sequence datasets in the UK Biobank. Cell Genomics. 2022;2(8):100167.

27.	Boeva V, Popova T, Bleakley K, Chiche P, Cappo J, Schleiermacher G, et al. Control-FREEC: a tool for assessing copy number and allelic content using next-generation sequencing data. Bioinformatics. 2011;28(3):423-5.

28.	Chen X, Schulz-Trieglaff O, Shaw R, Barnes B, Schlesinger F, Källberg M, et al. Manta: rapid detection of structural variants and indels for germline and cancer sequencing applications. Bioinformatics. 2015;32(8):1220-2.

29.	Demidov G, Sturm M, Ossowski S. ClinCNV: multi-sample germline CNV detection in NGS data. bioRxiv. 2022:2022.06.10.495642.

30.	Behera S, Catreux S, Rossi M, Truong S, Huang Z, Ruehle M, et al. Comprehensive genome analysis and variant detection at scale using DRAGEN. Nature Biotechnology. 2025;43(7):1177-91.

31.	Babadi M, Fu JM, Lee SK, Smirnov AN, Gauthier LD, Walker M, et al. GATK-gCNV enables the discovery of rare copy number variants from exome sequencing data. Nat Genet. 2023;55(9):1589-97.

32.	Talevich E, Shain AH, Botton T, Bastian BC. CNVkit: Genome-Wide Copy Number Detection and Visualization from Targeted DNA Sequencing. PLOS Computational Biology. 2016;12(4):e1004873.

33.	Jiang Y, Oldridge DA, Diskin SJ, Zhang NR. CODEX: a normalization and copy number variation detection method for whole exome sequencing. Nucleic Acids Res. 2015;43(6):e39.

34.	Jiang Y, Wang R, Urrutia E, Anastopoulos IN, Nathanson KL, Zhang NR. CODEX2: full-spectrum copy number variation detection by high-throughput DNA sequencing. Genome Biology. 2018;19(1):202.

35.	Fowler A. DECoN: A Detection and Visualization Tool for Exonic Copy Number Variants. Methods Mol Biol. 2022;2493:77-88.

36.	Xi R, Lee S, Xia Y, Kim TM, Park PJ. Copy number analysis of whole-genome data using BIC-seq2 and its application to detection of cancer susceptibility variants. Nucleic Acids Res. 2016;44(13):6274-86.

37.	Chen K, Wallis JW, McLellan MD, Larson DE, Kalicki JM, Pohl CS, et al. BreakDancer: an algorithm for high-resolution mapping of genomic structural variation. Nat Methods. 2009;6(9):677-81.

38.	Abyzov A, Li S, Kim DR, Mohiyuddin M, Stütz AM, Parrish NF, et al. Analysis of deletion breakpoints from 1,092 humans reveals details of mutation mechanisms. Nature Communications. 2015;6(1):7256.

39.	Lam HYK, Mu XJ, Stütz AM, Tanzer A, Cayting PD, Snyder M, et al. Nucleotide-resolution analysis of structural variants using BreakSeq and a breakpoint library. Nature Biotechnology. 2010;28(1):47-55.

40.	Abyzov A, Urban AE, Snyder M, Gerstein M. CNVnator: an approach to discover, genotype, and characterize typical and atypical CNVs from family and population genome sequencing. Genome Res. 2011;21(6):974-84.

41.	Popic V, Rohlicek C, Cunial F, Hajirasouliha I, Meleshko D, Garimella K, et al. Cue: a deep-learning framework for structural variant discovery and genotyping. Nat Methods. 2023;20(4):559-68.

42.	Rausch T, Zichner T, Schlattl A, Stütz AM, Benes V, Korbel JO. DELLY: structural variant discovery by integrated paired-end and split-read analysis. Bioinformatics. 2012;28(18):i333-i9.

43.	Zhu M, Need AC, Han Y, Ge D, Maia JM, Zhu Q, et al. Using ERDS to infer copy-number variants in high-coverage genomes. Am J Hum Genet. 2012;91(3):408-21.

44.	Michaelson JJ, Sebat J. forestSV: structural variant discovery through statistical learning. Nature Methods. 2012;9(8):819-21.

45.	Cameron DL, Schröder J, Penington JS, Do H, Molania R, Dobrovic A, et al. GRIDSS: sensitive and specific genomic rearrangement detection using positional de Bruijn graph assembly. Genome Res. 2017;27(12):2050-60.

46.	Dharanipragada P, Vogeti S, Parekh N. iCopyDAV: Integrated platform for copy number variations-Detection, annotation and visualization. PLoS One. 2018;13(4):e0195334.

47.	Magi A, Benelli M, Yoon S, Roviello F, Torricelli F. Detecting common copy number variants in high-throughput sequencing data by using JointSLM algorithm. Nucleic Acids Res. 2011;39(10):e65.

48.	Layer RM, Chiang C, Quinlan AR, Hall IM. LUMPY: a probabilistic framework for structural variant discovery. Genome Biology. 2014;15(6):R84.

49.	Ye K, Schulz MH, Long Q, Apweiler R, Ning Z. Pindel: a pattern growth approach to detect break points of large deletions and medium sized insertions from paired-end short reads. Bioinformatics. 2009;25(21):2865-71.

50.	Yoon S, Xuan Z, Makarov V, Ye K, Sebat J. Sensitive and accurate detection of copy number variants using read depth of coverage. Genome Res. 2009;19(9):1586-92.

51.	Miller CA, Hampton O, Coarfa C, Milosavljevic A. ReadDepth: a parallel R package for detecting copy number alterations from short sequencing reads. PLoS One. 2011;6(1):e16327.

52.	Bartenhagen C, Dugas M. Robust and exact structural variation detection with paired-end and soft-clipped alignments: SoftSV compared with eight algorithms. Briefings in Bioinformatics. 2015;17(1):51-62.

53.	Zhang J, Wang J, Wu Y. An improved approach for accurate and efficient calling of structural variations with low-coverage sequence data. BMC Bioinformatics. 2012;13 Suppl 6(Suppl 6):S6.

54.	Eisfeldt J, Vezzi F, Olason P, Nilsson D, Lindstrand A. TIDDIT, an efficient and comprehensive structural variant caller for massive parallel sequencing data. F1000Res. 2017;6:664.

55.	Kronenberg ZN, Osborne EJ, Cone KR, Kennedy BJ, Domyan ET, Shapiro MD, et al. Wham: Identifying Structural Variants of Biological Consequence. PLoS Comput Biol. 2015;11(12):e1004572.

56.	Chiang T, Liu X, Wu TJ, Hu J, Sedlazeck FJ, White S, et al. Atlas-CNV: a validated approach to call single-exon CNVs in the eMERGESeq gene panel. Genet Med. 2019;21(9):2135-44.

57.	Johansson LF, van Dijk F, de Boer EN, van Dijk-Bos KK, Jongbloed JD, van der Hout AH, et al. CoNVaDING: Single Exon Variation Detection in Targeted NGS Data. Hum Mutat. 2016;37(5):457-64.

58.	Cabello-Aguilar S, Vendrell JA, Van Goethem C, Brousse M, Gozé C, Frantz L, et al. ifCNV: A novel isolation-forest-based package to detect copy-number variations from various targeted NGS datasets. Mol Ther Nucleic Acids. 2022;30:174-83.

59.	Povysil G, Tzika A, Vogt J, Haunschmid V, Messiaen L, Zschocke J, et al. panelcn.MOPS: Copy-number detection in targeted NGS panel data for clinical diagnostics. Hum Mutat. 2017;38(7):889-97.

60.	Jiang T, Liu Y, Jiang Y, Li J, Gao Y, Cui Z, et al. Long-read-based human genomic structural variation detection with cuteSV. Genome Biology. 2020;21(1):189.

61.	Cretu Stancu M, van Roosmalen MJ, Renkens I, Nieboer MM, Middelkamp S, de Ligt J, et al. Mapping and phasing of structural variation in patient genomes using nanopore sequencing. Nature Communications. 2017;8(1):1326.

62.	Tham CY, Tirado-Magallanes R, Goh Y, Fullwood MJ, Koh BTH, Wang W, et al. NanoVar: accurate characterization of patients’ genomic structural variants using low-depth nanopore sequencing. Genome Biology. 2020;21(1):56.

63.	Gong L, Wong CH, Cheng WC, Tjong H, Menghi F, Ngan CY, et al. Picky comprehensively detects high-resolution structural variants in nanopore long reads. Nat Methods. 2018;15(6):455-60.

64.	Sedlazeck FJ, Rescheneder P, Smolka M, Fang H, Nattestad M, von Haeseler A, et al. Accurate detection of complex structural variations using single-molecule sequencing. Nature Methods. 2018;15(6):461-8.

65.	Smolka M, Paulin LF, Grochowski CM, Horner DW, Mahmoud M, Behera S, et al. Detection of mosaic and population-level structural variants with Sniffles2. Nature Biotechnology. 2024;42(10):1571-80.

66.	Heller D, Vingron M. SVIM-asm: structural variant detection from haploid and diploid genome assemblies. Bioinformatics. 2020;36(22-23):5519-21.

67.	Goel M, Sun H, Jiao W-B, Schneeberger K. SyRI: finding genomic rearrangements and local sequence differences from whole-genome assemblies. Genome Biology. 2019;20(1):277.

68.	English AC, Salerno WJ, Reid JG. PBHoney: identifying genomic variants via long-read discordance and interrupted mapping. BMC Bioinformatics. 2014;15(1):180.

69.	Zhang Z, Cheng H, Hong X, Di Narzo AF, Franzen O, Peng S, et al. EnsembleCNV: an ensemble machine learning algorithm to identify and genotype copy number variation using SNP array data. Nucleic Acids Res. 2019;47(7):e39.

70.	Pounraja VK, Jayakar G, Jensen M, Kelkar N, Girirajan S. A machine-learning approach for accurate detection of copy number variants from exome sequencing. Genome Res. 2019;29(7):1134-43.

71.	Wong K, Keane TM, Stalker J, Adams DJ. Enhanced structural variant and breakpoint detection using SVMerge by integration of multiple detection methods and local assembly. Genome Biology. 2010;11(12):R128.

72.	Zarate S, Carroll A, Mahmoud M, Krasheninina O, Jun G, Salerno WJ, et al. Parliament2: Accurate structural variant calling at scale. Gigascience. 2020;9(12).

73.	Mohiyuddin M, Mu JC, Li J, Bani Asadi N, Gerstein MB, Abyzov A, et al. MetaSV: an accurate and integrative structural-variant caller for next generation sequencing. Bioinformatics. 2015;31(16):2741-4.

74.	Lam HY, Pan C, Clark MJ, Lacroute P, Chen R, Haraksingh R, et al. Detecting and annotating genetic variations using the HugeSeq pipeline. Nat Biotechnol. 2012;30(3):226-9.

75.	Becker T, Lee W-P, Leone J, Zhu Q, Zhang C, Liu S, et al. FusorSV: an algorithm for optimally combining data from multiple structural variation detection methods. Genome Biology. 2018;19(1):38.

76.	Dierckxsens N, Li T, Vermeesch JR, Xie Z. A benchmark of structural variation detection by long reads through a realistic simulated model. Genome Biol. 2021;22(1):342.

77.	Fang L, Hu J, Wang D, Wang K. NextSV: a meta-caller for structural variants from low-coverage long-read sequencing data. BMC Bioinformatics. 2018;19(1):180.

78.	Jeffares DC, Jolly C, Hoti M, Speed D, Shaw L, Rallis C, et al. Transient structural variations have strong effects on quantitative traits and reproductive isolation in fission yeast. Nat Commun. 2017;8:14061.

79.	Korn JM, Kuruvilla FG, McCarroll SA, Wysoker A, Nemesh J, Cawley S, et al. Integrated genotype calling and association analysis of SNPs, common copy number polymorphisms and rare CNVs. Nat Genet. 2008;40(10):1253-60.

80.	Subirana I, Diaz-Uriarte R, Lucas G, Gonzalez JR. CNVassoc: Association analysis of CNV data using R. BMC Med Genomics. 2011;4:47.

81.	da Silva V, Ramos M, Groenen M, Crooijmans R, Johansson A, Regitano L, et al. CNVRanger: association analysis of CNVs with gene expression and quantitative phenotypes. Bioinformatics. 2020;36(3):972-3.

82.	Kim JH, Hu HJ, Yim SH, Bae JS, Kim SY, Chung YJ. CNVRuler: a copy number variation-based case-control association analysis tool. Bioinformatics. 2012;28(13):1790-2.

83.	Jeng J, Wu Q, Li H. A Statistical Method for Identifying Trait-Associated Copy Number Variants. Hum Hered. 2015;79(3-4):147-56.

84.	Barnes C, Plagnol V, Fitzgerald T, Redon R, Marchini J, Clayton D, et al. A robust statistical method for case-control association testing with copy number variation. Nat Genet. 2008;40(10):1245-52.

85.	Larsen SJ, do Canto LM, Rogatto SR, Baumbach J. CoNVaQ: a web tool for copy number variation-based association studies. BMC Genomics. 2018;19(1):369.

86.	Glessner JT, Li J, Hakonarson H. ParseCNV integrative copy number variation association software with quality tracking. Nucleic Acids Res. 2013;41(5):e64.

87.	Glessner JT, Li J, Liu Y, Khan M, Chang X, Sleiman PMA, et al. ParseCNV2: efficient sequencing tool for copy number variation genome-wide association studies. European Journal of Human Genetics. 2023;31(3):304-12.

88.	Labani M, Afrasiabi A, Beheshti A, Lovell NH, Alinejad-Rokny H. PeakCNV: A multi-feature ranking algorithm-based tool for genome-wide copy number variation-association study. Comput Struct Biotechnol J. 2022;20:4975-83.

89.	Purcell S, Neale B, Todd-Brown K, Thomas L, Ferreira MA, Bender D, et al. PLINK: a tool set for whole-genome association and population-based linkage analyses. Am J Hum Genet. 2007;81(3):559-75.

90.	Chang CC, Chow CC, Tellier LC, Vattikuti S, Purcell SM, Lee JJ. Second-generation PLINK: rising to the challenge of larger and richer datasets. Gigascience. 2015;4:7.

91.	Alinejad-Rokny H, Heng JIT, Forrest ARR. Brain-Enriched Coding and Long Non-coding RNA Genes Are Overrepresented in Recurrent Neurodevelopmental Disorder CNVs. Cell Rep. 2020;33(4):108307.
