# Supplementary material for: Computational strategies for copy number variation detection, disease association, and beyond
Source: Genome Biol. 2026 Jul 10;27:287. doi: 10.1186/s13059-026-04189-6 (PMC13551817; doi:10.1186/s13059-026-04189-6)
Supplement: Supplementary file 2 [file 13059_2026_4189_MOESM3_ESM.docx]

**Note S1. Platforms and methodologies for copy number variation detection**

**Cytogenetic and microarray-based methods**

Classical cytogenetic techniques, such as Giemsa staining, G-banding, and fluorescence in situ hybridisation (FISH), were the first tools used to detect large chromosomal abnormalities (typically >5 megabases) but lack the resolution to identify submicroscopic copy number variations (CNVs) (reviewed in Gordeeva et al. (1) and Ho et al. (2)). In the 2000s, microarrays (array-based comparative genomic hybridisation (aCGH) and single nucleotide polymorphism (SNP)-array) emerged as the leading technology for detecting CNVs, offering resolution at the level of tens of kilobases (3, 4) and enabling the discovery of large CNVs strongly associated with developmental disorders, which led the American College of Medical Genetics to recommend chromosomal microarray as a first-tier clinical diagnostic test for unexplained developmental disorders or congenital anomalies (5-7).

**Sequencing-based approaches**

Sequencing-based methods have significantly advanced CNV detection because of their enhanced sensitivity compared with array platforms. Short-read sequencing (SR-seq) methods (genome sequencing (GS) and exome sequencing (ES)) are not restricted to the specific regions covered by arrays. They have a greater capacity to discover new CNVs and provide higher resolution in predicting both breakpoints and smaller CNVs (8, 9). To address the coverage limitations of GS and ES, reduce costs, and simplify the analysis of the vast data produced by GS and ES, molecular diagnostic laboratories have routinely adopted custom or commercially targeted gene panels (10, 11). However, SR-seq techniques fail to encompass the entire spectrum of genomic variation. These methods struggle with regions of low complexity, such as repeats, and highly polymorphic areas, such as the human leukocyte antigen region. Furthermore, numerous diseases are linked to repeats that exceed the resolution of SR-seq. Although these techniques have proven quite successful in detecting single nucleotide variants and small indels, there is evidence suggesting that we have greatly underestimated the extent and complexity of structural variations, including CNVs, within the genome (12-17).

Many innovative technologies have attempted to address the limitations associated with short reads (reviewed by Ho et al. (2) and Mahmoud et al. (9)). Long-range genomic mapping methods, such as 10x Genomics Linked-Reads, Strand-seq, and Hi-C, are built upon short reads by identifying long-range associations between short-read pairs mapped at distant locations.

Alternatively, single-molecule approaches, such as long-read sequencing technologies by Pacific Biosciences (PacBio) and Oxford Nanopore Technologies (ONT), produce continuous reads ranging from tens to hundreds of kilobases in length. This feature enables the direct detection of a wide range of CNVs and improves the alignment of distinct reads in repetitive regions. Separately, optical mapping, as implemented by Bionano Genomics, does not rely on sequencing but instead generates high-resolution physical maps of the genome based on labelled DNA molecules (2, 9, 18).

Recent advances have enabled the detection of CNVs using RNA sequencing data. Advanced methods allow CNV detection at the single-cell level, especially in cancer research, providing insights into tumour heterogeneity, clonal evolution, and the functional impact of genomic alterations on gene expression (19-24).

**Note S2. Overview of copy number variation detection strategies across array- and sequencing-based platforms**

**Array-based comparative genomic hybridisation data**

Several common tools and algorithms are used to detect copy number variations (CNVs) using array-based comparative genomic hybridisation (aCGH) data, which can be broadly categorised as global or local segmentation methods (1, 25). Global segmentation methods are algorithms that analyse the entire genome to identify candidate CNV segments by comparing each potential segment with the full genomic background (26). These methods, such as circular binary segmentation (as implemented in DNAcopy) (27), ADM-2, and Nexus (28), often rely on recursive procedures to detect the change points. They are effective in identifying large-scale aberrations and are generally robust and computationally efficient. However, they may be less sensitive to small or subtle variations, particularly in high-resolution data.

In contrast, local segmentation methods focus on identifying CNVs based on localised statistical evidence rather than global comparisons. Techniques such as SaRa (29) and likelihood ratio selection (30) scan the genome using sliding windows or scan statistics to detect regions with significant deviations. These methods are theoretically grounded to maximise the detection power for small-scale or sparse events, particularly on high-resolution platforms. Although more sensitive than global methods, they are more prone to false positives, especially when the data deviate from normality (1, 26, 27, 31, 32).

**Single nucleotide polymorphism-array data**

Various models have been developed to detect CNVs from single nucleotide polymorphism (SNP)-array data using statistical, heuristic, and deep learning-based approaches. Hidden Markov models (HMMs) are widely used to model hidden copy number states across the genome. Tools such as PennCNV (33) and QuantiSNP (34) are included in this category. PennCNV incorporates the log R ratio (LRR), B-allele frequency (BAF), population allele frequencies, and the distance between neighbouring SNPs, and applies state-specific and distance-dependent transition probabilities (33). QuantiSNP uses an objective Bayes HMM framework to assign probabilistic CNV states based on LRR and BAF (34). In contrast, segmentation algorithms, such as R-GADA (35), divide the genome into segments and infer copy number states based on signal discontinuities. R-GADA applies a sparse Bayesian learning model, followed by backward elimination, to refine breakpoints (35). Ensemble methods aim to integrate the strengths of multiple callers (Section 3.2.1); for instance, EnsembleCNV combines outputs from PennCNV, QuantiSNP, and iPattern, applying batch effect correction, CNV call combination, re-genotyping, and boundary refinement (36). Additionally, Gaussian mixture models, as implemented in iPattern, utilise normalised signal intensities across samples to jointly detect CNVs, particularly those with high allele frequencies (37).

**Short-read sequencing data**

CNV detection from short-read sequencing data is generally approached using five main strategies. The first is paired-end mapping or the read pair method. This strategy examines the orientation and spacing between paired-end or mate-pair reads. Read pair-based tools use two strategies: model-based and clustering. The model-based strategy employs a probability test to identify abnormal distances between read pairs, whereas the clustering approach uses a predetermined distance to detect inconsistent readings (8, 38).

The second approach, split read, examines read pairings in which one aligned read is uniquely mapped, whereas the other fails to map or only partially maps. Split read detection strategies aim to identify reads that include the breakpoints of structural variations (SVs) and may be split into segments. Partially mapped reads are broken into several pieces using split read-based tools, with the initial and final fragments remapped independently (8, 38).

The third strategy involves read depth, which detects CNVs by measuring the number of sequencing reads aligned to specific genomic intervals. Read depth-based approaches typically calculate read counts in fixed-size or variable-size non-overlapping genomic windows (38). Mapping, normalisation, segmentation, and copy number estimation are crucial stages. During the mapping phase, short reads are aligned to a reference genome, and the read depth profile is obtained by counting the number of reads mapped with sufficient quality to each genomic window. During normalisation, systematic biases in read depth arising from variations in GC content, sequence mappability, or repetitive elements are corrected to improve accuracy. In the segmentation stage, the genome of each individual is partitioned into contiguous candidate CNV segments that exhibit similar normalised read depth values. Different algorithms are often applied to detect change points corresponding to copy number alterations. Finally, copy number state estimation is performed by assigning each segment a copy number value based on the normalised read depth profile, thereby identifying the genomic regions affected by deletions or duplications (8, 39). From a mathematical perspective, it is worth mentioning that once mapped and normalised, the read depth data obtained from next generation sequencing (NGS) studies exhibit similarities to the probe log ratios derived from aCGH data. Therefore, traditional methods for identifying CNV areas in aCGH data may be adapted and used for NGS read depth data (8). This step is critical because it significantly increases the accuracy of CNV detection by identifying these segments, which can then be classified as amplification or deletion. Several segmentation algorithms and approaches have been developed for CNV detection (see Note S3).

The fourth approach is *de novo* assembly (AS), which reconstructs contigs from overlapping reads without using a reference genome. CNV segments can be inferred by aligning the contigs to a reference. AS methods provide an unbiased platform for detecting novel variants across a broad size range, although they are computationally intensive and perform poorly in repetitive regions (8).

Finally, the fifth approach is the combinatorial approach, which integrates multiple types of evidence to improve the sensitivity, specificity, and resolution of CNV calling (2, 8).

**Long-read sequencing data**

SV detection, including CNVs, using long-read sequencing data relies on several computational strategies that leverage the unique advantages of long reads, such as their ability to span repetitive regions and provide long-range information (reviewed in Ahsan et al. (40)). One of the main approaches is alignment-based detection, in which individual long reads are aligned to a reference genome, and signatures such as read depth changes, split reads, soft-clipped bases, and large alignment gaps are extracted. These signatures are then clustered across reads to form consensus CNV calls. Tools such as cuteSV, Sniffles, Sniffles2, PBHoney, NanoSV, NanoVar, and Picky exemplify this strategy, which is generally rapid and performs well even with relatively low sequencing coverage. However, the results can be sensitive to the choice of aligner and sequencing error profile and may struggle to resolve complex CNVs in repetitive regions (41-50).

Another widely used strategy is assembly-based CNV detection. In this approach, long reads are first assembled into contigs through *de novo* or reference-guided assembly. These contigs are then aligned with a reference genome to identify differences. Tools such as SVIM-asm, SyRI and pbsv follow this approach. Assembly-based techniques provide high accuracy in breakpoint detection and are particularly useful for identifying large insertions, deletions, and complex rearrangements, particularly in repetitive regions. However, they are computationally intensive and typically require a higher sequencing depth, usually above 20x, to be effective (51-53).

**Note S3. Segmentation algorithms and approaches for copy number variation detection**

Segmentation algorithms are vital for detecting and analysing copy number variations (CNVs) in genomic data. Their main objective is to locate breakpoints where DNA copy number shifts occur, thereby dividing the noisy observed genomic signals into contiguous segments that ideally represent distinct underlying copy number states. The following are some key segmentation algorithms and methodologies commonly used in CNV analysis.

**Circular binary segmentation**

Circular binary segmentation (CBS) is an improved version of binary segmentation designed to detect breakpoints in copy number data. It models copy number measurements as a sequence of random variables and recursively identifies change points, where adjacent genomic segments show statistically significant differences. Starting from the entire chromosome, CBS splits the data into segments by locating breakpoints using a statistical test, which is often based on the maximum t-statistic. A breakpoint is called if the associated P-value falls below a predefined threshold (commonly 0.01). To prevent oversegmentation, a pruning step is applied to reduce the number of segments (27, 54). CBS has been implemented in several CNV detection tools, including ReadDepth (55).

**Hidden Markov models**

Hidden Markov models (HMMs)-based methods model the hidden copy number states underlying the observed genomic data, such as probe intensities or read depths (8). These models are defined by parameters, including the initial state distribution, transition probabilities between copy number states, and emission probability functions within each state. Parameter learning is typically performed using the Baum-Welch algorithm, employing an expectation-maximisation algorithm, while the most probable sequence of hidden states is inferred using the Viterbi algorithm to identify CNV segments. Some tools allow users to specify the number of hidden states and constrain transitions to reflect biologically plausible events (e.g. disallowing transitions directly from deletion to amplification) (54). A well-known HMM-based CNV detection tool is XHMM (56).

**Shifting level models**

Shifting level models are designed to model noisy sequential data characterised by abrupt changes in the mean, such as those observed in read depth signals for CNV detection. These models can often be framed and solved using classical HMM algorithms, including Baum-Welch for parameter training and Viterbi for inferring the most probable sequence of states (57, 58). Notable variants include JointSLM (59), which performs joint segmentation across multiple read depth profiles, and HSLM (60), which is adapted for spatially sparse data typical of exome sequencing.

**Mean shift-based segmentation**

The mean shift-based (MSB) approach, originally developed for image processing, has been adapted to segment read depth signals for CNV detection. In particular, tools such as CNVnator implement the MSB approach by dividing the genome into equal-sized, non-overlapping bins and using the number of mapped reads in each bin as the read depth signal. The segmentation process relies on the mean-shift algorithm, in which each data point is iteratively shifted towards regions of local density maxima along a mean-shift vector. Breakpoints between segments are identified where neighbouring vectors diverge but do not point towards each other, marking the transitions between distinct copy number states. To detect CNVs of varying sizes, a multi-bandwidth partitioning strategy is employed (61, 62).

**Variational-based segmentation approaches**

These segmentation methods are built on discontinuity-adaptive variational models, such as the Mumford-Shah model (63), which originated from image processing. They work by minimising an energy functional that balances the fit between the segmentation and the observed read depth data, while penalising overly complex or fragmented solutions (e.g., by limiting the number or length of segments) (64, 65). In VEGA, a bottom-up region-growing approach is applied. Small homogeneous regions are progressively merged into larger ones based on the similarity in the signal, guided by a data-driven regularisation parameter that controls the level of merging (65).

**Likelihood ratio selection**

Likelihood ratio selection (LRS) is designed to identify sparse and short CNV segments within long, noisy genomic read depth signals, such as those generated by genome sequencing. It proposes the use of a mean-matching variance-stabilising transformation to convert the problem of sparse segment identification for count data (such as read depth) into a Gaussian data problem. The LRS procedure then scans the genome with candidate intervals, calculates the likelihood ratio statistics, and iteratively selects intervals with the largest statistics while removing overlapping ones (30, 66).

**Isolation forest-based methods**

These approaches apply machine learning techniques, particularly the isolation forest (IF) algorithm, to detect CNVs as genomic data anomalies. IF constructs random binary trees to isolate observations, with anomalies typically requiring fewer splits and thus appearing closer to the root. Each genomic bin is assigned an anomaly score based on its average path length across the trees (67, 68). For example, ifCNV offers a reference-free implementation, it automatically constructs an internal reference from the input data and combines two IFs with a tailored scoring function to improve accuracy (68).

**Note S4. Platform-specific benchmarking of copy number variation detection tools**

For copy number variation (CNV) detection in array-based comparative genomic hybridisation (aCGH) data, a stepwise approach that combines different segmentation strategies is highly effective. Utilising circular binary segmentation as an initial step enables the robust identification of larger CNV segments, given its resilience to heavy-tailed noise and deviations from normality. This can be followed by the application of local segmentation methods, such as likelihood ratio selection, along with appropriate post-processing filters to remove small-width aberrations and minimise false positives. Integrating these complementary approaches can significantly improve both the power and reproducibility of CNV calls and increase concordance across technical replicates (25). aCGH was one of the first approaches to enable higher-resolution CNV detection, typically around ~100 kb (69). However, because aCGH lacks B-allele frequency (BAF) information, has relatively low resolution and coarse breakpoint precision, and is less scalable than newer platforms, it has been replaced by single nucleotide polymorphism (SNP) arrays and sequencing-based approaches in CNV association studies, although it may serve as validation alongside quantitative PCR or multiplex ligation-dependent probe amplification.

A similar principle applies to SNP-array data, where performance depends on both the algorithm choice and array design, particularly the probe density and distribution. Although high-density arrays can theoretically detect CNVs as small as ~20 kb, reliable detection in practice is generally limited to larger events (approximately ≥25 kb for deletions and ≥50 kb for duplications), with smaller calls being more prone to false positives unless supported by dense probe coverage (70-72). Hidden Markov model-based methods, such as PennCNV and QuantiSNP, generally offer higher precision and prediction validity, whereas other segmentation algorithms, such as R-GADA, tend to yield higher recall at the cost of increased false positives (73, 74). For high precision and reliability, PennCNV is consistently recommended as the most reliable method, achieving the highest precision and F1 score across various thresholds and CNV types (deletions and duplications), whereas R-GADA is preferred for sensitivity-focused studies aiming to capture as many true positives as possible (73, 74). Although EnsembleCNV, a newer ensemble method combining PennCNV, QuantiSNP, and iPattern calls, aims to balance performance, it can unexpectedly increase false positives, partly because of the lower precision of its underlying components, such as QuantiSNP and iPattern (74). Tool selection should also consider computational resources, as some methods (e.g., EnsembleCNV, QuantiSNP, and iPattern) require significantly more processing time and memory, making them less ideal for resource-limited environments (74).

Moving to genome sequencing (GS), which offers improved resolution and breakpoint accuracy, the same balance between sensitivity and specificity remains critical, but the sequencing context adds another layer of consideration (71, 75, 76). In short-read GS, variants as small as ~50 bp can be detected using breakpoint-based signals; however, read depth-based approaches are generally more reliable for CNVs larger than ~1 kb (76, 77). In contrast, ultra-low-coverage GS (e.g. ≤0.1×), substantially reduces resolution and is typically limited to the detection of large CNVs (≥1 Mb), with smaller events (e.g. <2 Mb) being more prone to false positives (78). For high sensitivity, tools such as LUMPY, CNVnator, and RDXplorer are effective, whereas LUMPY and Canvas are preferred for their low false discovery rates (FDR) (76). Another benchmarking study showed that DRAGEN HS (high-sensitivity mode) provides the highest sensitivity, whereas Cue delivers the highest precision, albeit with poor detection of small events (71). Although DRAGEN is commonly applied in GS workflows, its high-sensitivity modes have also been adapted for exome and targeted panel sequencing, with performance depending on the sequencing context and filtering strategies (79). The choice of tool must also consider the sequencing context. In genome bisulfite sequencing data, different aligner-caller combinations demonstrate optimal performance depending on the type of CNV. Specifically, the combinations of bwameth-DELLY and bwameth-BreakDancer have been reported to perform well for detecting deletions, whereas walt-CNVnator and bismarkbt2-CNVnator are preferable for identifying duplications (80). LUMPY generally stands out as the best overall tool for GS across various depths (5x to 50x) (76). For ultra-low coverage GS, BIC-seq2 or FREEC is suitable for large CNVs (≥1 Mb), with BIC-seq2 being favoured for smaller CNVs and sex chromosomes (78). The tool performance also varies according to the CNV type and size. For deletions, GRIDSS, LUMPY, SVseq2, SoftSV, and Manta are broadly effective, whereas large deletions are better detected by TIDDIT, forestSV, ERDS, and CNVnator (81). ERDS is generally highly accurate for deletions and works well with CNVnator for larger events (77). BreakDancer is particularly effective in detecting deletions of 1 kb or larger in GS (75). For duplications, Wham, SoftSV, and GRIDSS perform well, whereas CNVnator, ERDS, and iCopyDAV are effective for larger duplications (81). Similarly, Canvas, cn.MOPS, and ERDS offer strong performance for duplications with different size ranges (77). Combining complementary CNV callers is recommended to enhance overall accuracy. Using tools that exploit different signal types (e.g. Manta, Delly, ERDS, and CNVnator) can significantly improve precision and boost breakpoint, size, and genotype accuracy (81, 82). For clinical or diagnostic applications, a consensus among the top tools, such as GATK-gCNV, cn.MOPS, and CNVkit is advised (83). Some combinations, such as CNVnator and ERDS, resulted in a modest improvement in the FDR for deletions, although this came at the expense of reduced sensitivity. However, for clinically relevant CNVs, specifically rare genic events, this combination achieved a 0% FDR, with all calls confirmed (77). Similarly, combining BreakDancer and Delly showed minimal added value compared to that of BreakDancer alone (75).

The choice of CNV callers in exome sequencing (ES) follows the same rule; no single tool is optimal for all metrics. Differences between tools often reflect trade-offs between sensitivity and precision under default settings, although many methods provide quality scores that enable post hoc filtering, and integrated metrics, such as the F1 score, can offer more balanced comparisons. CNV detection in ES is inherently limited by its focus on protein-coding regions; therefore, breakpoints located in intronic or intergenic regions are often not captured (71, 84). Consequently, the detection resolution is typically defined at the exon level, with most tools performing optimally for CNVs spanning multiple exons (commonly ≥1-7 exons or ~1 kb and above), whereas sensitivity decreases substantially for smaller events involving only 1-4 exons (85, 86). For high sensitivity, tools such as EXCAVATOR2, exomeCopy, and ExomeDepth are often preferred (84-86). For applications requiring low false-positive rates or high precision, tools such as CONIFER, HMZDelFinder, cn.MOPS, and XHMM are more suitable (84-86), with CoNIFER reported to achieve a low false-positive rate in ES data; however, this may partly reflect its design, which limits the detection of very short CNVs (86). When detecting small CNVs, typically those involving one to seven exons, PatternCNV and CONTRA have shown comparable performance, and their intersection can improve precision (85). In scenarios involving smaller reference pools or variable coverage, the use of exomeCopy and ExCopyDepth, either individually or in combination, is recommended (86). Several tools, including ExomeDepth, XHMM, and CoNIFER, are widely used to detect rare CNVs in ES data. While ExomeDepth often performs well when larger reference sets are available, the differences between these methods primarily reflect trade-offs between sensitivity and specificity rather than targeting distinct classes of CNVs (84, 86). Owing to the limited overlap between different tools and the potential for high false-positive rates, the use of ensemble or consensus-based approaches is recommended. Intersecting results from multiple tools, such as PatternCNV and CONTRA, can increase the precision (85). Similarly, the intersection of ExomeCopy and ExCopyDepth is recommended for detecting short CNVs (86). In clinical or diagnostic settings, combining the outputs from tools such as GATK-gCNV, cn.MOPS, and CNVkit can enhance robustness and accuracy (83).

While CNV detection from targeted sequencing panels remains challenging because of restricted genomic coverage limited to predefined target regions, its suitability remains debatable (87, 88). Nevertheless, several tools have been developed to enable CNV inference in such data, particularly in clinical settings, where sensitivity is often prioritised to avoid missed diagnoses. Tools such as SOPHiA GENETICS DDM offer high sensitivity with no missed verified CNVs at the default settings, although the positive predictive value (PPV) is moderate. ExomeDepth generally provides a high PPV but with a lower sensitivity (87, 89). DECoN and panelcn.MOPS are frequently recommended because of their high sensitivity, particularly for single- and multi-exon CNVs. DECoN also achieves a strong balance between sensitivity and specificity when optimised (69, 87). GATK-gCNV and ClinCNV achieved the highest F1 scores overall, with GATK-gCNV showing excellent sensitivity at both the exon and gene levels (90). ExomeCNV is noted for its speed (69). DRAGEN HS-F (high-sensitivity mode with custom artefact filtering) is highlighted for clinical gene panels, achieving 100% sensitivity and strong precision (77%) after the removal of recurrent artefacts (71). Combining the results from multiple CNV detection tools can improve the overall performance. Intersecting tool calls generally increase specificity by reducing false positives, although this may come at the expense of recall, as some true positives may be missed. However, certain carefully selected tool pairs, such as Atlas-CNV with CoNVaDING, CODEX2 with CoNVaDING, and DECoN with CODEX2, have demonstrated the ability to recover all true positives at the gene level. Additionally, combining the outputs from a larger number of tools and using more than five control samples can further boost sensitivity, albeit at a significant computational cost (69, 90).

In long read sequencing (LR-seq), tool performance varies not only with the CNV type and size but also with the sequencing platform. LR-seq provides the highest breakpoint resolution among current technologies, enabling the detection of structural variations ≥50 bp, including CNVs; however, in practice, CNV calling is most robust for events ≥1 kb, with optimal performance typically observed in the ~1-100 kb range and reduced accuracy for very large or complex variants, depending on the caller and platform (91). Specifically, cuteSV, Delly, pbsv, and Sniffles2 achieved relatively high precision in CNV detection (often ≥0.7 in PacBio datasets), with even higher precision observed for deletions (approaching ~0.8), while the overall performance varied depending on the variant type and sequencing platform (91). The selection of these tools should be guided by the specific needs of the study, particularly the desired balance between sensitivity, precision, and computational efficiency. cuteSV is generally favoured for its optimal balance of precision and recall, whereas Sniffles2 offers the fastest runtime, making it suitable for rapid analysis (91). Additionally, tools such as pbsv are among the most effective for detecting small deletions in long-read datasets (81). For Nanopore sequencing data, the combination of minimap2 as the aligner with Sniffles as the CNV caller provides a robust starting point, offering balanced performance in detecting both deletions and duplications. Researchers prioritising high precision should also consider Sniffles, although it should be noted that this refers to earlier benchmarking studies and may not be directly comparable to more recent evaluations of Sniffles2 (92). Although tools such as NanoSV and NanoVar are generally reported to have lower overall accuracy and are often not recommended for routine CNV detection (91), the findings of these studies are somewhat contradictory. For instance, despite its lower precision, NanoSV has been highlighted in some evaluations for its high sensitivity, making it potentially valuable for sensitivity-driven applications (92). These discrepancies suggest that the performance of these tools may be context-dependent, and their use should be considered carefully in light of specific study objectives and dataset characteristics. To further enhance detection accuracy, it is advisable to integrate the results from multiple tools using consensus-or machine learning-based approaches. Calls supported by two or more tools have been shown to significantly improve the F1-score (81, 91, 92).

**Note S5. Challenges and considerations in copy number variation analysis on sex chromosomes**

The analysis of copy number variations (CNVs) on sex chromosomes presents distinct methodological challenges compared to autosomes, often leading to their exclusion from large-scale studies (93-96). Unlike autosomes, which are typically diploid, sex chromosomes exhibit sex-specific baseline ploidy, with males being hemizygous for the X chromosome, thereby violating the standard diploid assumptions underlying many CNV calling algorithms (97, 98). Consequently, accurate CNV detection requires specialised normalisation strategies, such as sex-specific calibration of signal intensities or explicit ploidy modelling, and in some cases, analytical simplifications, such as restricting analyses to a single sex or enforcing uniform diploid assumptions in computational models (86).

Additional complexity arises from the pseudoautosomal regions, which are shared between the X and Y chromosomes and are typically treated as diploid in both sexes. However, in individuals with sex chromosome aneuploidies (e.g., XXY), these regions may exhibit altered copy states, leading to ambiguous signal patterns and reduced genotyping accuracy (99, 100). More broadly, sex chromosome aneuploidies (e.g., XXX, XXY, XYY, or X0) can complicate both CNV detection and sample classification, often resulting in fragmented CNV calls or discrepancies between genetically inferred and reported sex, which necessitates the exclusion of ambiguous samples during quality control (QC) (99-101).

From a technical perspective, the performance of CNV detection tools on sex chromosomes is highly variable and depends on both the platform and algorithmic design. Benchmarking studies have demonstrated inconsistent performance across commonly used tools, particularly under low-coverage conditions, with some methods failing to detect CNVs or producing inflated or fragmented calls, whereas others exclude sex chromosomes entirely by default (78). In read depth-based approaches, the composition of the reference set is particularly critical, as combining male and female samples can introduce noise and reduce accuracy, whereas sex-specific reference panels and explicit ploidy settings can improve performance (86, 102).

Further methodological considerations include the need for specialised QC and analytical procedures. Sex chromosomes are commonly used for sample sex inference and validation, for example, through X-chromosome heterozygosity or coverage-based methods, and samples with discordant or ambiguous profiles are typically excluded (101, 103). In association analyses, sex is frequently included as a covariate to control for confounding effects, and in some cases, sex-stratified analyses are performed to identify sex-specific CNV associations (104-108).

**Note S6. Tools and frameworks for copy number variation association studies**

CNV association methods can be broadly divided into approaches that treat copy number variations (CNVs) as discrete events (e.g., presence/absence or deletion/duplication) and those that explicitly model copy number states across individuals (CNV genotyping). The latter class of methods, including mixture models and cohort-based approaches, is particularly important for analysing common or multiallelic CNVs in large-scale association studies (101, 109).

PLINK’s first-generation PLINK (v1.07) introduced basic CNV association commands that allowed investigators to perform permutation tests for case-control CNV burden and gene-based CNV enrichment using the same framework as single nucleotide polymorphism (SNP) genome-wide association study (GWAS) (103). Although this enables CNV analyses using a familiar tool, it has limitations. For instance, it treats CNVs as binary events (present/absent per sample) and does not model dosage beyond the presence of a CNV. Furthermore, comprehensive support for CNVs remains incomplete in second-generation PLINK (110). Nonetheless, many large consortium studies have successfully used PLINK for CNV burden tests, given its speed and ability to efficiently handle covariates and permutations. For example, the Psychiatric Genomics Consortium leveraged PLINK’s CNV functions in a study of 41,321 subjects, in which the burden of rare deletions and duplications was compared between cases and controls for disorders such as schizophrenia (98). This analysis confirmed that schizophrenia cases carried a higher load of rare disruptive CNVs than the controls. The key insight is that global burden or region-based association tests can reveal the influence of polygenic CNVs on disease. For example, an analysis of 60 complex diseases in the UK Biobank identified 73 notable associations between CNVs and diseases involving 40 conditions. This was achieved using region-level CNV GWAS approaches, revealing that the presence of a CNV in specific genomic areas increases the risk of disease or results in an earlier onset (111).

Birdsuite provides an integrated pipeline (Canary for common CNVs and Birdseye for rare CNVs) that outputs allele-specific copy number genotypes, allowing CNVs to be analysed in association models similar to SNPs (112). Studies using Birdsuite have highlighted the importance of stringent filtering to improve the reliability of calls (113). In practice, Birdsuite can identify a significant number of CNVs, including known polymorphisms, and is among the pioneering tools that facilitate combined SNP-CNV analysis. However, due to variability in CNV detection across different regions and samples, careful post-calling quality control (QC) is essential. In a study of metabolic traits within the African American HyperGEN cohort, Birdsuite software was used to call allele-specific CNVs and test their association with insulin resistance. This joint SNP-CNV genome-wide analysis identified two copy number polymorphisms in the TCRβ region that were suggestively associated with fasting insulin levels. Based on prior immunological studies reporting that certain TCR sequences can mimic insulin and its receptor, the authors proposed that variations in this region could potentially contribute to insulin resistance (114).

CNVtools pioneered a likelihood-based approach that involves fitting a mixture of Gaussian distributions to log R ratio intensity values to infer discrete copy number classes and then tests the association by comparing class frequencies between phenotypes. This approach was crucial in early CNV GWAS, where the intensity data were noisy. By modelling uncertainty, CNVtools can assign posterior probabilities to copy number genotypes (115). Its use in multiple GWAS showed that integrating CNV calling with statistical testing improved the power to detect loci that simpler approaches might miss. For instance, in the Wellcome Trust Case Control Consortium’s CNV analyses involving approximately 16,000 samples across eight prevalent diseases, CNVtools was employed alongside other methods to genotype CNV loci and assess their associations. This approach was instrumental in verifying the well-known deletion upstream of *IRGM* associated with Crohn’s disease by applying a Gaussian mixture model to the intensity data at that locus, revealing a notable difference in copy number between cases and controls (116). CNVtools underscored the need for specialised statistical frameworks for CNVs, and later tools (e.g., CNVassoc) drew on its mixture model strategy to model uncertainty.

R-GADA provides a fast, integrated pipeline from raw intensity normalisation to CNV calling and association testing. Its segmentation algorithm was noted to be orders of magnitude faster than the classic circular binary segmentation segmentation, with comparable accuracy. This speed enables the analysis of high-density SNP array data for CNVs in studies with hundreds or thousands of samples, which is an important step as CNV GWAS moves to a larger scale. The authors segmented intensity data from 270 HapMap individuals and generated a simulated case-control phenotype for association analysis. R-GADA efficiently processes approximately 3 million probes per sample in under one minute each (35). Despite its capability for association analysis, R-GADA is predominantly used for CNV detection and is rarely applied for association analysis (117-120). Another insight from R-GADA is the emphasis on population structure adjustment for CNV data analysis. The package includes principal component analysis of CNV calls to detect stratification, which can then be corrected in the association stage of the analysis. This is critical because CNV frequencies can differ by ancestry, risking confounding factors in GWAS. By implementing this, R-GADA showed improved control of false positives in the association tests. R-GADA demonstrated to the community that conducting a CNV GWAS is feasible within a single and adaptable framework. It also underscored practical considerations, such as parallelisation and the management of diverse array input formats, which were further developed (35).

CNVassoc introduced a latent class likelihood framework to explicitly account for uncertainty in copy number calls, thereby reducing bias in the association results. This tool provides flexible regression models that can incorporate covariate adjustments. A key innovation of CNVassoc is the separation of calling from association analysis; researchers can input probabilistic CNV calls from any algorithm (e.g. CGHcall, CANARY, and IMPUTE) and then perform association testing while weighing uncertainty. This design increases the statistical power and robustness of the inferences. Simulation studies have demonstrated that CNVassoc achieves markedly faster runtimes and lower convergence failure rates than CNVtools (105). Overall, CNVassoc highlighted the importance of accounting for CNV calling errors and demonstrated advantages in flexibility (e.g. different outcome types and study designs) for CNV GWAS (105, 121).

CNVRuler introduces systematic methods for defining CNV regions (CNVRs) before association. It provides three models for defining CNVRs–reciprocal overlap, simple overlap, and boundary-based segmentation–to combine individual CNVs into shared loci. After establishing CNVRs, CNVRuler facilitates various statistical analyses, including chi-square, Fisher’s exact test, logistic, and linear regression, and offers correction for population stratification. Furthermore, it features a user-friendly graphical interface and is compatible with the outputs of ten widely used CNV callers. It also includes downstream filtering capabilities based on size and signal intensity thresholds (104). In practical applications, CNVRuler has been employed in numerous studies, with significant utilisation observed in livestock genetics (122-125). In human research, CNVRuler was employed in a case-control study involving individuals of European ancestry. This tool facilitated the identification of CNVRs associated with proctitis, including one involving the *ALG1L2* gene, which was corroborated by transcriptomic analysis of whole blood tissues (126). Furthermore, CNVRuler has been extensively employed as a preprocessing method to consolidate individual CNVs into consistent CNVRs across different individuals (127-129). An important insight from the application of CNVRuler is that the definition of CNVRs significantly influences the association results. CNVRuler’s capability to refine low-density CNV clusters and consolidate overlapping calls effectively mitigates false positives arising from diffuse CNV breakpoints.

ParseCNV introduced a comprehensive pipeline for CNV association, which is characterised by rigorous QC measures. A central innovation is its dynamic handling of CNV breakpoints and lengths; rather than imposing fixed CNVR boundaries, ParseCNV flexibly merges or extends regions based on proximity and statistical evidence, ensuring that overlapping CNVs of different sizes are appropriately represented in association tests. This strategy minimises spurious signals that arise when misaligned CNVs are forced into rigid regions. Moreover, ParseCNV provides an integrated environment for multiple CNV association tests in a single software suite (107). ParseCNV has been applied in several high-impact studies. For example, in a genome-wide investigation of cardiac modifiers associated with 22q11.2 deletion syndrome, CNVs were identified using PennCNV, and association testing was performed using ParseCNV. This study led to the identification of partial microduplications of *KANSL1* as modifiers of the risk of congenital heart defects (130). In a large-scale CNV meta-analysis of five neuropsychiatric disorders, ParseCNV was used to harmonise association tests from multiple arrays, contributing to the identification and validation of a shared duplication locus at *DOCK8*/*KANK1* across schizophrenia, bipolar disorder, autism, attention deficit hyperactivity disorder (ADHD), and depression (131). Network-level analyses of neurodevelopmental CNVs have also utilised this approach, confirming the enrichment of CNVs in the *CNTN4*, 22q11.2, and 16p11.2 regions associated with ADHD and autism spectrum disorder, and mapping them to metabotropic glutamate receptor interaction networks (132). On a broader scale, the pipeline was integrated into a CNV map encompassing over 100,000 individuals, facilitating the precise estimation of CNV frequencies and enabling disease association analyses across the autoimmune, cardiometabolic, oncological, and neuropsychiatric domains (133). ParseCNV2 expanded ParseCNV’s capabilities with a redesigned backend that natively reads variant call format (VCF) calls and supports covariate adjustment and interactive QC filtering in the association step (108). In the Adolescent Brain Cognitive Development (ABCD) Study, which includes approximately 8,500 children, ParseCNV2 was used to scan genome-wide CNVRs for association with dimensional psychopathology and cognition, identifying candidate regions such as a 14q11.2 duplication linked to attention problems and showing that higher genome-wide CNV risk scores, which are based on gene intolerance and dosage sensitivity, correlate with poorer working memory, processing speed, and fluid intelligence (134). ParseCNV2 was also applied to biobank-scale datasets. It was benchmarked on the UK Biobank with approximately 450,000 participants to efficiently scan for CNV associations (108). The versatility of ParseCNV/2 is highlighted by its application to both hypothesis-driven and exploratory CNV research, demonstrating its reliability across various genotyping platforms and its contribution to the identification of novel genotype-phenotype associations.

CNVtest introduced a one-pass scanning approach that avoids preset CNV loci. It uses a variable-length interval scanning approach optimised to detect CNV trait associations directly from intensity data. This unified approach means that CNV detection and association are performed simultaneously, thereby improving the computational efficiency. An important advantage of CNVtest is its ability to manage misaligned breakpoints, enabling the detection of associations even when the CNVs of the carriers do not exhibit identical boundaries. The framework of CNVtest can be extended to various trait models through the application of generalised linear models and is capable of accommodating read depth data. Although primarily demonstrated through simulations, the authors applied the method to empirical data to identify CNVs associated with population differentiation. This application demonstrated the efficacy of this method in controlling genome-wide type I error while successfully detecting trait-associated CNVs (135).

Wham provides a unified framework that simultaneously calls structural variations (SVs), including CNVs, and evaluates their case-control association. It effectively treats SVs as alleles and uses a likelihood ratio test within the variant-calling step to check for phenotypic associations. It integrates multiple breakpoint signals (split reads, mate-pair discordance, soft-clipping, and alternative alignments) to detect SVs, which improves the ability to identify these associations. Furthermore, Wham’s capability to analyse pooled sequencing data while simultaneously conducting association testing represents a notable advancement in this field. The authors demonstrated Wham on multiple datasets. In one example, they analysed domestic pigeon populations with distinct physical traits and showed that Wham could identify SVs associated with these traits (136). Numerous studies have demonstrated the application of Wham, either independently or as part of an integrated pipeline, for the identification of CNVs and other SVs associated with various phenotypes and diseases (137-140). However, Wham was primarily developed and validated as an SV/CNV discovery tool; its built-in association testing has not been extensively benchmarked against established GWAS frameworks.

The primary contribution of CoNVaQ is to enhance accessibility and simplify the workflow of CNV association study. The tool offers a user-friendly R interface and an intuitive web interface (unavailable at the time of writing), enabling users to upload the CNV segments for both the cohorts. It automatically identifies CNVRs and conducts association testing, employing either a simple Fisher’s exact test for CNV occurrence or a custom query-based model to evaluate user-specified CNV frequency patterns. In practical applications, CoNVaQ has been commended for reducing accessibility barriers for clinicians. For instance, a geneticist lacking programming expertise can upload data to identify candidate CNVRs associated with specific traits of interest. Another aspect is the integrated enrichment analysis. After identifying significant CNVRs, CoNVaQ’s report highlighted any gene ontology terms or pathways that were overrepresented among the genes in these regions. To demonstrate its potential, the developers utilised CoNVaQ to analyse CNVs between two groups of patients with penile cancer: those with human papillomavirus (HPV)-positive and HPV-negative status. CoNVaQ identified several genomic regions where CNVs were more prevalent in one group than in the other, indicating distinct molecular causes for these differences. Although this instance pertains to cancer research, the tool is equally suitable for non-cancer phenotypes, as it only requires two sets of CNV data (such as cases versus controls or any two groups) (141).

CNVRanger provides a structured downstream analysis pipeline for CNV analysis. It employs multiple methodologies, including the CNVRuler method, reciprocal overlap, and GISTIC, to consolidate individual CNV calls into recurrent CNVRs. CNVRanger evaluates the overlap of CNVRs with functional genomic regions by utilising permutation-based enrichment tests (via regioneR) and conducting association tests with either quantitative traits or gene expression (CNV-expression quantitative trait loci (eQTL) analysis). A significant attribute of CNVRanger in the context of SNP-chip-derived CNV calls is its capability for probe-level association testing, which can enhance the statistical power for detecting common CNVs (142). The application of this method across a range of organisms, including Populus trichocarpa, Holstein cattle, various pig and chicken breeds, and human health studies, highlights its extensive utility (120, 143-148).

SNATCNV is a tool tailored to detect CNV associations at single-nucleotide resolution. Essentially, it evaluates the phenotype association for each genomic position by considering whether a CNV covers the position of each individual. This high-resolution methodology has been demonstrated to enhance the detection of small or overlapping CNVs, as it obviates the need to predefine the entire CNV segment as the unit of analysis. In comparative analyses, SNATCNV demonstrated superior performance in accurately identifying narrow CNV hotspots within genes. An additional insight is the significance of interpreting the gene sets. This underscores the importance of understanding the concentration of CNVs in specific gene categories, beyond determining whether they are associated with a particular disorder. The authors employed SNATCNV to analyse extensive psychiatric CNV datasets. In the context of autism, they examined CNV data from 19,663 cases and 6,479 controls, successfully identifying well-established pathogenic loci, such as the 16p11.2 deletion, and discovered additional recurrent CNVRs. Subsequent functional analyses of these regions assessed their gene content in relation to brain biology. In a separate schizophrenia dataset consisting of 27,034 cases and 25,448 controls, SNATCNV identified CNVs enriched in cases, once again implicating genes pertinent to neuropsychiatric diseases (149).

PeakCNV employs a multi-feature ranking algorithm to prioritise CNVRs for association studies. Instead of indiscriminately testing each CNVR, the method constructs CNV maps for both the case and control groups, clusters overlapping CNVRs, and ranks these clusters using the independence ranking score. This score integrates multiple features, including frequency differences and independence from other clusters. This methodology has been demonstrated to decrease the incidence of false positives and concentrate attention on the most probable pathogenic CNVs. An important insight is that PeakCNV addresses the dependency among CNVs by considering multiple overlapping CNVs within the same region as a single signal. This approach was employed to prevent redundant testing. Breakpoint heterogeneity was addressed by merging highly similar CNVRs and selecting peak regions within clusters. The tool confers both visual and interpretative benefits by producing a ranked list of CNVRs accompanied by scores, enabling researchers to efficiently identify which CNVs merit further biological exploration. In their benchmarks, the PeakCNV team compared its output to other tools using two case studies: a prostate cancer cohort (194 cases, 2,392 controls) and a much larger neurodevelopmental disorder dataset (19,642 cases with conditions such as autism or developmental delay and 6,451 controls). In the analysis of prostate cancer, PeakCNV identified fewer candidate CNVRs, and those identified were, on average, shorter than those detected by other methods. This is advantageous because it indicates that the tool focuses on the minimal critical regions. Notably, the CNVRs identified by PeakCNV encompassed a larger proportion of cases, indicating that a greater number of patients possessed these CNVs than the regions identified through conventional methods. In the dataset pertaining to neurodevelopmental disorders, the top-ranked CNVRs identified by PeakCNV demonstrated significant biological relevance. These CNVRs exhibited a greater overlap with genes characterised by brain-enriched expression and established neurological functions than the CNVs identified by alternative tools (150).

CNest has developed a comprehensive framework for CNV calling and association testing tailored to next generation sequencing (NGS) data. This framework is specifically designed to facilitate large-scale genome-wide analyses in the discovery mode, without the need for prior specification of loci. It estimates copy numbers from sequencing read depth with robust normalisation and bias correction, enabling accurate *de novo* CNV discovery and GWAS in a unified workflow. A key insight from CNest’s analysis of UK Biobank data is that certain CNV associations are independent of SNP signals. CNest categorises CNV associations based on their detectability through SNP-based GWAS, thereby identifying genuinely novel loci driven by CNVs. This methodology was applied to approximately 200,000 exome-sequenced participants from the UK Biobank, encompassing 78 traits, and resulted in the identification of 862 CNV-trait associations across a diverse range of phenotypes (101).

Most of the tools discussed were developed for SNP arrays or short-read sequencing data and do not support long-read inputs. CNVs from long-read sequencing (LR-seq) are often defined more precisely in terms of breakpoints and allelic complexity, which poses difficulties in aligning or comparing them with array-derived CNVs (42). There is a clear need for new or adapted tools that can incorporate long-read-derived CNVs into population-based association models, while accounting for differences in detection sensitivity and variant boundaries. To enable robust association testing of CNVs identified from LR-seq data, Beyter et al. (151) implemented a comprehensive computational framework that integrated SV/CNV discovery, genotyping, phasing, and imputation into population-scale association models. After discovering CNVs using long-read data with tools such as Sniffles and refining breakpoints with SViper, the authors applied LRcaller, a purpose-built genotyper for Oxford Nanopore reads, to produce high-resolution genotypes for all the individuals. These genotypes were then phased using long-range haplotypes and imputed into a much larger SNP-chip-typed cohort of 166,281 individuals using established methods. To ensure high accuracy in downstream association, only variants that passed stringent imputation quality metrics were retained for the analysis. For association analysis, they employed linear mixed models (BOLT-LMM) that account for relatedness and population structure and used linkage disequilibrium (LD) score regression to correct for inflation. Importantly, they also leveraged LD with known GWAS variants to annotate likely causal CNVs and performed direct association tests for high-impact CNVs affecting phenotypes, such as low-density lipoprotein (LDL) cholesterol and height. This workflow demonstrates how long-read-derived CNVs can be systematically integrated into association studies using custom computational pipelines and imputation-based methods, despite their complex allelic structures and breakpoint variability.

**References**

1.	Gordeeva V, Sharova E, Arapidi G. Progress in Methods for Copy Number Variation Profiling. Int J Mol Sci. 2022;23(4).

2.	Ho SS, Urban AE, Mills RE. Structural variation in the sequencing era. Nat Rev Genet. 2020;21(3):171-89.

3.	Iafrate AJ, Feuk L, Rivera MN, Listewnik ML, Donahoe PK, Qi Y, et al. Detection of large-scale variation in the human genome. Nat Genet. 2004;36(9):949-51.

4.	Sebat J, Lakshmi B, Troge J, Alexander J, Young J, Lundin P, et al. Large-scale copy number polymorphism in the human genome. Science. 2004;305(5683):525-8.

5.	Miller DT, Adam MP, Aradhya S, Biesecker LG, Brothman AR, Carter NP, et al. Consensus statement: chromosomal microarray is a first-tier clinical diagnostic test for individuals with developmental disabilities or congenital anomalies. Am J Hum Genet. 2010;86(5):749-64.

6.	Schaefer GB, Mendelsohn NJ. Clinical genetics evaluation in identifying the etiology of autism spectrum disorders: 2013 guideline revisions. Genet Med. 2013;15(5):399-407.

7.	Collins RL, Talkowski ME. Diversity and consequences of structural variation in the human genome. Nat Rev Genet. 2025.

8.	Zhao M, Wang Q, Wang Q, Jia P, Zhao Z. Computational tools for copy number variation (CNV) detection using next-generation sequencing data: features and perspectives. BMC Bioinformatics. 2013;14(11):S1.

9.	Mahmoud M, Gobet N, Cruz-Dávalos DI, Mounier N, Dessimoz C, Sedlazeck FJ. Structural variant calling: the long and the short of it. Genome Biol. 2019;20(1):246.

10.	Comprehensive gene panels provide advantages over clinical exome sequencing for Mendelian diseases. Genome Biol. 2015;16(1):134.

11.	Klein CJ, Foroud TM. Neurology Individualized Medicine: When to Use Next-Generation Sequencing Panels. Mayo Clin Proc. 2017;92(2):292-305.

12.	Merker JD, Wenger AM, Sneddon T, Grove M, Zappala Z, Fresard L, et al. Long-read genome sequencing identifies causal structural variation in a Mendelian disease. Genet Med. 2018;20(1):159-63.

13.	Song JHT, Lowe CB, Kingsley DM. Characterization of a Human-Specific Tandem Repeat Associated with Bipolar Disorder and Schizophrenia. Am J Hum Genet. 2018;103(3):421-30.

14.	Ashley EA. Towards precision medicine. Nat Rev Genet. 2016;17(9):507-22.

15.	Goldfeder RL, Priest JR, Zook JM, Grove ME, Waggott D, Wheeler MT, et al. Medical implications of technical accuracy in genome sequencing. Genome Med. 2016;8(1):24.

16.	Chaisson MJ, Huddleston J, Dennis MY, Sudmant PH, Malig M, Hormozdiari F, et al. Resolving the complexity of the human genome using single-molecule sequencing. Nature. 2015;517(7536):608-11.

17.	Huddleston J, Chaisson MJP, Steinberg KM, Warren W, Hoekzema K, Gordon D, et al. Discovery and genotyping of structural variation from long-read haploid genome sequence data. Genome Res. 2017;27(5):677-85.

18.	Goodwin S, McPherson JD, McCombie WR. Coming of age: ten years of next-generation sequencing technologies. Nat Rev Genet. 2016;17(6):333-51.

19.	Gao R, Bai S, Henderson YC, Lin Y, Schalck A, Yan Y, et al. Delineating copy number and clonal substructure in human tumors from single-cell transcriptomes. Nat Biotechnol. 2021;39(5):599-608.

20.	Fan J, Lee HO, Lee S, Ryu DE, Lee S, Xue C, et al. Linking transcriptional and genetic tumor heterogeneity through allele analysis of single-cell RNA-seq data. Genome Res. 2018;28(8):1217-27.

21.	Müller S, Cho A, Liu SJ, Lim DA, Diaz A. CONICS integrates scRNA-seq with DNA sequencing to map gene expression to tumor sub-clones. Bioinformatics. 2018;34(18):3217-9.

22.	Harmanci AS, Harmanci A, Zhou X. CaSpER identifies and visualizes CNV events by integrative analysis of single-cell or bulk RNA-sequencing data. Nature Communications. 2020;11.

23.	De Falco A, Caruso F, Su XD, Iavarone A, Ceccarelli M. A variational algorithm to detect the clonal copy number substructure of tumors from scRNA-seq data. Nat Commun. 2023;14(1):1074.

24.	Gao T, Soldatov R, Sarkar H, Kurkiewicz A, Biederstedt E, Loh PR, et al. Haplotype-aware analysis of somatic copy number variations from single-cell transcriptomes. Nat Biotechnol. 2023;41(3):417-26.

25.	Roy S, Motsinger Reif A. Evaluation of calling algorithms for array-CGH. Front Genet. 2013;4:217.

26.	Killick R, Fearnhead P, Eckley IA. Optimal detection of changepoints with a linear computational cost. Journal of the American Statistical Association. 2012;107(500):1590-8.

27.	Olshen AB, Venkatraman ES, Lucito R, Wigler M. Circular binary segmentation for the analysis of array-based DNA copy number data. Biostatistics. 2004;5(4):557-72.

28.	Darvishi K. Application of Nexus copy number software for CNV detection and analysis. Curr Protoc Hum Genet. 2010;Chapter 4:Unit 4.14.1-28.

29.	Niu YS, Zhang H. THE SCREENING AND RANKING ALGORITHM TO DETECT DNA COPY NUMBER VARIATIONS. Ann Appl Stat. 2012;6(3):1306-26.

30.	Jeng XJ, Cai TT, Li H. Optimal Sparse Segment Identification with Application in Copy Number Variation Analysis. J Am Stat Assoc. 2010;105(491):1156-66.

31.	Zhang Z, Lange K, Ophoff R, Sabatti C. RECONSTRUCTING DNA COPY NUMBER BY PENALIZED ESTIMATION AND IMPUTATION. Ann Appl Stat. 2010;4(4):1749-73.

32.	Tibshirani R, Wang P. Spatial smoothing and hot spot detection for CGH data using the fused lasso. Biostatistics. 2008;9(1):18-29.

33.	Wang K, Li M, Hadley D, Liu R, Glessner J, Grant SF, et al. PennCNV: an integrated hidden Markov model designed for high-resolution copy number variation detection in whole-genome SNP genotyping data. Genome Res. 2007;17(11):1665-74.

34.	Colella S, Yau C, Taylor JM, Mirza G, Butler H, Clouston P, et al. QuantiSNP: an Objective Bayes Hidden-Markov Model to detect and accurately map copy number variation using SNP genotyping data. Nucleic Acids Res. 2007;35(6):2013-25.

35.	Pique-Regi R, Cáceres A, González JR. R-Gada: a fast and flexible pipeline for copy number analysis in association studies. BMC Bioinformatics. 2010;11:380.

36.	Zhang Z, Cheng H, Hong X, Di Narzo AF, Franzen O, Peng S, et al. EnsembleCNV: an ensemble machine learning algorithm to identify and genotype copy number variation using SNP array data. Nucleic Acids Res. 2019;47(7):e39.

37.	Pinto D, Pagnamenta AT, Klei L, Anney R, Merico D, Regan R, et al. Functional impact of global rare copy number variation in autism spectrum disorders. Nature. 2010;466(7304):368-72.

38.	Pirooznia M, Goes FS, Zandi PP. Whole-genome CNV analysis: advances in computational approaches. Frontiers in Genetics. 2015;6.

39.	Magi A, Tattini L, Pippucci T, Torricelli F, Benelli M. Read count approach for DNA copy number variants detection. Bioinformatics. 2012;28(4):470-8.

40.	Ahsan MU, Liu Q, Perdomo JE, Fang L, Wang K. A survey of algorithms for the detection of genomic structural variants from long-read sequencing data. Nat Methods. 2023;20(8):1143-58.

41.	Jiang T, Liu Y, Jiang Y, Li J, Gao Y, Cui Z, et al. Long-read-based human genomic structural variation detection with cuteSV. Genome Biology. 2020;21(1):189.

42.	Sedlazeck FJ, Rescheneder P, Smolka M, Fang H, Nattestad M, von Haeseler A, et al. Accurate detection of complex structural variations using single-molecule sequencing. Nature Methods. 2018;15(6):461-8.

43.	Smolka M, Paulin LF, Grochowski CM, Horner DW, Mahmoud M, Behera S, et al. Detection of mosaic and population-level structural variants with Sniffles2. Nature Biotechnology. 2024;42(10):1571-80.

44.	English AC, Salerno WJ, Reid JG. PBHoney: identifying genomic variants via long-read discordance and interrupted mapping. BMC Bioinformatics. 2014;15(1):180.

45.	Cretu Stancu M, van Roosmalen MJ, Renkens I, Nieboer MM, Middelkamp S, de Ligt J, et al. Mapping and phasing of structural variation in patient genomes using nanopore sequencing. Nature Communications. 2017;8(1):1326.

46.	Tham CY, Tirado-Magallanes R, Goh Y, Fullwood MJ, Koh BTH, Wang W, et al. NanoVar: accurate characterization of patients’ genomic structural variants using low-depth nanopore sequencing. Genome Biology. 2020;21(1):56.

47.	Gong L, Wong CH, Cheng WC, Tjong H, Menghi F, Ngan CY, et al. Picky comprehensively detects high-resolution structural variants in nanopore long reads. Nat Methods. 2018;15(6):455-60.

48.	Luo J, Ding H, Shen J, Zhai H, Wu Z, Yan C, et al. BreakNet: detecting deletions using long reads and a deep learning approach. BMC Bioinformatics. 2021;22(1):577.

49.	Ding H, Luo J. MAMnet: detecting and genotyping deletions and insertions based on long reads and a deep learning approach. Brief Bioinform. 2022;23(5).

50.	Lin J, Wang S, Audano PA, Meng D, Flores JI, Kosters W, et al. SVision: a deep learning approach to resolve complex structural variants. Nat Methods. 2022;19(10):1230-3.

51.	Goel M, Sun H, Jiao W-B, Schneeberger K. SyRI: finding genomic rearrangements and local sequence differences from whole-genome assemblies. Genome Biology. 2019;20(1):277.

52.	Wenger AM, Peluso P, Rowell WJ, Chang PC, Hall RJ, Concepcion GT, et al. Accurate circular consensus long-read sequencing improves variant detection and assembly of a human genome. Nat Biotechnol. 2019;37(10):1155-62.

53.	Heller D, Vingron M. SVIM-asm: structural variant detection from haploid and diploid genome assemblies. Bioinformatics. 2020;36(22-23):5519-21.

54.	Zhang Y, Liu W, Duan J. On the core segmentation algorithms of copy number variation detection tools. Briefings in Bioinformatics. 2024;25(2).

55.	Miller CA, Hampton O, Coarfa C, Milosavljevic A. ReadDepth: a parallel R package for detecting copy number alterations from short sequencing reads. PLoS One. 2011;6(1):e16327.

56.	Fromer M, Moran JL, Chambert K, Banks E, Bergen SE, Ruderfer DM, et al. Discovery and statistical genotyping of copy-number variation from whole-exome sequencing depth. Am J Hum Genet. 2012;91(4):597-607.

57.	Magi A, Benelli M, Marseglia G, Nannetti G, Scordo MR, Torricelli F. A shifting level model algorithm that identifies aberrations in array-CGH data. Biostatistics. 2010;11(2):265-80.

58.	Magi A, Pippucci T, Sidore C. XCAVATOR: accurate detection and genotyping of copy number variants from second and third generation whole-genome sequencing experiments. BMC Genomics. 2017;18(1):747.

59.	Magi A, Benelli M, Yoon S, Roviello F, Torricelli F. Detecting common copy number variants in high-throughput sequencing data by using JointSLM algorithm. Nucleic Acids Res. 2011;39(10):e65.

60.	Magi A, Tattini L, Cifola I, D'Aurizio R, Benelli M, Mangano E, et al. EXCAVATOR: detecting copy number variants from whole-exome sequencing data. Genome Biol. 2013;14(10):R120.

61.	Wang LY, Abyzov A, Korbel JO, Snyder M, Gerstein M. MSB: a mean-shift-based approach for the analysis of structural variation in the genome. Genome Res. 2009;19(1):106-17.

62.	Abyzov A, Urban AE, Snyder M, Gerstein M. CNVnator: an approach to discover, genotype, and characterize typical and atypical CNVs from family and population genome sequencing. Genome Res. 2011;21(6):974-84.

63.	Mumford D, Shah J. Optimal approximations by piecewise smooth functions and associated variational problems. Communications on Pure and Applied Mathematics. 1989;42(5):577-685.

64.	Anjum S, Morganella S, D'Angelo F, Iavarone A, Ceccarelli M. VEGAWES: variational segmentation on whole exome sequencing for copy number detection. BMC Bioinformatics. 2015;16:315.

65.	Morganella S, Cerulo L, Viglietto G, Ceccarelli M. VEGA: variational segmentation for copy number detection. Bioinformatics. 2010;26(24):3020-7.

66.	Vardhanabhuti S, Jeng XJ, Wu Y, Li H. Parametric modeling of whole-genome sequencing data for CNV identification. Biostatistics. 2014;15(3):427-41.

67.	Yuan X, Yu J, Xi J, Yang L, Shang J, Li Z, et al. CNV_IFTV: An Isolation Forest and Total Variation-Based Detection of CNVs from Short-Read Sequencing Data. IEEE/ACM Trans Comput Biol Bioinform. 2021;18(2):539-49.

68.	Cabello-Aguilar S, Vendrell JA, Van Goethem C, Brousse M, Gozé C, Frantz L, et al. ifCNV: A novel isolation-forest-based package to detect copy-number variations from various targeted NGS datasets. Mol Ther Nucleic Acids. 2022;30:174-83.

69.	Roca I, González-Castro L, Fernández H, Couce ML, Fernández-Marmiesse A. Free-access copy-number variant detection tools for targeted next-generation sequencing data. Mutat Res Rev Mutat Res. 2019;779:114-25.

70.	Carter NP. Methods and strategies for analyzing copy number variation using DNA microarrays. Nat Genet. 2007;39(7 Suppl):S16-21.

71.	De La Vega FM, Irvine SA, Anur P, Potts K, Kraft L, Torres R, et al. Benchmarking of germline copy number variant callers from whole genome sequencing data for clinical applications. Bioinform Adv. 2025;5(1):vbaf071.

72.	Silva C, Ferrão J, Marques B, Pedro S, Correia H, Valente A, et al. Comparative analysis of hybrid-SNP microarray and nanopore sequencing for detection of large-sized copy number variants in the human genome. Mol Cytogenet. 2025;18(1):18.

73.	Nutsua ME, Fischer A, Nebel A, Hofmann S, Schreiber S, Krawczak M, et al. Family-Based Benchmarking of Copy Number Variation Detection Software. PLoS One. 2015;10(7):e0133465.

74.	van Baardwijk MN, Heijnen L, Zhao H, Baudis M, Stubbs AP. A systematic benchmark of copy number variation detection tools for high density SNP genotyping arrays. Genomics. 2024;116(6):110962.

75.	Whitford W, Lehnert K, Snell RG, Jacobsen JC. Evaluation of the performance of copy number variant prediction tools for the detection of deletions from whole genome sequencing data. J Biomed Inform. 2019;94:103174.

76.	Zhang L, Bai W, Yuan N, Du Z. Comprehensively benchmarking applications for detecting copy number variation. PLoS Comput Biol. 2019;15(5):e1007069.

77.	Trost B, Walker S, Wang Z, Thiruvahindrapuram B, MacDonald JR, Sung WWL, et al. A Comprehensive Workflow for Read Depth-Based Identification of Copy-Number Variation from Whole-Genome Sequence Data. Am J Hum Genet. 2018;102(1):142-55.

78.	Smolander J, Khan S, Singaravelu K, Kauko L, Lund RJ, Laiho A, et al. Evaluation of tools for identifying large copy number variations from ultra-low-coverage whole-genome sequencing data. BMC Genomics. 2021;22(1):357.

79.	Behera S, Catreux S, Rossi M, Truong S, Huang Z, Ruehle M, et al. Comprehensive genome analysis and variant detection at scale using DRAGEN. Nature Biotechnology. 2025;43(7):1177-91.

80.	Zhao S, Xu D, Cai J, Shen Q, He M, Pan X, et al. Benchmarking strategies for CNV calling from whole genome bisulfite data in humans. Comput Struct Biotechnol J. 2025;27:912-9.

81.	Kosugi S, Momozawa Y, Liu X, Terao C, Kubo M, Kamatani Y. Comprehensive evaluation of structural variation detection algorithms for whole genome sequencing. Genome Biol. 2019;20(1):117.

82.	Coutelier M, Holtgrewe M, Jäger M, Flöttman R, Mensah MA, Spielmann M, et al. Combining callers improves the detection of copy number variants from whole-genome sequencing. Eur J Hum Genet. 2022;30(2):178-86.

83.	Gabrielaite M, Torp MH, Rasmussen MS, Andreu-Sánchez S, Vieira FG, Pedersen CB, et al. A Comparison of Tools for Copy-Number Variation Detection in Germline Whole Exome and Whole Genome Sequencing Data. Cancers (Basel). 2021;13(24).

84.	Tan R, Wang Y, Kleinstein SE, Liu Y, Zhu X, Guo H, et al. An evaluation of copy number variation detection tools from whole-exome sequencing data. Hum Mutat. 2014;35(7):899-907.

85.	Gordeeva V, Sharova E, Babalyan K, Sultanov R, Govorun VM, Arapidi G. Benchmarking germline CNV calling tools from exome sequencing data. Scientific Reports. 2021;11(1):14416.

86.	Samarakoon PS, Sorte HS, Kristiansen BE, Skodje T, Sheng Y, Tjønnfjord GE, et al. Identification of copy number variants from exome sequence data. BMC Genomics. 2014;15(1):661.

87.	Moreno-Cabrera JM, Del Valle J, Castellanos E, Feliubadaló L, Pineda M, Brunet J, et al. Evaluation of CNV detection tools for NGS panel data in genetic diagnostics. Eur J Hum Genet. 2020;28(12):1645-55.

88.	Royer-Bertrand B, Cisarova K, Niel-Butschi F, Mittaz-Crettol L, Fodstad H, Superti-Furga A. CNV Detection from Exome Sequencing Data in Routine Diagnostics of Rare Genetic Disorders: Opportunities and Limitations. Genes (Basel). 2021;12(9).

89.	Lepkes L, Kayali M, Blümcke B, Weber J, Suszynska M, Schmidt S, et al. Performance of In Silico Prediction Tools for the Detection of Germline Copy Number Variations in Cancer Predisposition Genes in 4208 Female Index Patients with Familial Breast and Ovarian Cancer. Cancers (Basel). 2021;13(1).

90.	Munté E, Roca C, Del Valle J, Feliubadaló L, Pineda M, Gel B, et al. Detection of germline CNVs from gene panel data: benchmarking the state of the art. Brief Bioinform. 2024;26(1).

91.	Yuan N, Jia P. Comprehensive assessment of long-read sequencing platforms and calling algorithms for detection of copy number variation. Brief Bioinform. 2024;25(5).

92.	Zhou A, Lin T, Xing J. Evaluating nanopore sequencing data processing pipelines for structural variation identification. Genome Biol. 2019;20(1):237.

93.	Lo Faro V, Ten Brink JB, Snieder H, Jansonius NM, Bergen AA. Genome-wide CNV investigation suggests a role for cadherin, Wnt, and p53 pathways in primary open-angle glaucoma. BMC Genomics. 2021;22(1):590.

94.	Han J, Walters JT, Kirov G, Pocklington A, Escott-Price V, Owen MJ, et al. Gender differences in CNV burden do not confound schizophrenia CNV associations. Sci Rep. 2016;6:25986.

95.	Kikuchi M, Kobayashi K, Nishida N, Sawai H, Sugiyama M, Mizokami M, et al. Genome-wide copy number variation analysis of hepatitis B infection in a Japanese population. Hum Genome Var. 2021;8(1):22.

96.	Rodríguez-López J, Flórez G, Blanco V, Pereiro C, Fernández JM, Fariñas E, et al. Genome wide analysis of rare copy number variations in alcohol abuse or dependence. J Psychiatr Res. 2018;103:212-8.

97.	Auwerx C, Lepamets M, Sadler MC, Patxot M, Stojanov M, Baud D, et al. The individual and global impact of copy-number variants on complex human traits. Am J Hum Genet. 2022;109(4):647-68.

98.	Marshall CR, Howrigan DP, Merico D, Thiruvahindrapuram B, Wu W, Greer DS, et al. Contribution of copy number variants to schizophrenia from a genome-wide study of 41,321 subjects. Nat Genet. 2017;49(1):27-35.

99.	Bycroft C, Freeman C, Petkova D, Band G, Elliott LT, Sharp K, et al. The UK Biobank resource with deep phenotyping and genomic data. Nature. 2018;562(7726):203-9.

100.	Vevera J, Zarrei M, Hartmannová H, Jedličková I, Mušálková D, Přistoupilová A, et al. Rare copy number variation in extremely impulsively violent males. Genes Brain Behav. 2019;18(6):e12536.

101.	Fitzgerald T, Birney E. CNest: A novel copy number association discovery method uncovers 862 new associations from 200,629 whole-exome sequence datasets in the UK Biobank. Cell Genomics. 2022;2(8):100167.

102.	Babadi M, Fu JM, Lee SK, Smirnov AN, Gauthier LD, Walker M, et al. GATK-gCNV enables the discovery of rare copy number variants from exome sequencing data. Nat Genet. 2023;55(9):1589-97.

103.	Purcell S, Neale B, Todd-Brown K, Thomas L, Ferreira MA, Bender D, et al. PLINK: a tool set for whole-genome association and population-based linkage analyses. Am J Hum Genet. 2007;81(3):559-75.

104.	Kim JH, Hu HJ, Yim SH, Bae JS, Kim SY, Chung YJ. CNVRuler: a copy number variation-based case-control association analysis tool. Bioinformatics. 2012;28(13):1790-2.

105.	Subirana I, Diaz-Uriarte R, Lucas G, Gonzalez JR. CNVassoc: Association analysis of CNV data using R. BMC Med Genomics. 2011;4:47.

106.	Wittig M, Helbig I, Schreiber S, Franke A. CNVineta: a data mining tool for large case-control copy number variation datasets. Bioinformatics. 2010;26(17):2208-9.

107.	Glessner JT, Li J, Hakonarson H. ParseCNV integrative copy number variation association software with quality tracking. Nucleic Acids Res. 2013;41(5):e64.

108.	Glessner JT, Li J, Liu Y, Khan M, Chang X, Sleiman PMA, et al. ParseCNV2: efficient sequencing tool for copy number variation genome-wide association studies. European Journal of Human Genetics. 2023;31(3):304-12.

109.	Handsaker RE, Korn JM, Nemesh J, McCarroll SA. Discovery and genotyping of genome structural polymorphism by sequencing on a population scale. Nat Genet. 2011;43(3):269-76.

110.	Chang CC, Chow CC, Tellier LC, Vattikuti S, Purcell SM, Lee JJ. Second-generation PLINK: rising to the challenge of larger and richer datasets. Gigascience. 2015;4:7.

111.	Auwerx C, Jõeloo M, Sadler MC, Tesio N, Ojavee S, Clark CJ, et al. Rare copy-number variants as modulators of common disease susceptibility. Genome Med. 2024;16(1):5.

112.	Korn JM, Kuruvilla FG, McCarroll SA, Wysoker A, Nemesh J, Cawley S, et al. Integrated genotype calling and association analysis of SNPs, common copy number polymorphisms and rare CNVs. Nat Genet. 2008;40(10):1253-60.

113.	Zhang X, Du R, Li S, Zhang F, Jin L, Wang H. Evaluation of copy number variation detection for a SNP array platform. BMC Bioinformatics. 2014;15(1):50.

114.	Irvin MR, Wineinger NE, Rice TK, Pajewski NM, Kabagambe EK, Gu CC, et al. Genome-Wide Detection of Allele Specific Copy Number Variation Associated with Insulin Resistance in African Americans from the HyperGEN Study. PLOS ONE. 2011;6(8):e24052.

115.	Barnes C, Plagnol V, Fitzgerald T, Redon R, Marchini J, Clayton D, et al. A robust statistical method for case-control association testing with copy number variation. Nat Genet. 2008;40(10):1245-52.

116.	Craddock N, Hurles ME, Cardin N, Pearson RD, Plagnol V, Robson S, et al. Genome-wide association study of CNVs in 16,000 cases of eight common diseases and 3,000 shared controls. Nature. 2010;464(7289):713-20.

117.	Kasak L, Rull K, Sõber S, Laan M. Copy number variation profile in the placental and parental genomes of recurrent pregnancy loss families. Sci Rep. 2017;7:45327.

118.	Rambo-Martin BL, Mulle JG, Cutler DJ, Bean LJH, Rosser TC, Dooley KJ, et al. Analysis of Copy Number Variants on Chromosome 21 in Down Syndrome-Associated Congenital Heart Defects. G3 (Bethesda). 2018;8(1):105-11.

119.	Han N, Oh JM, Kim IW. Combination of Genome-Wide Polymorphisms and Copy Number Variations of Pharmacogenes in Koreans. J Pers Med. 2021;11(1).

120.	Getmantseva L, Kolosova M, Fede K, Korobeinikova A, Kolosov A, Romanets E, et al. Finding Predictors of Leg Defects in Pigs Using CNV-GWAS. Genes (Basel). 2023;14(11).

121.	Balagué-Dobón L, Cáceres A, González JR. Fully exploiting SNP arrays: a systematic review on the tools to extract underlying genomic structure. Brief Bioinform. 2022;23(2).

122.	de Almeida Santana MH, Junior GA, Cesar AS, Freua MC, da Costa Gomes R, da Luz ESS, et al. Copy number variations and genome-wide associations reveal putative genes and metabolic pathways involved with the feed conversion ratio in beef cattle. J Appl Genet. 2016;57(4):495-504.

123.	Silva VH, Regitano LC, Geistlinger L, Pértille F, Giachetto PF, Brassaloti RA, et al. Genome-Wide Detection of CNVs and Their Association with Meat Tenderness in Nelore Cattle. PLoS One. 2016;11(6):e0157711.

124.	de Lemos MVA, Peripolli E, Berton MP, Feitosa FLB, Olivieri BF, Stafuzza NB, et al. Association study between copy number variation and beef fatty acid profile of Nellore cattle. J Appl Genet. 2018;59(2):203-23.

125.	Sasaki S, Watanabe T, Ibi T, Hasegawa K, Sakamoto Y, Moriwaki S, et al. Identification of deleterious recessive haplotypes and candidate deleterious recessive mutations in Japanese Black cattle. Sci Rep. 2021;11(1):6687.

126.	Pathak GA, Polimanti R, Silzer TK, Wendt FR, Chakraborty R, Phillips NR. Genetically-regulated transcriptomics & copy number variation of proctitis points to altered mitochondrial and DNA repair mechanisms in individuals of European ancestry. BMC Cancer. 2020;20(1):954.

127.	Zhang M, Li Q, Wang KL, Dong Y, Mu YT, Cao YM, et al. Lipolysis and gestational diabetes mellitus onset: a case-cohort genome-wide association study in Chinese. J Transl Med. 2023;21(1):47.

128.	Wang K, Cadzow M, Bixley M, Leask MP, Merriman ME, Yang Q, et al. A Polynesian-specific copy number variant encompassing the MICA gene associates with gout. Hum Mol Genet. 2022;31(21):3757-68.

129.	Oliveira P, Costa GNO, Damasceno AKA, Hartwig FP, Barbosa GCG, Figueiredo CA, et al. Genome-wide burden and association analyses implicate copy number variations in asthma risk among children and young adults from Latin America. Sci Rep. 2018;8(1):14475.

130.	León LE, Benavides F, Espinoza K, Vial C, Alvarez P, Palomares M, et al. Partial microduplication in the histone acetyltransferase complex member KANSL1 is associated with congenital heart defects in 22q11.2 microdeletion syndrome patients. Sci Rep. 2017;7(1):1795.

131.	Glessner JT, Li J, Wang D, March M, Lima L, Desai A, et al. Copy number variation meta-analysis reveals a novel duplication at 9p24 associated with multiple neurodevelopmental disorders. Genome Med. 2017;9(1):106.

132.	Glessner JT, Khan ME, Chang X, Liu Y, Otieno FG, Lemma M, et al. Rare recurrent copy number variations in metabotropic glutamate receptor interacting genes in children with neurodevelopmental disorders. J Neurodev Disord. 2023;15(1):14.

133.	Li YR, Glessner JT, Coe BP, Li J, Mohebnasab M, Chang X, et al. Rare copy number variants in over 100,000 European ancestry subjects reveal multiple disease associations. Nat Commun. 2020;11(1):255.

134.	Sha Z, Sun KY, Jung B, Barzilay R, Moore TM, Almasy L, et al. Copy Number Variant Architecture of Child Psychopathology and Cognitive Development in the ABCD Study. Am J Psychiatry. 2025;182(8):763-78.

135.	Jeng J, Wu Q, Li H. A Statistical Method for Identifying Trait-Associated Copy Number Variants. Hum Hered. 2015;79(3-4):147-56.

136.	Kronenberg ZN, Osborne EJ, Cone KR, Kennedy BJ, Domyan ET, Shapiro MD, et al. Wham: Identifying Structural Variants of Biological Consequence. PLoS Comput Biol. 2015;11(12):e1004572.

137.	Li D, Matsuoka LS, Donoghue S, Hou C, Strong A, McDonald-McGinn DM, et al. Modeling the long-range effect of an inversion downstream of EFNB1 concludes a 43-year molecular diagnostic odyssey for craniofrontonasal syndrome. Eur J Hum Genet. 2025.

138.	Kaivola K, Chia R, Ding J, Rasheed M, Fujita M, Menon V, et al. Genome-wide structural variant analysis identifies risk loci for non-Alzheimer's dementias. Cell Genom. 2023;3(6):100316.

139.	Billingsley KJ, Ding J, Jerez PA, Illarionova A, Levine K, Grenn FP, et al. Genome-Wide Analysis of Structural Variants in Parkinson Disease. Ann Neurol. 2023;93(5):1012-22.

140.	Abe-Hatano C, Iida A, Kosugi S, Momozawa Y, Terao C, Ishikawa K, et al. Whole genome sequencing of 45 Japanese patients with intellectual disability. Am J Med Genet A. 2021;185(5):1468-80.

141.	Larsen SJ, do Canto LM, Rogatto SR, Baumbach J. CoNVaQ: a web tool for copy number variation-based association studies. BMC Genomics. 2018;19(1):369.

142.	da Silva V, Ramos M, Groenen M, Crooijmans R, Johansson A, Regitano L, et al. CNVRanger: association analysis of CNVs with gene expression and quantitative phenotypes. Bioinformatics. 2020;36(3):972-3.

143.	Wu Y, Adams K. Genome-Wide Analyses of Copy Number Variants in 751 Populus trichocarpa Individuals From Natural Populations. Genome Biol Evol. 2025;17(7).

144.	Xie X, Shi L, Hou G, Zhong Z, Wang Z, Pan D, et al. Genome wide detection of CNV and their association with body size in Danzhou chickens. Poult Sci. 2024;103(12):104266.

145.	Wu J, Wu T, Xie X, Niu Q, Zhao Z, Zhu B, et al. Genetic Association Analysis of Copy Number Variations for Meat Quality in Beef Cattle. Foods. 2023;12(21).

146.	Fernandes AC, da Silva VH, Goes CP, Moreira GCM, Godoy TF, Ibelli AMG, et al. Genome-wide detection of CNVs and their association with performance traits in broilers. BMC Genomics. 2021;22(1):354.

147.	Rymuza J, Kober P, Maksymowicz M, Nyc A, Mossakowska BJ, Woroniecka R, et al. High level of aneuploidy and recurrent loss of chromosome 11 as relevant features of somatotroph pituitary tumors. J Transl Med. 2024;22(1):994.

148.	Zhang W, Yang P, Yang Y, Liu S, Xu Y, Wu C, et al. Genomic landscape and distinct molecular subtypes of primary testicular lymphoma. J Transl Med. 2024;22(1):414.

149.	Alinejad-Rokny H, Heng JIT, Forrest ARR. Brain-Enriched Coding and Long Non-coding RNA Genes Are Overrepresented in Recurrent Neurodevelopmental Disorder CNVs. Cell Rep. 2020;33(4):108307.

150.	Labani M, Afrasiabi A, Beheshti A, Lovell NH, Alinejad-Rokny H. PeakCNV: A multi-feature ranking algorithm-based tool for genome-wide copy number variation-association study. Comput Struct Biotechnol J. 2022;20:4975-83.

151.	Beyter D, Ingimundardottir H, Oddsson A, Eggertsson HP, Bjornsson E, Jonsson H, et al. Long-read sequencing of 3,622 Icelanders provides insight into the role of structural variants in human diseases and other traits. Nature Genetics. 2021;53(6):779-86.
